# Iron-Responsive miR-485-3p Regulates Cellular Iron Homeostasis by Targeting Ferroportin

**DOI:** 10.1371/journal.pgen.1003408

**Published:** 2013-04-04

**Authors:** Carolyn Sangokoya, Jennifer F. Doss, Jen-Tsan Chi

**Affiliations:** 1 The Institute for Genome Sciences and Policy, Duke University School of Medicine, Durham, North Carolina, United States of America; 2 Department of Molecular Genetics and Microbiology, Duke University School of Medicine, Durham, North Carolina, United States of America; 3 University Program in Genetics and Genomics, Duke University School of Medicine, Durham, North Carolina, United States of America; University of Heidelberg, Germany

## Abstract

Ferroportin (FPN) is the only known cellular iron exporter in mammalian cells and plays a critical role in the maintenance of both cellular and systemic iron balance. During iron deprivation, the translation of FPN is repressed by iron regulatory proteins (IRPs), which bind to the 5′ untranslated region (UTR), to reduce iron export and preserve cellular iron. Here, we report a novel iron-responsive mechanism for the post-transcriptional regulation of FPN, mediated by miR-485-3p, which is induced during iron deficiency and represses FPN expression by directly targeting the FPN 3′UTR. The overexpression of miR-485-3p represses FPN expression and leads to increased cellular ferritin levels, consistent with increased cellular iron. Conversely, both inhibition of miR-485-3p activity and mutation of the miR-485-3p target sites on the FPN 3′UTR are able to relieve FPN repression and lead to decreased cellular iron levels. Together, these findings support a model that includes both IRPs and microRNAs as iron-responsive post-transcriptional regulators of FPN. The involvement of microRNA in the iron-responsive regulation of FPN offers additional stability and fine-tuning of iron homeostasis within different cellular contexts. MiR-485-3p-mediated repression of FPN may also offer a novel potential therapeutic mechanism for circumventing hepcidin-resistant mechanisms responsible for some iron overload diseases.

## Author Summary

Cellular iron homeostasis is maintained by a sophisticated system that responds to iron levels and coordinates the expression of targets important for balancing iron export and uptake with intracellular storage and utilization. Ferroportin is the only known cellular iron exporter in mammalian cells and plays a critical role in both cellular and systemic iron balance. Thus the ability to regulate cellular iron export is of great interest in the search for therapeutic strategies to control dysregulated iron homeostasis, iron overload disorders, and conditions affected by cellular iron concentrations such as antimicrobial resistance. During iron deprivation, repression of ferroportin levels reduces iron export and preserves cellular iron. Ferroportin translation is known to be repressed by iron regulatory proteins that bind to the 5′UTR, yet alternative mechanisms that can post-transcriptionally regulate ferroportin have not been previously reported. Here, we find that miR-485-3p is induced during iron deficiency and represses ferroportin by directly targeting its 3′UTR, and further experimental evidence supports a model that includes both iron regulatory proteins and microRNAs as post-transcriptional regulators of ferroportin. These findings demonstrate a novel role for microRNAs in the cellular response to iron deficiency and can have therapeutic implications for various diseases of iron homeostasis.

## Introduction

While iron is an essential nutrient for all cells, high levels of iron can lead to toxicity. Therefore, cellular iron homeostasis is carefully maintained by an exquisite system of iron regulatory proteins (IRPs) that respond to iron levels and coordinate the expression of targets important for balancing iron export and uptake with intracellular storage and utilization [1], [2]. Ferroportin (FPN) functions as the only known iron exporter in mammalian cells and plays a critical role in the maintenance of both cellular and systemic iron balance [3]–[5]. Although ubiquitously expressed, FPN is most abundant in cell types known to absorb, process, recycle, and export significant amounts of iron, including duodenal enterocytes, hepatocytes, erythroid cells and reticuloendothelial macrophages [5]–[7].

Given the important regulatory role of FPN, it is not surprising that FPN is regulated at multiple levels–transcriptionally by heme [8], [9], post-transcriptionally by the IRP system [10]–[12], and post-translationally by the iron regulatory hormone hepcidin [13], [14]. During iron deficiency, IRPs inhibit the translation of FPN by binding to the iron regulatory element (IRE) located in the 5′ untranslated region (UTR) of FPN messenger RNA (mRNA), leading to lower FPN protein levels, decreased export of iron, and cellular iron retention [11]. Hepcidin targets membrane-bound FPN for degradation and decreases FPN-mediated iron export. Defects in this FPN ‘off-switch’ as a result of hepcidin deficiency or hepcidin resistance due to FPN gain-of-function mutations can eventually lead to systemic iron overload in the form of hemochromatosis [15]–[18], resulting in significant tissue damage and multi-organ failure with limited therapeutic options [19]. Therefore the identification of novel mechanisms for the post-transcriptional regulation of FPN that can bypass these pathogenic defects will be an important step in the development of novel interventions to ameliorate iron overload and improve clinical outcomes for these patients.

Although there is a greater understanding of transcriptional and IRP-mediated regulation of FPN under various stresses such as heme, nitric oxide, oxidative stress, and hypoxia [8], [9], [20]–, it is not clear whether an IRP-independent mechanism exists for post-transcriptional FPN regulation. One potential class of post-transcriptional regulators are microRNAs— endogenous non-coding small RNAs that bind to complementary sites in the 3′UTR of target mRNAs and drive translational repression or mRNA degradation [23]–[26]. MicroRNAs have been found to play roles as important mediators in various stress responses from flies, worms, and zebrafish to mammals [27]. Recently, the liver-enriched miR-122 has been found to be critical for the control of systemic iron homeostasis in mice by targeting *Hfe* and *Hjv*, which encode proteins important for the hepcidin hormone response to systemic iron availability [28]. While this landmark study focused on miR-122 and captured its role in systemic iron homeostasis, it is unknown whether there are microRNAs that respond to intracellular iron levels and play a role in cellular iron homeostasis, particularly those that can potentially regulate FPN expression.

In this study, we examine the potential role of microRNAs in the IRP/IRE-independent post-transcriptional regulation of FPN. We identify microRNAs with altered expression under cellular iron deprivation and find that the microRNA miR-485-3p directly targets the 3′ UTR of FPN. Through gain-of-function and loss-of-function studies, we provide compelling evidence to support a role for miR-485-3p as an important post-transcriptional regulator of endogenous FPN expression and modulator of cellular iron homeostasis.

## Results

### Evidence of IRP/IRE-independent post-transcriptional regulation of FPN

**Figure 1:**
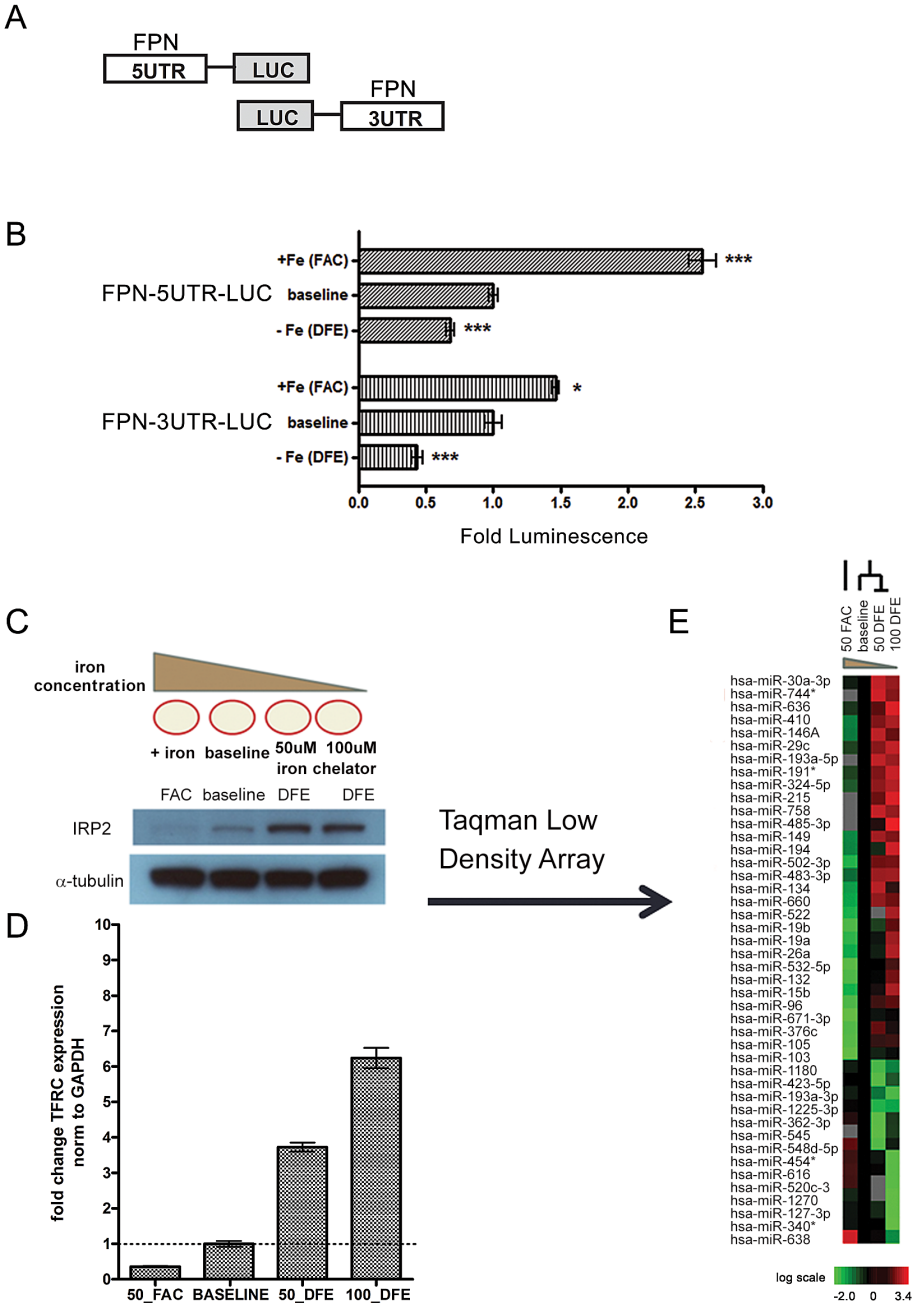
Ferroportin is post-transcriptionally regulated by elements within both the 5′ and 3′ UTRs in response to altered cellular iron concentration. (A) Schematic of FPN-5′UTR and FPN-3′UTR luciferase reporters. (B) The FPN 5′UTR and 3′UTR luciferase reporter constructs were transfected in HepG2 cells and treated with conditions of iron-supplementation (FAC) or iron-depletion (DFE). The relative luciferase activities following iron-supplementation (FAC) or iron-depletion (DFE) are shown when compared with baseline condition. Data is expressed as fold change in luminescence ± SEM relative to baseline condition, normalized to empty reporter control (n = 3). (C) Schematic of iron-supplementation (FAC) or iron-depletion (DFE) of K562 cells to achieve iron-rich and iron-deficient conditions and western blot analysis of IRP2 protein levels in the corresponding conditions. (D) QRT-PCR analysis of TFRC expression from corresponding samples shown in (C), normalized to GAPDH control. (E) Heatmap representation of microRNA expression in cells treated as indicated in (C). The heatmap indicated the change of Ct obtained by the RQ Manager v1.2 which has been normalized to RNU48 endogenous control. MicroRNAs with a log2 expression change of at least 0.5 in either the iron-rich or iron-deficient condition when compared to baseline were selected and considered to be iron-responsive. These microRNAs are filtered and arranged by hierarchical clustering as shown. * Significantly different by Student's t-test: *p<0.05, ***p<0.0001.

To investigate the iron-responsive regulation of the FPN expression, we first demonstrated the known iron-dependent IRP-mediated regulation of the FPN 5′UTR using a luciferase reporter construct with the full FPN 5′UTR placed upstream of luciferase (Figure 1A). The iron-replete and iron-deficient conditions were created by addition of the iron supplement ferric ammonium citrate (FAC) and the iron chelator deferoxamine (DFE), respectively. When normalized to the activity of a control empty reporter under identical treatment, we found that FPN 5′UTR luciferase activity was responsive to iron levels in the human HepG2 hepatocyte cell line— significantly decreased during iron depletion (0.681 fold baseline ±0.029, p<0.0001) and significantly increased (2.554 fold baseline ±0.099, p<0.0001) during iron supplementation (Figure 1B). Similar results were seen in the human K562 human erythroid cell line (Figure S1).

To determine whether the FPN 3′UTR, which lacks IRE, could also be a target of iron-dependent regulation, we used a reporter construct with the full FPN 3′UTR placed downstream of luciferase and analyzed reporter activity in HepG2 cells under different iron conditions. Surprisingly, we found that iron depletion led to significant inhibition of FPN 3′UTR reporter activity (0.437 fold baseline ±0.041, p = 0.012) (Figure 1B). Additionally, iron supplementation led to significant increase (1.463 fold baseline ±0.024, p<0.0001) in FPN 3′UTR reporter activity. Similar results were seen in K562 cells (Figure S1). Since the 3′UTR lacks the IRE region, it is unlikely that these changes are a result of IRP-mediated regulation. Collectively, these data show that both the FPN 5′UTR and 3′UTR can be regulated by iron concentration and indicate an unexpected regulatory role for the 3′ UTR in iron-dependent regulation of FPN.

### Identification of microRNAs responsive to iron concentration

Both RNA-binding proteins and microRNAs are known to function as post-transcriptional regulators via the 3′UTR. To identify microRNAs that could play a role in this regulation, we performed microRNA profiling to identify iron-responsive microRNAs in the K562 erythroid cell line, a well-characterized model for the study of cellular iron metabolism [7], [29], [30]. We treated K562 cells with FAC (iron-rich condition), DFE (iron-deficient condition), or mock (baseline) treatment. Following DFE treatment, we noted increased levels of both IRP2 protein (Figure 1C) and transferrin receptor mRNA (Figure 1D), as expected under iron depletion. We then used quantitative Real-Time PCR Taqman Low Density Arrays (TLDA) to measure the expression of 754 microRNAs under these different iron conditions. Threshold cycle (Ct) values were obtained by the RQ Manager v1.2 software with automatic threshold settings. Of the 300 microRNAs considered to be expressed under these conditions, we identified 44 microRNAs which were differentially expressed from baseline by log2 expression of at least 0.5 in either the iron-deficient or iron-rich condition (Figure 1E).

**Figure 2:**
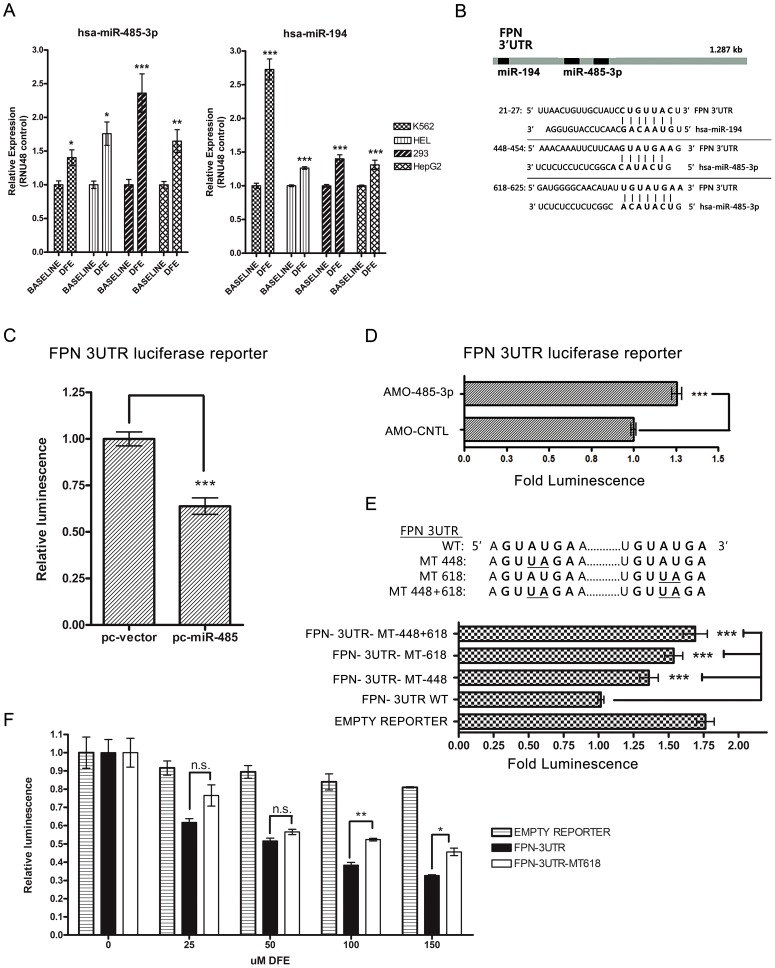
MiR-485-3p directly targets FPN. (A) Quantitative real-time PCR (QRT-PCR) analysis of miR-485-3p (left) and miR-194 (right) expression in K562, HEL, HEK-293, and HepG2 cells after treatment with 100 µM DFE, relative to baseline control. Data expressed as fold change in expression relative to RNU48 control (n = 4). (B) Schematic of the FPN 3′UTR with sequence alignments of predicted miR-194 and miR-485-3p target binding sites. (C) Fold change in luminescence of FPN 3′UTR luciferase reporter in HepG2 cells co-transfected with miR-485 expression construct (pc-miR-485), relative to vector control (pc-vector) (n = 3). (D) Fold change in luminescence of FPN 3′UTR luciferase reporter co-transfected with antisense-mediated oligonucleotides (AMOs) against miR-485-3p (AMO-485-3p), expressed as fold change ± SEM relative to non-targeting control AMO (AMO-CNTL (n = 4). (E) Fold change in luminescence of mutant MT-448+618 FPN 3′UTR, MT-618 FPN 3′UTR, MT-448 FPN 3′UTR, WT 3′UTR and empty luciferase reporter, expressed as fold change ± SEM relative to wild type FPN 3′UTR reporter (n = 4). (F) Activity of FPN 3′UTR, mutant MT-618 FPN 3′UTR, and empty control luciferase reporters following treatment with indicated concentrations of DFE in HepG2 cells. Data is expressed as fold change in luminescence ± SEM relative to baseline condition (0 µM DFE) (n = 3). * Significantly different by Student's t-test: *p<0.05, **p<0.01, ***p<0.0001.

To prioritize the iron-responsive microRNAs with potential regulatory roles in iron homeostasis, we first analyzed the microRNAs (Figure S2A–S2B) with predicted mRNA targets in the cellular iron homeostasis gene ontology (GO: 006879) (Table S1), using the microRNA.org and TargetscanHumanv6.0 databases [31], [32]. Notably, 7/8 repressed and 16/21 induced microRNAs under iron deficiency have predicted iron-related targets (Figure S2A–S2B). Two induced microRNAs, miR-485-3p and miR-194, are predicted to target the FPN 3′UTR (Figure 2B). We used individual TaqMan microRNA Real-time assays to confirm the induction of miR-485-3p, miR-194, and three additional microRNAs (miR-30a*, miR-149, and miR-502-3p) in independent biological replicates in K562 under iron deprivation (Figure 2A and Figure S2C). To determine if these results could be seen in other cell types, we also measured the expression of these microRNAs in response to iron deprivation in HEL (human erythroid), HEK293 (human embryonic kidney), and HepG2 (human hepatocyte) cell lines (Figure 2A and Figure S2C). We found that miR-485-3p exhibited the most uniform and significant induction in response to iron deprivation across all four tested cell lines. To further verify these findings in primary cells, we subjected human primary macrophages to iron depletion and found a similar degree of miR-485-3p induction (Figure S2D).

To date, miR-485-3p has shown only one confirmed target, which is involved in the expression of DNA topoisomerase II in human lymphoblastic leukemia cells [33]. Another study identified an allele variant in functional miR-485-3p target sites of the neurotrophin-3 receptor gene (NTRK3) as a susceptibility factor for anxiety disorders [34]. While most of the targets predicted by TargetScan 6.2 (Table S2) have not been functionally validated, these targets contain many genes involved in G-protein coupled receptor protein signal (GO:0007186), response to external stimulus (GO:0009605) and regulation of metabolism (GO:0019222).

### MiR-485-3p directly targets FPN

The FPN 3′UTR has predicted target sites for both miR-194 and miR-485-3p (Figure 2B). To determine whether the FPN 3′UTR is targeted by these microRNAs, we used expression constructs encoding the precursor hairpin sequences for miR-194 (pc-miR-194) or miR-485 (pc-miR-485) to overexpress miR-194 and miR-485 and measure their respective effects on FPN 3′UTR reporter activity. Enforced miR-485 expression led to significant inhibition of FPN 3′UTR reporter activity (0.639 fold control ±0.044, p<0.001) (Figure 2C and Figure S2E), while miR-194 overexpression did not inhibit (1.186 fold control ±0.05, p = .001) reporter activity (Figure S2E). These data indicate that miR-485-3p, but not miR-194, can act to repress the FPN 3′UTR. We then used antisense-mediated 2′-O-methyl oligonucleotides (AMOs) specific for miR-485-3p to determine the effect of inhibition of endogenous miR-485-3p-mediated RNA-induced silencing complex (RISC) activity [35] on the FPN 3′UTR reporter. Treatment with AMO-485-3p led to significantly increased FPN 3′UTR luciferase reporter activity compared to control in HepG2 (Figure 2D) and K562 (Figure S2F) cells.

Next, we mutated the sequence of the only predicted canonical 8mer miR-485-3p binding site on the FPN 3′UTR, given the high confidence for microRNA-mediated repression with this predicted seed match type [36], and created mutant FPN 3′UTR reporters with mutation in either one predicted site (MT-448 or MT-618) or both predicted sites (MT-448+618) (Figure 2E). While both individual mutations led to significantly increased reporter activities compared to the wild type FPN 3′UTR reporter at baseline (Figure 2E), the change caused by MT-618 (1.536 fold control ±.065, p<0.001) was more than that caused by MT-448 (1.359 fold control ±.066, p<.0001). Mutation of both sites led to even higher (1.690 fold control ±.086, p<.0001) reporter activity (Figure 2E), indicating that both predicted miR-485-3p binding sites contribute significantly to regulation of the FPN 3′UTR. To determine the effect of the predicted miR-485-3p binding sites on the FPN 3′UTR during the iron-deficient state, we measured luciferase activity of the mutant MT-618 FPN 3′UTR reporter compared to the wild type FPN 3′UTR reporter during iron deprivation under increasing concentrations (0–150 µM) of DFE. We found that both the mutant and wild type FPN 3′UTR luciferase reporter activities were decreased under all tested DFE concentrations compared to empty vector control (Figure 2F), however the MT-618 FPN 3′UTR reporter demonstrated significantly higher expression compared to wild type (Figure 2F) under 100 and 150 µM DFE, indicating that this miR-485-3p binding site is a significant contributor in the regulation of the FPN 3′UTR under iron deprivation. Collectively these studies identify FPN as a direct and physiologically relevant target of miR-485-3p.

### MiR-485-3p represses endogenous FPN expression and alters cellular iron status

**Figure 3:**
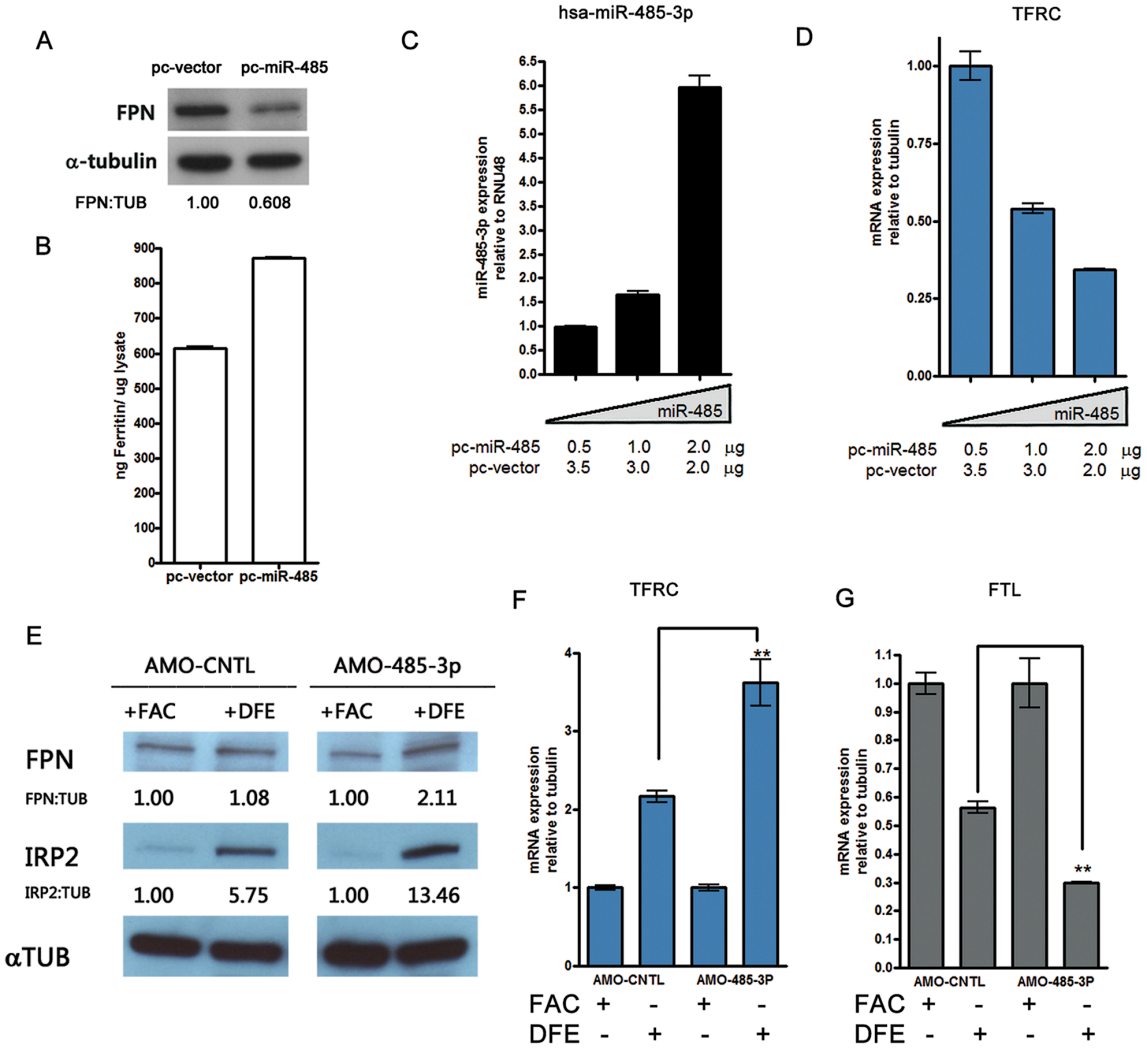
MiR-485-3p-mediated FPN repression is sufficient to alter endogenous cellular iron status. (A) Western blot analysis of FPN and α-tubulin in HepG2 cells transfected with miR-485 (pc-miR-485) or vector control (pc-vector), with densitometric analysis of FPN normalized to tubulin levels. (B) Corresponding ferritin protein levels from samples shown in (3A) as measured by ferritin ELISA. Data are the mean ± SEM (n = 3). (C) QRT-PCR analysis of miR-485-3p expression in HepG2 cells transfected with indicated concentrations of pc-miR-485. Data expressed as fold change in expression relative to RNU48 control (n = 3). (D) QRT-PCR analysis of TFRC mRNA expression in HepG2 cells expressing increasing concentrations of miR-485, relative to tubulin control (n = 3). (E) Western blot analysis of FPN, IRP2 and α-tubulin protein levels in HepG2 cells treated with control (AMO-CNTL) or miR-485-3p-blocking (AMO-485-3p) antisense mediated oligonucleotides and subjected to iron supplementation (FAC) or iron depletion (DFE). Densitometric analysis shows indicated protein expression normalized to tubulin levels, relative to the iron-replete (FAC) condition. (F to G) Corresponding TFRC (F) and FTL (G) mRNA expression, relative to tubulin control, in HepG2 treated with either AMO-CNTL and AMO-485-3p and exposed to iron-rich or iron-deficient condition (n = 3). * Significantly different by Student's t-test: **p<0.01.

Next, we assessed the effect of miR-485-3p on endogenous FPN protein expression and intracellular iron regulation. A previously published FPN antibody [37] identified a ∼68 KDa protein with reduction in intensity following the silencing of FPN via pooled siRNAs (Figure S3A). Enforced expression of miR-485 repressed endogenous FPN protein in both HepG2 (Figure 3A) and K562 (Figure S3B) cells and increased intracellular ferritin levels (Figure 3B and Figure S3C). Transfection with increased concentrations of miR-485 led to dose-dependent increased miR-485-3p expression (Figure 3C and Figure S3D) and corresponding decreases in transferrin receptor (TFRC) mRNA levels (Figure 3D and Figure S3E), consistent with an increase in cellular iron. Importantly, these changes occurred without significant changes in FPN mRNA levels (Figure S3F). Since TFRC is a predicted target of miR-485-3p (Figure S2A and S3G), we tested the potential regulatory relationship using reporter constructs with the wild type (TFRC-3UTR-WT) or with a mutated miR-485-3p binding site (TFRC-3UTR-MT1937). Co-transfection of miR-485-3p did not affect the reporter activities of either reporter constructs (Figure S3H). Therefore, miR-485-mediated changes in TFRC mRNA are likely secondary to the changes in the cellular iron status instead of a result of direct regulation.

The specific inhibition of miR-485-3p activity in HepG2 cells by AMOs led to significant and reproducible increase in FPN protein levels in response to iron depletion (Figure 3E and Figure S3I–S3J). We demonstrate that these cells with loss of miR-485-3p function are in a greater state of iron deficiency, as evidenced by increased levels of IRP2 protein (Figure 3E), increased TFRC mRNA expression (Figure 3F), and decreased ferritin light chain (FTL) mRNA expression (Figure 3G). The observed increase in the expression of ferroportin despite increased IRP2 protein level suggests that miR-485-3p activity is necessary for the response of FPN expression to iron depletion. This observation is consistent with the possibility that the microRNA activity of miR-485-3p plays an important role that is separate and distinct from the regulation by IRP2. Collectively, these gain-of-function and loss-of-function data strongly support a role of miR-485-3p as an important post-transcriptional regulator of endogenous FPN expression.

### Both IRP-mediated 5′UTR regulation and miR-485-3p-mediated FPN 3′UTR regulation contribute to overall post-transcriptional regulation of FPN

**Figure 4:**
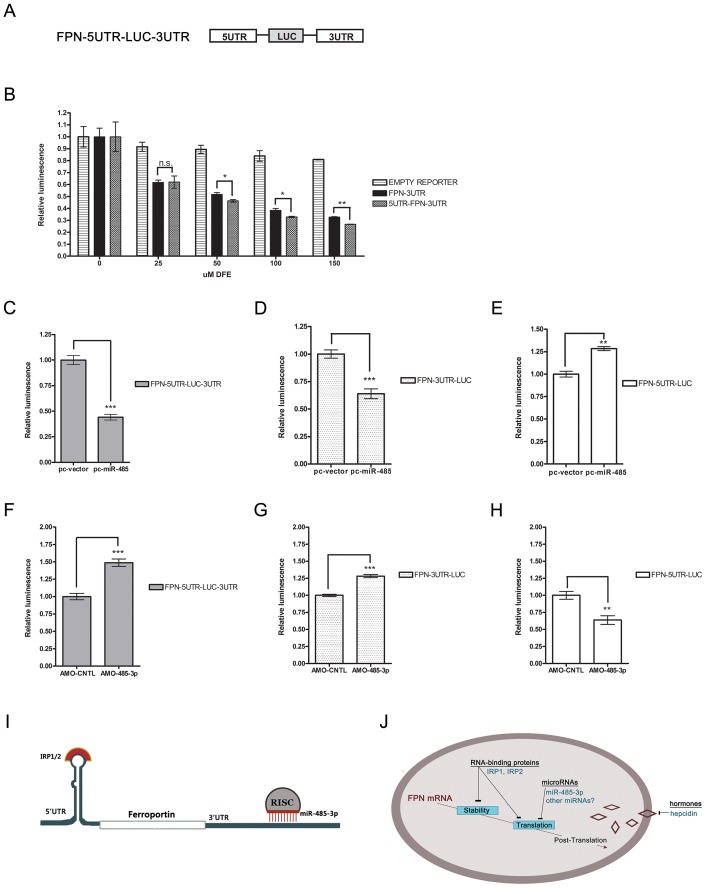
Both IRP-mediated 5′UTR and MiR-485-3p-mediated FPN 3′UTR to post-transcriptional regulation of FPN. (A) Schematic representation of FPN-5UTR-LUC-3UTR luciferase reporter construct. (B) Activity of FPN 3′UTR, 5UTR-LUC-3UTR, and empty control luciferase reporters in HepG2 cells following treatment with indicated concentrations of DFE. Data is expressed as fold change in luminescence ± SEM relative to baseline condition (0 µM DFE) (n = 3). (C–E) Activity of FPN 5′UTR reporter (C), FPN-5UTR-LUC-3UTR reporter (D), and FPN 3′UTR reporter (E) following overexpression of miR-485 (pc-miR-485) or vector control (pc-vector); expressed as fold change in luminescence ± SEM relative to control samples (n = 4). (F–H) Activity of FPN 5′UTR reporter (F), FPN-5UTR-LUC-3UTR reporter (G), and FPN 3′UTR reporter (H) following treatment with control (AMO-CNTL) or miR-485-3p-blocking (AMO-485-3p) antisense mediated oligonucleotides; expressed as fold change in luminescence ± SEM relative to AMO-CNTL samples. Results of reporter experiments in (C–H) are normalized to empty reporter controls subjected to identical treatment (n = 3). (I) Model of IRP and miR-485-3p-mediated regulation of the FPN transcript during the iron-deficient condition. (J) Model of post-transcriptional regulation of ferroportin mRNA. * Significantly different by Student's t-test: *p<0.05, **p<0.01, ***p<0.0001.

Finally we sought to mimic the regulation of endogenous FPN mRNA by constructing a luciferase reporter (FPN-5UTR-LUC-3UTR) containing both the FPN 5′UTR and FPN 3′UTR placed upstream and downstream of luciferase, respectively (Figure 4A). We measured luciferase reporter activity of the FPN-5UTR-LUC-3UTR reporter compared to the FPN 3′UTR reporter during iron deprivation under increasing concentrations (0–150 µM) of DFE (Figure 4B). When normalized to control empty reporter under identical conditions, we found that although both reporters exhibited significantly decreased activities under all tested DFE concentrations, the effect of iron deprivation on the FPN-5UTR-LUC-3UTR reporter was slightly, but significantly more decreased under 50, 100, and 150 µM DFE compared with that of the FPN 3′UTR reporter.

We used the FPN-5UTR-LUC-3UTR, FPN-5′UTR, and FPN 3′UTR reporters to further characterize the effect of both regulatory regions on post-transcriptional regulation of FPN in response to miR-485 overexpression (Figure 4C–4E) or inhibition of miR-485-3p activity (Figure 4F–H). Enforced expression of miR-485 significantly inhibited both the FPN-5UTR-LUC-3UTR and FPN 3′UTR reporters, leading to 0.442 fold control (±.027, p<0.0001) and 0.639 fold control (±.044, p<0.0001) reporter activities, respectively (Figure 4C–4D). Enforced expression of miR-485 significantly increased FPN 5′UTR activity (1.283 fold control ±.022, p = 0.0004) (Figure 4E). Since the FPN 5′UTR contains the IRP binding site and does not have predicted miR-485 target sites, this increase likely reflects the increased endogenous cellular iron retention due to miR-485-mediated decreased endogenous FPN levels. Inhibition of miR-485-3p-mediated RISC activity by AMO-485-3p led to an increased activity of the FPN 5UTR-LUC-3UTR (1.488 fold control ±.053, p<0.0001) and FPN 3′UTR (1.282 fold control ±.023, p<0.0001) reporters compared to control inhibitor (Figure 4F–4G). Treatment with AMO-485-3p led to significantly decreased FPN 5′UTR activity (0.638 fold control ±.064, p = 0.010) (Figure 4H). With the AMO-485-3p-mediated potentiation of FPN levels and the subsequent continued export of iron, this decrease likely reflects the binding of IRPs in response to decreased endogenous cellular iron levels.

In summary, we demonstrate the post-transcriptional regulation of FPN during the iron-deficient condition by miR-485-3p via the 3′UTR in addition to the well-recognized regulation by IRPs via the 5′UTR (Figure 4I). These findings support a model that includes both IRPs and miR-485-3p as concurrent modulators of mRNA stability and translation in the post-transcriptional regulation of FPN expression (Figure 4J) and in the fine-tuning of cellular iron homeostasis.

## Discussion

Given the crucial role of FPN in iron metabolism, extensive regulation of FPN occurs at multiple levels, including the transcriptional [8], [9], [38], post-transcriptional [10]–[12], and post-translational (hepcidin) levels [13], [14], [16], [39]. This study, for the first time, establishes the 3′UTR of FPN as an important regulatory region and miR-485-3p as a post-transcriptional regulator in response to iron deprivation to reduce FPN expression and iron export in the maintenance of cellular iron homeostasis.

The discovery of iron-responsive microRNAs and microRNA-mediated regulation of FPN in several cell lines and primary macrophages illustrates the complexity of regulatory mechanisms for the precise and dynamic regulation of cellular iron. However, these findings were mainly obtained from the cellular response to varying iron levels *in vitro*. The use of primary macrophages and several cell types studied, and their longstanding use in this field offers a broad baseline and physiologically relevant context to indicate the potential relevance of individual microRNAs and their functional target(s) in cellular iron regulation. But it will be important to further establish the *in vivo* relevance of these findings using clinical samples from individuals with iron overload and iron deficiency conditions or receiving treatments to correct conditions of iron deficiency or overload.

Several microRNAs have been found to regulate targets with key roles in iron homeostasis. The hypoxia-induced miR-210 is known to directly target ISCU1/2, which play a role in the biogenesis and integrity of iron-sulfur clusters [40]. Repression of iron-sulfur clusters increases the functionality of IRP1 as an RNA-binding protein and indirectly alters IRP1-dependent regulation [40], [41]. The liver-specific microRNA miR-122 is known to directly target *Hjv* and *Hfe*, both important for hepcidin expression, and has been shown to play an important role in the control of murine systemic iron homeostasis [28]. However, no studies to date have sought to identify a potential repertoire of microRNAs whose expression levels are associated with changes in cellular iron concentration in mammalian cells. Using an unbiased approach, we have identified iron-responsive microRNAs with predicted mRNA targets associated with cellular iron homeostasis. These microRNAs are expected to play an integral role in the cellular iron response.

The regulation of FPN by microRNAs is likely to be distinct from other well-established mechanisms in several important ways. Unlike the systemic regulation of FPN by circulating hormone hepcidin, the monitoring of local iron levels by cellular microRNAs can lead to a more dynamic response to spatial and temporal fluctuations. While both microRNAs and IRPs are iron-responsive and target a group of mRNAs, they may also respond to different sets of non-iron environmental conditions and regulate distinct sets of target mRNAs to allow for diversity and fine-tuning of gene regulation. The targeting of FPN by microRNAs in the 3′ UTR allows for the possibility of iron-dependent regulation of subsets of FPN mRNAs known to lack the 5′ UTR [42], [43]. Given that the specific composition of microRNAs can differ among cell types, distinct and coordinated responses of iron-responsive networks may exist within different cell types. Thus, it will be important to extend this study to other relevant cell types and validate the interaction of iron-responsive microRNAs with predicted iron-related targets. Additionally, high-throughput techniques to probe the microRNA-mRNA interactome [44], [45] offer powerful complementary approaches to identify the *in vivo* target mRNAs associated with Ago2 during different iron states. Such exploration of iron-responsive microRNAs and their respective targets will lead to a more comprehensive pathway demonstrating an integrated role for microRNAs in the regulation of cellular iron homeostasis.

Since FPN is known to be repressed by the IRP/IRE system under the iron-deficient condition, our findings suggest a potential cooperative relationship between RNA-binding proteins (RBPs) and microRNAs in the regulation of FPN. The cooperative contribution of RBPs, including both IRPs and the microRNA-guided RISC, to the post-transcriptional regulation of target RNAs constitutes a major regulatory layer of gene expression [46], [47]. RBPs can function to promote or inhibit microRNA target availability and binding, leading to the enhancement or inhibition of mRNA stability and translation [27]. In the case of FPN, it is possible that the IRP/IRE 5′UTR interaction can be further stabilized and fine-tuned by the microRNA-mediated RISC on the 3′UTR to enable a more dynamic and fine-tuned expression over a wide range of iron conditions.

Finally, microRNA-mediated regulation by miR-485-3p may offer a novel alternative means to target intracellular FPN and alter cellular iron status. Successful proof-of-concept studies supporting the use of therapeutic microRNA mimics have been demonstrated with microRNAs identified as functional tumor suppressors in mouse models of cancer [48]–[50]. Therapeutic inhibition of miR-122 with locked nucleic acid–modified oligonucleotides [51] has recently been shown to successfully lower hepatitis C virus (HCV) replication in chronically infected primate models and lead to long-lasting suppression of HCV viremia and improvement of HCV-induced liver pathology [52]. Manipulation of the miR-485-3p-FPN regulatory axis can potentially be used as a tool to bypass hepcidin deficiency or hepcidin resistance due to FPN gain-of-function mutations, mechanisms that lead to systemic iron overload pathology. Since FPN expression and cellular iron levels can control the growth of *Salmonella* [53], [54], the miR-485-3p-FPN relationship may also prove relevant for antimicrobial resistance strategies.

## Materials and Methods

### Cell culture and *in vitro* regulation of cellular iron

K562 cells were maintained in RPMI; 293 and HepG2 cells were maintained in DMEM. All cells were incubated in humidified atmosphere of 5% CO2 at 37°C and supplemented with 10% fetal bovine serum (HyClone) and 1% penicillin and streptomycin. Ferric ammonium citrate (FAC) and deferoxamine (DFE) were purchased from Sigma. For iron depletion, cells were treated with DFE (100 µM unless otherwise indicated) diluted in PBS and added to the media for indicated time intervals (16–24 hours). For iron supplementation, cells were treated with FAC (500 nM) diluted in PBS and added to the media for indicated time intervals (16–24 hours).

### Purification and differentiation of primary human macrophages

Human peripheral blood mononuclear cells (PBMC) were isolated by Ficoll Paque (GE healthcare) density centrifugation from whole blood. Monocytes were enriched from freshly isolated PBMC through plastic adherence for 1–2 hours. To differentiate monocytes into macrophages, cells were plated into RPMI 1640 media with 2 mM glutamine (Gibco) containing 10% fetal bovine serum (Hyclone), 100 µg/ml streptomycin, 100 U/ml penicillin, 1% Na-pyruvate, 1% NEAA (Non-Essential Amino Acids) and 50 ng/ml rHu M-CSF. Cells were allowed to differentiate over a course of seven days, and then treated.

### Reporter construction, cell transfection, and analysis

Luciferase reporters were constructed using the psi-CHECK2 vector (Promega). The 5′UTR of FPN was amplified using primers (forward: CAGCTAGCCCGACTCGGTATAAGAGCTG; reverse: CAGCTAGCAACAGGAGTGCAAGGAACTG) and cloned upstream of Renilla luciferase to form FPN-5UTR-LUC. The 3′UTR of FPN amplified using primers (forward: TTTAACTGTTGCTATCCTGTTACT; reverse: CCTTTTTACAAAGATTTTACAACATAG) and cloned into the MCS downstream of Renilla luciferase (FPN-3UTR-LUC). The 3′UTR of TFRC amplified using primers (forward: GCAAAATGCATGCCCTGTA; reverse: AAGCATTGGGTGGGTAAATTC) and cloned into the MCS downstream of Renilla luciferase (TFRC-3UTR-LUC).

Mutant reporters were constructed using primer-based overlapping PCR with the following primers: FPN3UTRmt448-F- TGCATCTTAGTTATTTTTAAAAACAAATTCTTCAAGTTAGAAGACTAAATTTTGATAACTAATATTATCCTTATTG, FPN3UTRmt448-R CAATAAGGATAATATTAGTTATCAAAATTTAGTCTTCTAACTTGAAGAATTTGTTTTTAAAAATAACTAAGATGCA, FPN3UTRmt618-F- ACATCAAGAGCTTCGTGGAG, FPN3UTRmt618-R- CTCGAGTTACAAAGATTTTACAACATAG, FPN3UTRmt618-mutF- GGCAACATATTTGTTAGAAGCA, FPN3UTRmt618-mutR- CTAACAAATATGTTGCCCCCATC, TFRC3UTRmt1937-F-GATGGTTCACTCACGGAGCTTCGAACTTATTGTAACCTACATTTAATTGATC, TFRC3UTRmt1937-R-GATCAATTAAATGTAGGTTACAATAAGTTCGAAGCTCCGTGAGTGAACCATC. All reporters were transfected into K562 and HepG2 cells using Lipofectamine 2000 or Lipofectamine LTX (Invitrogen). Reporter assays were conducted using the Dual Luciferase Reporter Assay System (Promega) and the Tecan Infinite F200 reader according to manufacturer's protocol.

### Expression vectors, antisense-mediated oligonucleotides, and cell transfection

Expression constructs encoding miR-485 (pc-miR-485) and miR-194 (pc-miR-194) were created by insertion into a cytomegalovirus-based pcDNA3 cloning vector (Invitrogen) using the following primers: pc-miR-485-forward:TCATGTGTGGTACTTGGAGA; pc-miR-485-reverse: AAAAGAAGTCAGCCATGTGT; pc-miR-194-forward: GAATTCCCATGATGAGCAAAAGGAATC; pc-miR-194-reverse: CTCGAGATCAAAAGTAACAGCATCTC; Antisense-2′O-methyl-modified nucleotides were purchased from (Dharmacon). AMO-485-3p: AGAGAGGAGAGCCGTGTATGAC; AMO-CNTL1: AAGGCAAGCUGACCCUGAAGU; AMO-CNTL2: CCAUCUUUACCAGACAGUGUUA. Control (Ambion) and FPN siRNA (SMARTpool from Dharmacon) were transfected into K562 and HepG2 cells using nucleofection (Amaxa) and lipofection (Invitrogen) methods.

### Real-time quantitative RT–PCR and microRNA expression analysis

Quantitative real-time RT-PCR (qRT-PCR) analysis of microRNA expression using TaqMan Low Density Arrays (TLDA) Human microRNA Panel (Applied Biosystems) was conducted according to manufacturer's instructions using the ABI 7500 real-time PCR system (Applied Biosystems). The Ct data were obtained by the RQ Manager v1.2 software using automatic threshold settings and normalized to RNU48 endogenous control. MicroRNAs with a log2 expression change of at least 0.5 in either the iron-rich or iron-deficient condition when compared to baseline were considered to be iron-responsive. Individual qRT-PCR Taqman mature microRNA assays (Applied Biosystems) were used for validation of results from TLDA. qRT-PCR analysis of mRNA expression using Power SYBR Green (Applied Biosystems) was conducted as described previously [55] with primers specific for ferroportin, transferrin, and ferritin light chain, with beta-tubulin as an internal control.

### Western blot analysis, antibodies, and Ferritin ELISA

Western blots were performed as described in [55] using the following antibodies: IRP2 (Santa Cruz Biotechnology), alpha tubulin (Sigma), GAPDH (Santa Cruz Biotechnology), anti-mouse IgG-HRP (R&D Systems) and anti-rabbit IgG-HRP (R&D Systems). The FPN antibody was kindly provided by Dr. Tomasa Barrientos de Renshaw of the Andrews Laboratory as described [37]. Relevant protein band was identified by performing siRNA experiments using control (Ambion) or SMARTpool FPN-targeting siRNA (Dhamarcon). Ferritin ELISA was performed using the Human Ferritin ELISA assay (Abnova) according to manufacturer's instructions.

### Statistical analysis

Statistical analyses were performed using the Student's *t* test. Results were considered statistically significant at a p-value <0.05 (*), <0.01 (**), or <.0001 (***). n.s = nonsignificant. Graphs were generated using Prism 5 software (GraphPad software, Inc.)

### Primers

QPCR-Beta tubulin-F- GCACATAGTAGGCGCTCAAT

QPCR-Beta tubulin-R- ATCTGGAGACCCAGCTTCTT

QPCR-FPN-F- GACATGAGCAAGAGCCTAC

QPCR-FPN-R- AGGCTGGTTGTAGTAGGAGA

QPCR-TFRC-F- AAAATCCGGTCTAGGCACAG

QPCR-TFRC-R- CCTTTAAATGCAGGGACGAA

QPCR-FTL-F- GGGTCTGTCTCTTGCTTCAAC

QPCR-FTL-R- GGTTGGCAAGAAGGAGCTAA

QPCR-GAPDH-F- AGCAAGAGCACAAGAGGAAG

QPCR-GAPDH-R- GGTTGAGCACAGGGTACTTT

## Supporting Information

Figure S1 Activity of FPN 5′UTR and 3′UTR luciferase reporters in K562 cells following iron-supplementation (FAC) or iron-depletion (DFE). Data expressed as fold change in luminescence ± SEM relative to baseline condition, normalized to empty reporter control (n = 3). * Significantly different by Student's t-test: **p<0.01, ***p<0.0001.
(PDF)

Figure S2 (A–B) Predicted iron-relevant mRNA targets of significantly upregulated (A) and downregulated. (B) microRNAs (listed in Table S1). (C) Quantitative real-time PCR (QRT-PCR) analysis of miR-149, miR-502-3p, and miR-30a* expression in K562, HEL, and HEK-293 cells after treatment with 100 µM deferoxamine (DFE), relative to baseline control. Data expressed as fold change in expression relative to RNU48 control (n = 4). (D) Quantitative real-time PCR (QRT-PCR) analysis of miR-485-3p expression in human primary macrophages after treatment with 100 µM deferoxamine (DFE), relative to baseline control. Data expressed as fold change in expression relative to RNU48 control (n = 6). (E) Fold change in luminescence of FPN 3′UTR luciferase reporter in K562 cells co-transfected with miR-485 expression construct (pc-miR-485) (left) or miR-194 (pc-miR-194), expressed as fold change ± SEM relative to vector control (pc-vector) (n = 3). (F) Fold change in luminescence of FPN 3′UTR luciferase reporter co-transfected with antisense-mediated oligonucleotides (AMOs) against miR-485-3p (AMO-485-3p) expressed as fold change ± SEM relative to control AMOs (AMO-CNTL1, AMO-CNTL2)(n = 3). * Significantly different by Student's t-test: *p<0.05, **p<0.01, ***p<0.0001.
(PDF)

Figure S3 (A) Western blot confirmation of antibody specificity by silencing of FPN with siRNAs. HepG2 lysate from cells transfected with control or FPN-targeting siRNAs were analyzed by PAGE and probed with FPN antibody. One indicated ∼68 KDa band was recognized as FPN protein by its reduction in intensity following the silencing of FPN. (B) Western blot analysis of FPN in K562 cells transfected with miR-485 (pc-miR-485) or vector control (pc-vector), normalized to tubulin levels. (C) Ferritin protein levels from corresponding samples in Figure S3A as measured by ferritin ELISA. Data are the mean ± SEM (n = 3). (D) QRT-PCR analysis of miR-485-3p expression in K562 cells transfected with increasing concentration of pc-miR-485. Data expressed as fold change in expression relative to RNU48 control (n = 3). (E to F) QRT-PCR analysis of TFRC (E) and FPN (F) mRNA expression in K562 cells expressing increasing concentrations of miR-485, relative to tubulin control (n = 3). (G) Schematic of the TFRC 3′UTR with sequence alignments of predicted miR-485-3p target binding site. (H) Fold change in luminescence of TFRC 3′UTR luciferase reporter in HepG2 cells co-transfected with miR-485 expression construct (pc-miR-485) or vector control construct (pc-vector), expressed as fold change ± SEM relative to vector control (pc-vector) co-transfected with empty reporter control (n = 4). None of the pairwise comparisons were statistically significant. (I) Full western blot and molecular size markers from Figure 3E. (J) Quantitation of FPN protein expression in HepG2 cells transfected with control (AMO-CNTL) or miR-485-3p-blocking (AMO-485-3p) antisense mediated oligonucleotides and subjected to iron supplementation (FAC) or iron depletion (DFE) (n = 3). * Significantly different by Student's t-test: *p<0.05.
(PDF)

Table S1 List of genes involved in cellular iron ion homeostasis from gene ontology (GO: 006879) analysis, using the microRNA.org and TargetscanHumanv6.0 databases.
(PDF)

Table S2 A list of miR-485-3p target mRNAs predicted by Targetscan.
(PDF)
