# Supplementary figures and images for: Iron-Responsive miR-485-3p Regulates Cellular Iron Homeostasis by Targeting Ferroportin
Source: PLoS Genet. 2013 Apr 4;9(4):e1003408. doi: 10.1371/journal.pgen.1003408 (PMC3616902; doi:10.1371/journal.pgen.1003408)

Supplemental Figure 1

S1

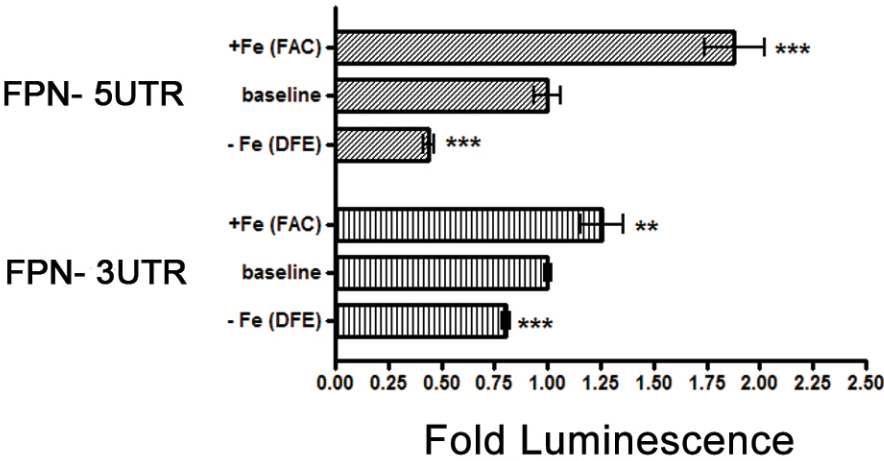

Supplement: Figure S1 — Activity of FPN 5′UTR and 3′UTR luciferase reporters in K562 cells following iron-supplementation (FAC) or iron-depletion (DFE). Data expressed as fold change in luminescence ± SEM relative to baseline condition, normalized to empty reporter control (n = 3). * Significantly different by Student's t-test: **p<0.01, ***p<0.0001. (PDF) [file pgen.1003408.s001.pdf]

Supplemental Figure 3

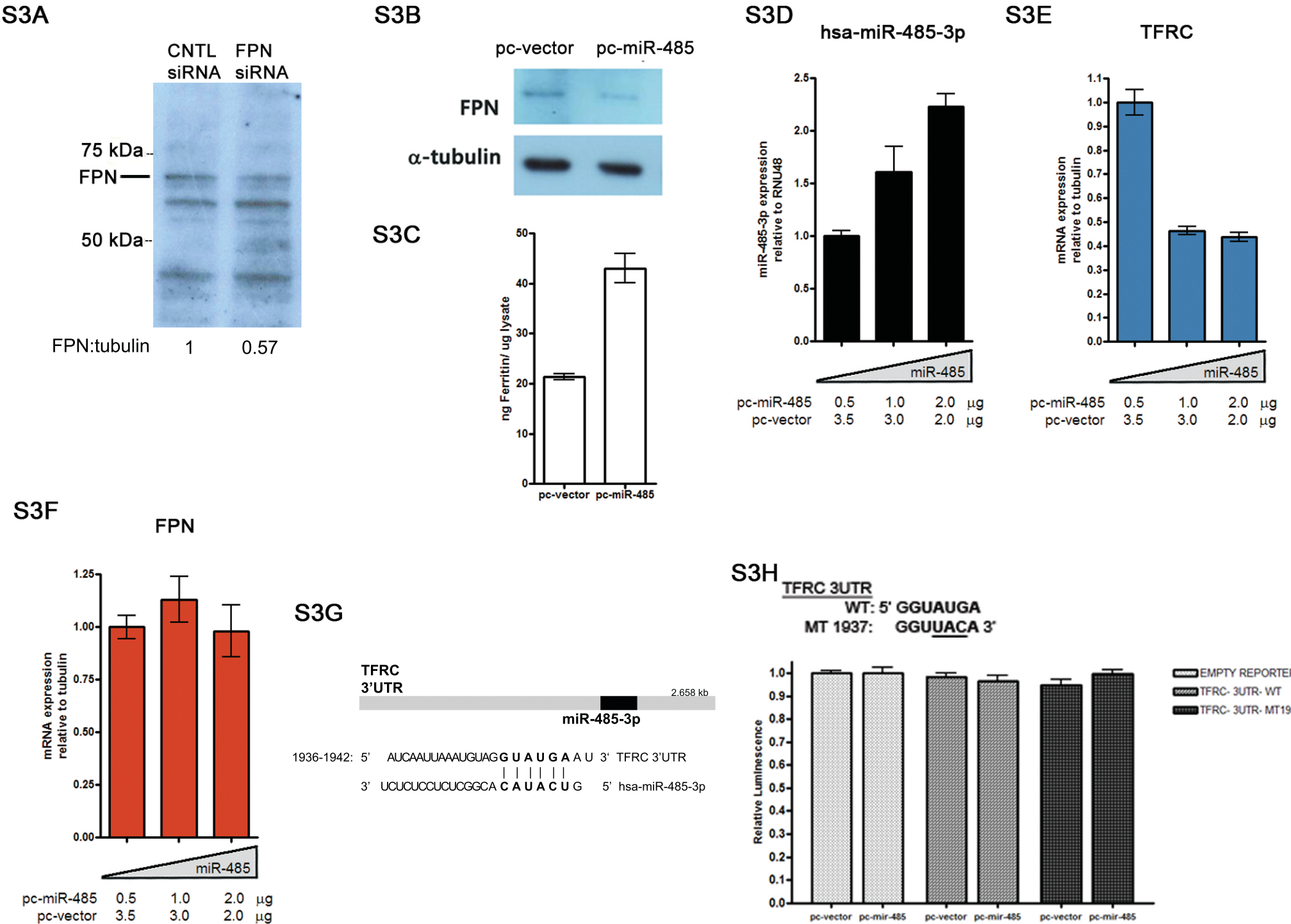

S3I

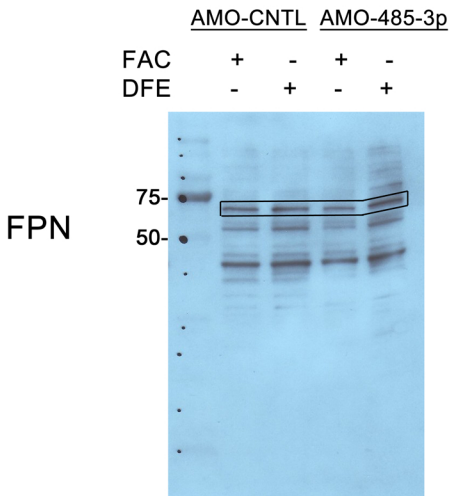

S3J

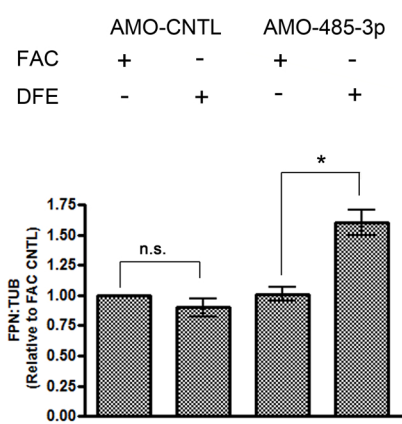

Supplement: Figure S3 — (A) Western blot confirmation of antibody specificity by silencing of FPN with siRNAs. HepG2 lysate from cells transfected with control or FPN-targeting siRNAs were analyzed by PAGE and probed with FPN antibody. One indicated ∼68 KDa band was recognized as FPN protein by its reduction in intensity following the silencing of FPN. (B) Western blot analysis of FPN in K562 cells transfected with miR-485 (pc-miR-485) or vector control (pc-vector), normalized to tubulin levels. (C) Ferritin protein levels from corresponding samples in Figure S3A as measured by ferritin ELISA. Data are the mean ± SEM (n = 3). (D) QRT-PCR analysis of miR-485-3p expression in K562 cells transfected with increasing concentration of pc-miR-485. Data expressed as fold change in expression relative to RNU48 control (n = 3). (E to F) QRT-PCR analysis of TFRC (E) and FPN (F) mRNA expression in K562 cells expressing increasing concentrations of miR-485, relative to tubulin control (n = 3). (G) Schematic of the TFRC 3′UTR with sequence alignments of predicted miR-485-3p target binding site. (H) Fold change in luminescence of TFRC 3′UTR luciferase reporter in HepG2 cells co-transfected with miR-485 expression construct (pc-miR-485) or vector control construct (pc-vector), expressed as fold change ± SEM relative to vector control (pc-vector) co-transfected with empty reporter control (n = 4). None of the pairwise comparisons were statistically significant. (I) Full western blot and molecular size markers from Figure 3E. (J) Quantitation of FPN protein expression in HepG2 cells transfected with control (AMO-CNTL) or miR-485-3p-blocking (AMO-485-3p) antisense mediated oligonucleotides and subjected to iron supplementation (FAC) or iron depletion (DFE) (n = 3). * Significantly different by Student's t-test: *p<0.05. (PDF) [file pgen.1003408.s003.pdf]
