# Supplementary material for: Iron-Responsive miR-485-3p Regulates Cellular Iron Homeostasis by Targeting Ferroportin
Source: PLoS Genet. 2013 Apr 4;9(4):e1003408. doi: 10.1371/journal.pgen.1003408 (PMC3616902; doi:10.1371/journal.pgen.1003408)

Supplemental Figure 2

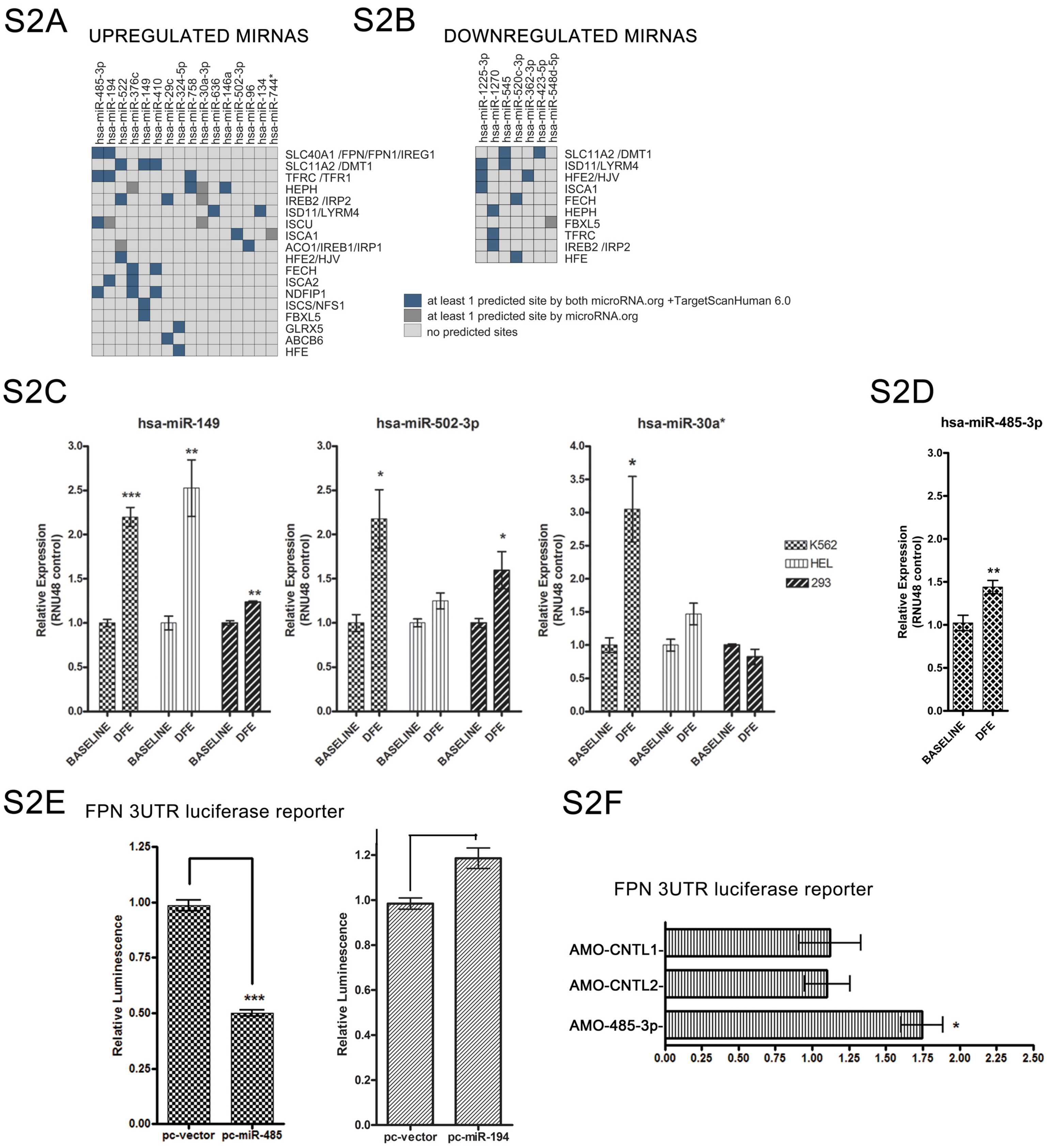

Supplement: Figure S2 — (A–B) Predicted iron-relevant mRNA targets of significantly upregulated (A) and downregulated. (B) microRNAs (listed in Table S1). (C) Quantitative real-time PCR (QRT-PCR) analysis of miR-149, miR-502-3p, and miR-30a* expression in K562, HEL, and HEK-293 cells after treatment with 100 µM deferoxamine (DFE), relative to baseline control. Data expressed as fold change in expression relative to RNU48 control (n = 4). (D) Quantitative real-time PCR (QRT-PCR) analysis of miR-485-3p expression in human primary macrophages after treatment with 100 µM deferoxamine (DFE), relative to baseline control. Data expressed as fold change in expression relative to RNU48 control (n = 6). (E) Fold change in luminescence of FPN 3′UTR luciferase reporter in K562 cells co-transfected with miR-485 expression construct (pc-miR-485) (left) or miR-194 (pc-miR-194), expressed as fold change ± SEM relative to vector control (pc-vector) (n = 3). (F) Fold change in luminescence of FPN 3′UTR luciferase reporter co-transfected with antisense-mediated oligonucleotides (AMOs) against miR-485-3p (AMO-485-3p) expressed as fold change ± SEM relative to control AMOs (AMO-CNTL1, AMO-CNTL2)(n = 3). * Significantly different by Student's t-test: *p<0.05, **p<0.01, ***p<0.0001. (PDF) [file pgen.1003408.s002.pdf]
