# Supplementary material for: Iron-Responsive miR-485-3p Regulates Cellular Iron Homeostasis by Targeting Ferroportin
Source: PLoS Genet. 2013 Apr 4;9(4):e1003408. doi: 10.1371/journal.pgen.1003408 (PMC3616902; doi:10.1371/journal.pgen.1003408)
Supplement: Table S1 — List of genes involved in cellular iron ion homeostasis from gene ontology (GO: 006879) analysis, using the microRNA.org and TargetscanHumanv6.0 databases. (PDF) [file pgen.1003408.s004.pdf]

Genes involved in cellular iron ion homeostasis

GENE	NAME
FECH	Ferrochelatase, mitochondrial
ISCU	iron-sulfur cluster assembly enzyme
TFRC /TFR1	Transferrin receptor protein 1
ISCA1	Iron-sulfur cluster assembly 1 homolog, mitochondrial
ISCS/NFS1	NFS1 nitrogen fixation 1 homolog
ISD11/LYRM4	LYR motif containing 4
SLC40A1 /FPN/FPN1/IREG1	Solute carrier family 40 member 1
ACO1/IREB1/IRP1	Cytoplasmic aconitate hydratase
ALAS2 /ALASE	5-aminolevulinate synthase, erythroid-specific, mitochondrial
FBXL5	F-box/LRR-repeat protein 5
GLRX5	Glutaredoxin-related protein 5, mitochondrial
ISCA2	Iron-sulfur cluster assembly 2 homolog, mitochondrial
SLC11A2 /DMT1	Natural resistance-associated macrophage protein 2
ABCB6	ATP-binding cassette sub-family B member 6, mitochondrial
ABCB7	ATP-binding cassette sub-family B member 7, mitochondrial
FTH1 /FTH	Ferritin heavy chain
IREB2 /IRP2	Iron-responsive element-binding protein 2
ABCG2	ATP-binding cassette sub-family G member 2
CP	Ceruloplasmin
CYBRD1	Cytochrome b reductase 1
FLVCR1	Feline leukemia virus subgroup C receptor-related protein 1
FTH1P19	Putative ferritin heavy polypeptide-like 19
FTHL17	Ferritin heavy polypeptide-like 17
FTL	Ferritin light chain
FTMT	Ferritin, mitochondrial
FXN	Frataxin, mitochondrial
GDF2/BMP9	Growth/differentiation factor 2
HAMP	Hepcidin
HEPH	Hephaestin
HFE	Hereditary hemochromatosis protein
HMOX1 /HO1	Heme oxygenase 1
HMOX2 /HO2	Heme oxygenase 2
HN/MT-RNR2	Putative humanin peptide
HP	Haptoglobin
HPX	Hemopexin
LTF	Lactotransferrin
MCOLN1	Mucolipin-1
MFI2	Melanotransferrin
MYC	Myc proto-oncogene protein
NDFIP1	NEDD4 family-interacting protein 1
NUBP1	Cytosolic Fe-S cluster assembly factor NUBP1
SCARA5	Scavenger receptor class A member 5
SLC11A1/NRAMP	Natural resistance-associated macrophage protein 1
SLC46A1	Proton-coupled folate transporter
SOD1	Superoxide dismutase [Cu-Zn]
STEAP3	Metalloreductase STEAP3
TCIRG1	V-type proton ATPase 116 kDa subunit a isoform 3
TF	Serotransferrin
TFR2	Transferrin receptor protein 2
