# Supplementary material for: Iron-Responsive miR-485-3p Regulates Cellular Iron Homeostasis by Targeting Ferroportin
Source: PLoS Genet. 2013 Apr 4;9(4):e1003408. doi: 10.1371/journal.pgen.1003408 (PMC3616902; doi:10.1371/journal.pgen.1003408)
Supplement: Table S2 — A list of miR-485-3p target mRNAs predicted by Targetscan. (PDF) [file pgen.1003408.s005.pdf]

Supplemental Table 2

| Target gene | Representative transcript | Gene name |
|---|---|---|
| CCDC68 | NM_001143829 | coiled-coil domain containing 68 |
| WDR35 | NM_001006657 | WD repeat domain 35 |
| PM20D2 | NM_001010853 | peptidase M20 domain containing 2 |
| PDE10A | NM_001130690 | phosphodiesterase 10A |
| NOM1 | NM_138400 | nucleolar protein with MIF4G domain 1 |
| CPOX | NM_000097 | coproporphyrinogen oxidase |
| SOX7 | NM_031439 | SRY (sex determining region Y)-box 7 |
| C17orf75 | NM_022344 | chromosome 17 open reading frame 75 |
| HCN1 | NM_021072 | hyperpolarization activated cyclic nucleotide-gated potassium channel 1 |
| ARHGAP20 | NM_020809 | Rho GTPase activating protein 20 |
| TRIM2 | NM_001130067 | tripartite motif containing 2 |
| ZMYM4 | NM_005095 | zinc finger, MYM-type 4 |
| ZNF10 | NM_015394 | zinc finger protein 10 |
| ST6GAL2 | NM_001142351 | ST6 beta-galactosamide alpha-2,6-sialyltranferase 2 |
| TMEM184B | NM_001195071 | transmembrane protein 184B |
| MLL3 | NM_170606 | myeloid/lymphoid or mixed-lineage leukemia 3 |
| LEPR | NM_001003679 | leptin receptor |
| ADCYAP1 | NM_001099733 | adenylate cyclase activating polypeptide 1 (pituitary) |
| MEOX2 | NM_005924 | mesenchyme homeobox 2 |
| ZNF302 | NM_001012320 | zinc finger protein 302 |
| PPM1B | NM_177968 | protein phosphatase, Mg2+/Mn2+ dependent, 1B |
| ZNF197 | NM_001024855 | zinc finger protein 197 |
| NRF1 | NM_001040110 | nuclear respiratory factor 1 |
| DDX26B | NM_182540 | DEAD/H (Asp-Glu-Ala-Asp/His) box polypeptide 26B |
| ULBP1 | NM_025218 | UL16 binding protein 1 |
| CASK | NM_001126054 | calcium/calmodulin-dependent serine protein kinase (MAGUK family) |
| GALC | NM_000153 | galactosylceramidase |
| GOLGA8A | NM_181077 | golgin A8 family, member A |
| RUFY2 | NM_017987 | RUN and FYVE domain containing 2 |
| FAM76A | NM_001143912 | family with sequence similarity 76, member A |
| KCNQ5 | NM_001160130 | potassium voltage-gated channel, KQT-like subfamily, member 5 |
| GPD1L | NM_015141 | glycerol-3-phosphate dehydrogenase 1-like |
| DNAJC24 | NM_181706 | DnaJ (Hsp40) homolog, subfamily C, member 24 |
| SAP18 | NM_005870 | Sin3A-associated protein, 18kDa |
| ELAVL2 | NM_001171195 | ELAV (embryonic lethal, abnormal vision, Drosophila)-like 2 (Hu antigen B) |
| CCNJ | NM_001134375 | cyclin J |

| | | |
|---|---|---|
| GPC1 | NM_002081 | glypican 1 |
| PPP3R1 | NM_000945 | protein phosphatase 3, regulatory subunit B, alpha |
| GRSF1 | NM_001098477 | G-rich RNA sequence binding factor 1 |
| MITD1 | NM_138798 | MIT, microtubule interacting and transport, domain containing 1 |
| FAM26E | NM_153711 | family with sequence similarity 26, member E |
| PLCL2 | NM_001144382 | phospholipase C-like 2 |
| PUM2 | NM_015317 | pumilio homolog 2 (Drosophila) |
| SVIP | NM_148893 | small VCP/p97-interacting protein |
| PHC3 | NM_024947 | polyhomeotic homolog 3 (Drosophila) |
| CAMSAP1 | NM_015447 | calmodulin regulated spectrin-associated protein 1 |
| CHTOP | NM_001206612 | chromatin target of PRMT1 |
| ASTN1 | NM_004319 | astrotactin 1 |
| CEACAM1 | NM_001024912 | carcinoembryonic antigen-related cell adhesion molecule 1 (biliary glycoprotein) |
| C5orf41 | NM_153607 | chromosome 5 open reading frame 41 |
| UBA6 | NM_018227 | ubiquitin-like modifier activating enzyme 6 |
| IFRD1 | NM_001007245 | interferon-related developmental regulator 1 |
| TMSB10 | NM_021103 | thymosin beta 10 |
| PLA2G4A | NM_024420 | phospholipase A2, group IVA (cytosolic, calcium-dependent) |
| ZNF568 | NM_001204835 | zinc finger protein 568 |
| LRRC2 | NM_024512 | leucine rich repeat containing 2 |
| MAB21L1 | NM_005584 | mab-21-like 1 (C. elegans) |
| FAM126A | NM_032581 | family with sequence similarity 126, member A |
| USP49 | NM_018561 | ubiquitin specific peptidase 49 |
| SMG1 | NM_015092 | smg-1 homolog, phosphatidylinositol 3-kinase-related kinase (C. elegans) |
| CDH13 | NM_001220488 | cadherin 13, H-cadherin (heart) |
| SMPX | NM_014332 | small muscle protein, X-linked |
| C2orf89 | NM_001080824 | chromosome 2 open reading frame 89 |
| NAALAD2 | NM_005467 | N-acetylated alpha-linked acidic dipeptidase 2 |
| SLFN5 | NM_144975 | schlafen family member 5 |
| CLEC1B | NM_001099431 | C-type lectin domain family 1, member B |
| FGF2 | NM_002006 | fibroblast growth factor 2 (basic) |
| AUTS2 | NM_001127231 | autism susceptibility candidate 2 |
| PDZD2 | NM_178140 | PDZ domain containing 2 |
| KIAA1239 | NM_001144990 | KIAA1239 |
| LRRC55 | NM_001005210 | leucine rich repeat containing 55 |
| ZNHIT6 | NM_001170670 | zinc finger, HIT-type containing 6 |
| RGPD5 | NM_005054 | RANBP2-like and GRIP domain containing 5 |
| GTF3C3 | NM_001206774 | general transcription factor IIIC, polypeptide 3, 102kDa |
| ITM2A | NM_001171581 | integral membrane protein 2A |

| | | |
|---|---|---|
| CTBP1 | NM_001012614 | C-terminal binding protein 1 |
| MPHOSPH8 | NM_017520 | M-phase phosphoprotein 8 |
| MUC15 | NM_001135091 | mucin 15, cell surface associated |
| CDH19 | NM_021153 | cadherin 19, type 2 |
| AKAP1 | NM_001242902 | A kinase (PRKA) anchor protein 1 |
| ZNF441 | NM_152355 | zinc finger protein 441 |
| RGPD6 | NM_001123363 | RANBP2-like and GRIP domain containing 6 |
| ZNF813 | NM_001004301 | zinc finger protein 813 |
| RGPD8 | NM_001164463 | RANBP2-like and GRIP domain containing 8 |
| GLRX3 | NM_006541 | glutaredoxin 3 |
| WFDC6 | NM_080827 | WAP four-disulfide core domain 6 |
| ALDH18A1 | NM_001017423 | aldehyde dehydrogenase 18 family, member A1 |
| C15orf29 | NM_024713 | chromosome 15 open reading frame 29 |
| MTM1 | NM_000252 | myotubularin 1 |
| FAM13A | NM_001015045 | family with sequence similarity 13, member A |
| TANC2 | NM_025185 | tetratricopeptide repeat, ankyrin repeat and coiled-coil containing 2 |
| SLC36A3 | NM_001145017 | solute carrier family 36 (proton/amino acid symporter), member 3 |
| LCORL | NM_001166139 | ligand dependent nuclear receptor corepressor-like |
| DENND1B | NM_001195215 | DENN/MADD domain containing 1B |
| N6AMT1 | NM_013240 | N-6 adenine-specific DNA methyltransferase 1 (putative) |
| CSTF3 | NM_001326 | cleavage stimulation factor, 3' pre-RNA, subunit 3, 77kDa |
| NRG1 | NM_001159995 | neuregulin 1 |
| TMEM30A | NM_001143958 | transmembrane protein 30A |
| SPRED2 | NM_001128210 | sprouty-related, EVH1 domain containing 2 |
| CALCRL | NM_005795 | calcitonin receptor-like |
| WIF1 | NM_007191 | WNT inhibitory factor 1 |
| ASCL4 | NM_203436 | achaete-scute complex homolog 4 (Drosophila) |
| TCTEX1D1 | NM_152665 | Tctex1 domain containing 1 |
| MAT1A | NM_000429 | methionine adenosyltransferase I, alpha |
| CTPS2 | NM_001144002 | CTP synthase II |
| GPR64 | NM_001079858 | G protein-coupled receptor 64 |
| ZMAT1 | NM_001011657 | zinc finger, matrin-type 1 |
| IPMK | NM_152230 | inositol polyphosphate multikinase |
| EIF3B | NM_001037283 | eukaryotic translation initiation factor 3, subunit B |
| AK3 | NM_001199852 | adenylate kinase 3 |
| RBM28 | NM_001166135 | RNA binding motif protein 28 |
| GRPR | NM_005314 | gastrin-releasing peptide receptor |
| CEP170 | NM_001042404 | centrosomal protein 170kDa |
| INO80D | NM_017759 | INO80 complex subunit D |

| | | |
|---|---|---|
| TTL | NM_153712 | tubulin tyrosine ligase |
| PPARGC1A | NM_013261 | peroxisome proliferator-activated receptor gamma, coactivator 1 alpha |
| PISD | NM_014338 | phosphatidylserine decarboxylase |
| MIDN | NM_177401 | midnolin |
| FOXP2 | NM_001172766 | forkhead box P2 |
| USP37 | NM_020935 | ubiquitin specific peptidase 37 |
| ATP1B1 | NM_001677 | ATPase, Na+/K+ transporting, beta 1 polypeptide |
| STEAP2 | NM_001040665 | six transmembrane epithelial antigen of the prostate 2 |
| MCM8 | NM_032485 | minichromosome maintenance complex component 8 |
| MZT1 | NM_001071775 | mitotic spindle organizing protein 1 |
| RALGAPB | NM_020336 | Ral GTPase activating protein, beta subunit (non-catalytic) |
| MAP2K1 | NM_002755 | mitogen-activated protein kinase kinase 1 |
| C18orf10 | NM_015476 | chromosome 18 open reading frame 10 |
| KIF21A | NM_001173463 | kinesin family member 21A |
| PEX5 | NM_000319 | peroxisomal biogenesis factor 5 |
| ZNF100 | NM_173531 | zinc finger protein 100 |
| MRO | NM_001127174 | maestro |
| TAF4 | NM_003185 | TAF4 RNA polymerase II, TATA box binding protein (TBP)-associated factor, 135kDa |
| TMEM108 | NM_001136469 | transmembrane protein 108 |
| NRAS | NM_002524 | neuroblastoma RAS viral (v-ras) oncogene homolog |
| CD46 | NM_002389 | CD46 molecule, complement regulatory protein |
| FOXE1 | NM_004473 | forkhead box E1 (thyroid transcription factor 2) |
| HSPA6 | NM_002155 | heat shock 70kDa protein 6 (HSP70B') |
| PDHX | NM_001135024 | pyruvate dehydrogenase complex, component X |
| BBS9 | NM_001033604 | Bardet-Biedl syndrome 9 |
| CACNA1B | NM_000718 | calcium channel, voltage-dependent, N type, alpha 1B subunit |
| MTTP | NM_000253 | microsomal triglyceride transfer protein |
| LSM11 | NM_173491 | LSM11, U7 small nuclear RNA associated |
| SYPL1 | NM_006754 | synaptophysin-like 1 |
| ZNF30 | NM_001099437 | zinc finger protein 30 |
| FMO3 | NM_001002294 | flavin containing monooxygenase 3 |
| PCDH7 | NM_001173523 | protocadherin 7 |
| PRKCA | NM_002737 | protein kinase C, alpha |
| KBTBD6 | NM_152903 | kelch repeat and BTB (POZ) domain containing 6 |
| ZNF776 | NM_173632 | zinc finger protein 776 |
| SEPT2 | NM_001008491 | septin 2 |
| MATN3 | NM_002381 | matrilin 3 |
| WDR7 | NM_015285 | WD repeat domain 7 |
| RPL23A | NM_000984 | ribosomal protein L23a |

| | | |
|---|---|---|
| ZNF557 | NM_001044387 | zinc finger protein 557 |
| BNIP2 | NM_004330 | BCL2/adenovirus E1B 19kDa interacting protein 2 |
| ZC3H13 | NM_015070 | zinc finger CCCH-type containing 13 |
| UBR3 | NM_172070 | ubiquitin protein ligase E3 component n-recognin 3 (putative) |
| ZNF274 | NM_016324 | zinc finger protein 274 |
| ZNF233 | NM_001207005 | zinc finger protein 233 |
| OXGR1 | NM_080818 | oxoglutarate (alpha-ketoglutarate) receptor 1 |
| GRM6 | NM_000843 | glutamate receptor, metabotropic 6 |
| GNA14 | NM_004297 | guanine nucleotide binding protein (G protein), alpha 14 |
| SLC6A15 | NM_001146335 | solute carrier family 6 (neutral amino acid transporter), member 15 |
| TBX15 | NM_152380 | T-box 15 |
| RAB38 | NM_022337 | RAB38, member RAS oncogene family |
| ZMYM1 | NM_024772 | zinc finger, MYM-type 1 |
| MCM6 | NM_005915 | minichromosome maintenance complex component 6 |
| MYO1E | NM_004998 | myosin IE |
| RBAK | NM_001204456 | RB-associated KRAB zinc finger |
| CYR61 | NM_001554 | cysteine-rich, angiogenic inducer, 61 |
| FAM108B1 | NM_001025780 | family with sequence similarity 108, member B1 |
| TNPO1 | NM_002270 | transportin 1 |
| C14orf169 | NM_024644 | chromosome 14 open reading frame 169 |
| PGRMC1 | NM_006667 | progesterone receptor membrane component 1 |
| C8orf41 | NM_001102401 | chromosome 8 open reading frame 41 |
| ZNF828 | NM_001164144 | zinc finger protein 828 |
| EFCAB5 | NM_001145053 | EF-hand calcium binding domain 5 |
| TPH2 | NM_173353 | tryptophan hydroxylase 2 |
| UBE2Q1 | NM_017582 | ubiquitin-conjugating enzyme E2Q family member 1 |
| PPP4R4 | NM_058237 | protein phosphatase 4, regulatory subunit 4 |
| PROX1 | NM_002763 | prospero homeobox 1 |
| TTC39A | NM_001080494 | tetratricopeptide repeat domain 39A |
| OTUD4 | NM_001102653 | OTU domain containing 4 |
| PEAK1 | NM_024776 | NKF3 kinase family member |
| TMEM50B | NM_006134 | transmembrane protein 50B |
| RAP2A | NM_021033 | RAP2A, member of RAS oncogene family |
| FGF1 | NM_000800 | fibroblast growth factor 1 (acidic) |
| ACN9 | NM_020186 | ACN9 homolog (S. cerevisiae) |
| RHOQ | NM_012249 | ras homolog gene family, member Q |
| SLC8A1 | NM_001112800 | solute carrier family 8 (sodium/calcium exchanger), member 1 |
| NPHP3 | NM_153240 | nephronophthisis 3 (adolescent) |
| LGR4 | NM_018490 | leucine-rich repeat containing G protein-coupled receptor 4 |

| | | |
|---|---|---|
| PRKAR2A | NM_004157 | protein kinase, cAMP-dependent, regulatory, type II, alpha |
| CAP2 | NM_006366 | CAP, adenylate cyclase-associated protein, 2 (yeast) |
| ODZ2 | NM_001122679 | odz, odd Oz/ten-m homolog 2 (Drosophila) |
| OSTC | NM_021227 | oligosaccharyltransferase complex subunit |
| PIGK | NM_005482 | phosphatidylinositol glycan anchor biosynthesis, class K |
| CETN3 | NM_004365 | centrin, EF-hand protein, 3 |
| TRMT5 | NM_020810 | TRM5 tRNA methyltransferase 5 homolog (S. cerevisiae) |
| IL6ST | NM_001190981 | interleukin 6 signal transducer (gp130, oncostatin M receptor) |
| PTPN2 | NM_001207013 | protein tyrosine phosphatase, non-receptor type 2 |
| TRIM24 | NM_003852 | tripartite motif containing 24 |
| SLC9A8 | NM_015266 | solute carrier family 9 (sodium/hydrogen exchanger), member 8 |
| HMBOX1 | NM_001135726 | homeobox containing 1 |
| LY75 | NM_002349 | lymphocyte antigen 75 |
| PRPH2 | NM_000322 | peripherin 2 (retinal degeneration, slow) |
| HPS3 | NM_032383 | Hermansky-Pudlak syndrome 3 |
| LIMD1 | NM_014240 | LIM domains containing 1 |
| HNRNPH1 | NM_005520 | heterogeneous nuclear ribonucleoprotein H1 (H) |
| CD36 | NM_001001548 | CD36 molecule (thrombospondin receptor) |
| HEPACAM2 | NM_001039372 | HEPACAM family member 2 |
| TTPAL | NM_001039199 | tocopherol (alpha) transfer protein-like |
| ZNF266 | NM_006631 | zinc finger protein 266 |
| STYX | NM_001130701 | serine/threonine/tyrosine interacting protein |
| ATP1B4 | NM_001142447 | ATPase, Na+/K+ transporting, beta 4 polypeptide |
| DAGLA | NM_006133 | diacylglycerol lipase, alpha |
| ZNF626 | NM_145297 | zinc finger protein 626 |
| PIK3C2A | NM_002645 | phosphoinositide-3-kinase, class 2, alpha polypeptide |
| ZNF322B | NM_199005 | zinc finger protein 322B |
| PTPRD | NM_001040712 | protein tyrosine phosphatase, receptor type, D |
| DMXL2 | NM_001174116 | Dmx-like 2 |
| TMEM56 | NM_001199679 | transmembrane protein 56 |
| NFYB | NM_006166 | nuclear transcription factor Y, beta |
| MYCT1 | NM_025107 | myc target 1 |
| NLRP8 | NM_176811 | NLR family, pyrin domain containing 8 |
| PPM1K | NM_152542 | protein phosphatase, Mg2+/Mn2+ dependent, 1K |
| ZNF506 | NM_001099269 | zinc finger protein 506 |
| PDE4DIP | NM_001002811 | phosphodiesterase 4D interacting protein |
| MYO1B | NM_001130158 | myosin IB |
| SCML2 | NM_006089 | sex comb on midleg-like 2 (Drosophila) |
| CANX | NM_001024649 | calnexin |

| | | |
|---|---|---|
| PSG1 | NM_001184825 | pregnancy specific beta-1-glycoprotein 1 |
| MELK | NM_014791 | maternal embryonic leucine zipper kinase |
| GIN1 | NM_017676 | gypsy retrotransposon integrase 1 |
| BICC1 | NM_001080512 | bicaudal C homolog 1 (Drosophila) |
| VPS36 | NM_016075 | vacuolar protein sorting 36 homolog (S. cerevisiae) |
| MFN1 | NM_033540 | mitofusin 1 |
| TRIM8 | NM_030912 | tripartite motif containing 8 |
| DBX2 | NM_001004329 | developing brain homeobox 2 |
| ARID1A | NM_006015 | AT rich interactive domain 1A (SWI-like) |
| BAIAP2 | NM_017450 | BAI1-associated protein 2 |
| WDR33 | NM_018383 | WD repeat domain 33 |
| LHX1 | NM_005568 | LIM homeobox 1 |
| MAPKBP1 | NM_001128608 | mitogen-activated protein kinase binding protein 1 |
| ZNF257 | NM_033468 | zinc finger protein 257 |
| CHGA | NM_001275 | chromogranin A (parathyroid secretory protein 1) |
| HNRNPH2 | NM_001032393 | protein-coding |
| RCBTB1 | NM_018191 | regulator of chromosome condensation (RCC1) and BTB (POZ) domain containing |
| MAGEE1 | NM_020932 | melanoma antigen family E, 1 |
| RPL36A-HNRNP | NM_001199973 | RPL36A-HNRNPH2 readthrough |
| IPO8 | NM_001190995 | importin 8 |
| RCN2 | NM_002902 | reticulocalbin 2, EF-hand calcium binding domain |
| SRSF2 | NM_001195427 | serine/arginine-rich splicing factor 2 |
| CORIN | NM_006587 | corin, serine peptidase |
| VMA21 | NM_001017980 | VMA21 vacuolar H+-ATPase homolog (S. cerevisiae) |
| C6orf106 | NM_022758 | chromosome 6 open reading frame 106 |
| CEP44 | NM_001040157 | centrosomal protein 44kDa |
| CEP72 | NM_018140 | centrosomal protein 72kDa |
| TXLNG | NM_001168683 | taxilin gamma |
| GRIK3 | NM_000831 | glutamate receptor, ionotropic, kainate 3 |
| MTMR9 | NM_015458 | myotubularin related protein 9 |
| PRSS35 | NM_001170423 | protease, serine, 35 |
| JAG1 | NM_000214 | jagged 1 |
| HMMR | NM_001142556 | hyaluronan-mediated motility receptor (RHAMM) |
| NXPH1 | NM_152745 | neurexophilin 1 |
| S100PBP | NM_022753 | S100P binding protein |
| HS6ST3 | NM_153456 | heparan sulfate 6-O-sulfotransferase 3 |
| C10orf88 | NM_024942 | chromosome 10 open reading frame 88 |
| CDH15 | NM_004933 | cadherin 15, type 1, M-cadherin (myotubule) |
| IL1RN | NM_000577 | interleukin 1 receptor antagonist |

| | | |
|---|---|---|
| INPP1 | NM_001128928 | inositol polyphosphate-1-phosphatase |
| SNAI2 | NM_003068 | snail homolog 2 (Drosophila) |
| CADM4 | NM_145296 | cell adhesion molecule 4 |
| PIK3C2B | NM_002646 | phosphoinositide-3-kinase, class 2, beta polypeptide |
| GBE1 | NM_000158 | glucan (1,4-alpha-), branching enzyme 1 |
| MBIP | NM_001144891 | MAP3K12 binding inhibitory protein 1 |
| ZNF181 | NM_001029997 | zinc finger protein 181 |
| RBM46 | NM_144979 | RNA binding motif protein 46 |
| C11orf45 | NM_145013 | chromosome 11 open reading frame 45 |
| ARHGAP6 | NM_006125 | Rho GTPase activating protein 6 |
| MTRF1 | NM_004294 | mitochondrial translational release factor 1 |
| GDAP2 | NM_001135589 | ganglioside induced differentiation associated protein 2 |
| RNF146 | NM_001242844 | ring finger protein 146 |
| CPNE8 | NM_153634 | copine VIII |
| YOD1 | NM_018566 | YOD1 OTU deubiquinating enzyme 1 homolog (S. cerevisiae) |
| YLPM1 | NM_019589 | YLP motif containing 1 |
| ODZ1 | NM_001163278 | odz, odd Oz/ten-m homolog 1 (Drosophila) |
| CACNA2D4 | NM_172364 | calcium channel, voltage-dependent, alpha 2/delta subunit 4 |
| C22orf39 | NM_173793 | chromosome 22 open reading frame 39 |
| RBBP6 | NM_032626 | retinoblastoma binding protein 6 |
| GNAI1 | NM_002069 | guanine nucleotide binding protein (G protein), alpha inhibiting activity polypeptide 1 |
| TNFSF15 | NM_001204344 | tumor necrosis factor (ligand) superfamily, member 15 |
| ASXL1 | NM_001164603 | additional sex combs like 1 (Drosophila) |
| PTPLAD2 | NM_001010915 | protein tyrosine phosphatase-like A domain containing 2 |
| CWF19L2 | NM_152434 | CWF19-like 2, cell cycle control (S. pombe) |
| CNR1 | NM_001160226 | cannabinoid receptor 1 (brain) |
| CHRDL1 | NM_001143981 | chordin-like 1 |
| ALG14 | NM_144988 | asparagine-linked glycosylation 14 homolog (S. cerevisiae) |
| SLC30A8 | NM_001172811 | solute carrier family 30 (zinc transporter), member 8 |
| DNM1L | NM_005690 | dynamin 1-like |
| YAF2 | NM_001190977 | YY1 associated factor 2 |
| CDK16 | NM_001170460 | cyclin-dependent kinase 16 |
| IQGAP1 | NM_003870 | IQ motif containing GTPase activating protein 1 |
| TMED10 | NM_006827 | transmembrane emp24-like trafficking protein 10 (yeast) |
| HOOK3 | NM_032410 | hook homolog 3 (Drosophila) |
| SECISBP2L | NM_001193489 | SECIS binding protein 2-like |
| RUFY3 | NM_001037442 | RUN and FYVE domain containing 3 |
| N4BP2 | NM_018177 | NEDD4 binding protein 2 |
| RPL34 | NM_000995 | ribosomal protein L34 |

| | | |
|---|---|---|
| BCOR | NM_001123383 | BCL6 corepressor |
| PCGF3 | NM_006315 | polycomb group ring finger 3 |
| RRP15 | NM_016052 | ribosomal RNA processing 15 homolog (S. cerevisiae) |
| DYRK1A | NM_001396 | dual-specificity tyrosine-(Y)-phosphorylation regulated kinase 1A |
| DDX21 | NM_004728 | DEAD (Asp-Glu-Ala-Asp) box polypeptide 21 |
| C11orf87 | NM_207645 | chromosome 11 open reading frame 87 |
| ENY2 | NM_001193557 | enhancer of yellow 2 homolog (Drosophila) |
| MAFB | NM_005461 | v-maf musculoaponeurotic fibrosarcoma oncogene homolog B (avian) |
| CDH8 | NM_001796 | cadherin 8, type 2 |
| ZNF264 | NM_003417 | zinc finger protein 264 |
| FBLN1 | NM_006486 | fibulin 1 |
| GOLGA8B | NM_001023567 | golgin A8 family, member B |
| TRIM10 | NM_006778 | tripartite motif containing 10 |
| SCML1 | NM_001037535 | sex comb on midleg-like 1 (Drosophila) |
| MYOM1 | NM_003803 | myomesin 1, 185kDa |
| SCAI | NM_001144877 | suppressor of cancer cell invasion |
| VAPB | NM_001195677 | VAMP (vesicle-associated membrane protein)-associated protein B and C |
| C17orf102 | NM_207454 | chromosome 17 open reading frame 102 |
| ANO6 | NM_001025356 | anoctamin 6 |
| CDYL | NM_001143970 | chromodomain protein, Y-like |
| RABEP1 | NM_001083585 | rabaptin, RAB GTPase binding effector protein 1 |
| C9orf82 | NM_001167575 | chromosome 9 open reading frame 82 |
| RNF38 | NM_022781 | ring finger protein 38 |
| NTM | NM_001048209 | neurotrimin |
| ZNF397 | NM_001135178 | zinc finger protein 397 |
| SEMA4A | NM_001193300 | sema domain, immunoglobulin domain (Ig), transmembrane domain (TM) and short cytoplasmic domain, (semaphorin) 4A |
| PLRG1 | NM_001201564 | pleiotropic regulator 1 |
| ITM2B | NM_021999 | integral membrane protein 2B |
| KIF21B | NM_017596 | kinesin family member 21B |
| MCM10 | NM_018518 | minichromosome maintenance complex component 10 |
| POLR2D | NM_004805 | polymerase (RNA) II (DNA directed) polypeptide D |
| GSK3B | NM_001146156 | glycogen synthase kinase 3 beta |
| PIGB | NM_004855 | phosphatidylinositol glycan anchor biosynthesis, class B |
| AQR | NM_014691 | aquarius homolog (mouse) |
| TMEM19 | NM_018279 | transmembrane protein 19 |
| PI4K2A | NM_018425 | phosphatidylinositol 4-kinase type 2 alpha |
| C9orf123 | NM_033428 | chromosome 9 open reading frame 123 |
| PDIA6 | NM_005742 | protein disulfide isomerase family A, member 6 |

| | | |
|---|---|---|
| C7orf58 | NM_024913 | chromosome 7 open reading frame 58 |
| TANC1 | NM_001145909 | tetratricopeptide repeat, ankyrin repeat and coiled-coil containing 1 |
| LRRK2 | NM_198578 | leucine-rich repeat kinase 2 |
| TMEM131 | NM_015348 | transmembrane protein 131 |
| SRSF7 | NM_001031684 | serine/arginine-rich splicing factor 7 |
| ACTB | NM_001101 | actin, beta |
| OR10W1 | NM_207374 | olfactory receptor, family 10, subfamily W, member 1 |
| IDS | NM_000202 | iduronate 2-sulfatase |
| COX19 | NM_001031617 | COX19 cytochrome c oxidase assembly homolog (S. cerevisiae) |
| RGPD4 | NM_182588 | RANBP2-like and GRIP domain containing 4 |
| ANKRD18A | NM_147195 | ankyrin repeat domain 18A |
| PLSCR1 | NM_021105 | phospholipid scramblase 1 |
| ZNF780B | NM_001005851 | zinc finger protein 780B |
| STX2 | NM_001980 | syntaxin 2 |
| WNT11 | NM_004626 | wingless-type MMTV integration site family, member 11 |
| DSCR6 | NM_018962 | Down syndrome critical region gene 6 |
| NID1 | NM_002508 | nidogen 1 |
| SCN9A | NM_002977 | sodium channel, voltage-gated, type IX, alpha subunit |
| P2RY1 | NM_002563 | purinergic receptor P2Y, G-protein coupled, 1 |
| SUPT7L | NM_014860 | suppressor of Ty 7 (S. cerevisiae)-like |
| RCBTB2 | NM_001268 | regulator of chromosome condensation (RCC1) and BTB (POZ) domain containing |
| SLC35A5 | NM_017945 | solute carrier family 35, member A5 |
| STK35 | NM_080836 | serine/threonine kinase 35 |
| HCFC2 | NM_013320 | host cell factor C2 |
| RYR2 | NM_001035 | ryanodine receptor 2 (cardiac) |
| WDR17 | NM_170710 | WD repeat domain 17 |
| C6orf201 | NM_001085401 | chromosome 6 open reading frame 201 |
| AGTR2 | NM_000686 | angiotensin II receptor, type 2 |
| FCRL4 | NM_031282 | Fc receptor-like 4 |
| SLC24A3 | NM_020689 | solute carrier family 24 (sodium/potassium/calcium exchanger), member 3 |
| CSGALNACT2 | NM_018590 | chondroitin sulfate N-acetylgalactosaminyltransferase 2 |
| POLR3GL | NM_032305 | polymerase (RNA) III (DNA directed) polypeptide G (32kD)-like |
| KIF23 | NM_004856 | kinesin family member 23 |
| HEXIM1 | NM_006460 | hexamethylene bis-acetamide inducible 1 |
| ZNF250 | NM_001109689 | zinc finger protein 250 |
| UPRT | NM_145052 | uracil phosphoribosyltransferase (FUR1) homolog (S. cerevisiae) |
| FAM133B | NM_001040057 | family with sequence similarity 133, member B |
| DLG2 | NM_001142699 | discs, large homolog 2 (Drosophila) |
| C11orf21 | NM_001142946 | chromosome 11 open reading frame 21 |

| | | |
|---|---|---|
| PCDH18 | NM_019035 | protocadherin 18 |
| CKAP5 | NM_001008938 | cytoskeleton associated protein 5 |
| SHISA2 | NM_001007538 | shisa homolog 2 (Xenopus laevis) |
| RPL22 | NM_000983 | ribosomal protein L22 |
| TMEM67 | NM_001142301 | transmembrane protein 67 |
| GRID1 | NM_017551 | glutamate receptor, ionotropic, delta 1 |
| RAB40B | NM_006822 | RAB40B, member RAS oncogene family |
| HNRNPA3 | NM_194247 | heterogeneous nuclear ribonucleoprotein A3 |
| DCUN1D5 | NM_032299 | DCN1, defective in cullin neddylation 1, domain containing 5 (S. cerevisiae) |
| MBP | NM_001025100 | myelin basic protein |
| NSD1 | NM_022455 | nuclear receptor binding SET domain protein 1 |
| RIT1 | NM_006912 | Ras-like without CAAX 1 |
| TEX12 | NM_031275 | testis expressed 12 |
| SGIP1 | NM_032291 | SH3-domain GRB2-like (endophilin) interacting protein 1 |
| SYNE2 | NM_015180 | spectrin repeat containing, nuclear envelope 2 |
| C17orf56 | NM_144679 | chromosome 17 open reading frame 56 |
| SLC40A1 | NM_014585 | solute carrier family 40 (iron-regulated transporter), member 1 |
| COL4A3 | NM_000091 | collagen, type IV, alpha 3 (Goodpasture antigen) |
| FBXL7 | NM_012304 | F-box and leucine-rich repeat protein 7 |
| KBTBD8 | NM_032505 | kelch repeat and BTB (POZ) domain containing 8 |
| PON3 | NM_000940 | paraoxonase 3 |
| SMAD4 | NM_005359 | SMAD family member 4 |
| PNN | NM_002687 | pinin, desmosome associated protein |
| PER3 | NM_016831 | period homolog 3 (Drosophila) |
| SUN1 | NM_001130965 | Sad1 and UNC84 domain containing 1 |
| TOR1AIP2 | NM_022347 | torsin A interacting protein 2 |
| NCKAP5 | NM_207363 | NCK-associated protein 5 |
| DNAJC27 | NM_001198559 | DnaJ (Hsp40) homolog, subfamily C, member 27 |
| RAI2 | NM_001172732 | retinoic acid induced 2 |
| MAT2B | NM_013283 | methionine adenosyltransferase II, beta |
| RAD51D | NM_001142571 | RAD51 homolog D (S. cerevisiae) |
| MAP1LC3B | NM_022818 | microtubule-associated protein 1 light chain 3 beta |
| MAGT1 | NM_032121 | magnesium transporter 1 |
| RGS21 | NM_001039152 | regulator of G-protein signaling 21 |
| ESAM | NM_138961 | endothelial cell adhesion molecule |
| CNKSR3 | NM_173515 | CNKSR family member 3 |
| TBC1D13 | NM_018201 | TBC1 domain family, member 13 |
| ANKRD46 | NM_198401 | ankyrin repeat domain 46 |
| UBE2E3 | NM_006357 | ubiquitin-conjugating enzyme E2E 3 |

| | | |
|---|---|---|
| SYTL4 | NM_001129896 | synaptotagmin-like 4 |
| SIRT1 | NM_001142498 | sirtuin 1 |
| BTG2 | NM_006763 | BTG family, member 2 |
| RC3H1 | NM_172071 | ring finger and CCCH-type domains 1 |
| TXNDC5 | NM_001145549 | thioredoxin domain containing 5 (endoplasmic reticulum) |
| AFAP1 | NM_001134647 | actin filament associated protein 1 |
| PCDHB2 | NM_018936 | protocadherin beta 2 |
| ZNF273 | NM_021148 | zinc finger protein 273 |
| GLYAT | NM_201648 | glycine-N-acyltransferase |
| TSHZ3 | NM_020856 | teashirt zinc finger homeobox 3 |
| CUL3 | NM_003590 | cullin 3 |
| CKAP2 | NM_001098525 | cytoskeleton associated protein 2 |
| CXorf26 | NM_016500 | chromosome X open reading frame 26 |
| GALNT1 | NM_020474 | UDP-N-acetyl-alpha-D-galactosamine:polypeptide N-acetylgalactosaminyltransferase |
| RAP1B | NM_001010942 | RAP1B, member of RAS oncogene family |
| FAM65B | NM_014722 | family with sequence similarity 65, member B |
| C4orf49 | NM_032623 | chromosome 4 open reading frame 49 |
| TMEM178 | NM_001167959 | transmembrane protein 178 |
| THBS1 | NM_003246 | thrombospondin 1 |
| IFIT1 | NM_001548 | interferon-induced protein with tetratricopeptide repeats 1 |
| AP3D1 | NM_001077523 | adaptor-related protein complex 3, delta 1 subunit |
| SGK3 | NM_001033578 | serum/glucocorticoid regulated kinase family, member 3 |
| PAPD4 | NM_001114393 | PAP associated domain containing 4 |
| C8orf44-SGK3 | NM_001204173 | C8orf44-SGK3 readthrough |
| MRPL19 | NM_014763 | mitochondrial ribosomal protein L19 |
| ZCCHC6 | NM_001185059 | zinc finger, CCHC domain containing 6 |
| NUDCD1 | NM_001128211 | NudC domain containing 1 |
| XG | NM_001141919 | Xg blood group |
| PPIP5K1 | NM_001130858 | diphosphoinositol pentakisphosphate kinase 1 |
| C15orf63 | NM_001199885 | chromosome 15 open reading frame 63 |
| DIRC2 | NM_032839 | disrupted in renal carcinoma 2 |
| ZNF518A | NM_014803 | zinc finger protein 518A |
| SLC5A3 | NM_006933 | solute carrier family 5 (sodium/myo-inositol cotransporter), member 3 |
| NEK4 | NM_001193533 | NIMA (never in mitosis gene a)-related kinase 4 |
| SPTLC1 | NM_178324 | serine palmitoyltransferase, long chain base subunit 1 |
| AKIRIN2 | NM_018064 | akirin 2 |
| PDCD6IP | NM_001162429 | programmed cell death 6 interacting protein |
| MS4A3 | NM_001031666 | membrane-spanning 4-domains, subfamily A, member 3 (hematopoietic cell-specific) |
| FBN2 | NM_001999 | fibrillin 2 |

| | | |
|---|---|---|
| MGEA5 | NM_001142434 | meningioma expressed antigen 5 (hyaluronidase) |
| IBTK | NM_015525 | inhibitor of Bruton agammaglobulinemia tyrosine kinase |
| C6orf115 | NM_021243 | chromosome 6 open reading frame 115 |
| FOXN3 | NM_001085471 | forkhead box N3 |
| C5orf47 | NM_001144954 | chromosome 5 open reading frame 47 |
| NANP | NM_152667 | N-acetylneuraminic acid phosphatase |
| MCU | NM_138357 | mitochondrial calcium uniporter |
| DDX39A | NM_005804 | DEAD (Asp-Glu-Ala-Asp) box polypeptide 39A |
| NUDT19 | NM_001105570 | nudix (nucleoside diphosphate linked moiety X)-type motif 19 |
| CAPN7 | NM_014296 | calpain 7 |
| SIP1 | NM_001009182 | survival of motor neuron protein interacting protein 1 |
| GCA | NM_012198 | grancalcin, EF-hand calcium binding protein |
| CGGBP1 | NM_001008390 | CGG triplet repeat binding protein 1 |
| C2orf43 | NM_021925 | chromosome 2 open reading frame 43 |
| CLEC4M | NM_001144904 | C-type lectin domain family 4, member M |
| ZNF295 | NM_001098402 | zinc finger protein 295 |
| REPS2 | NM_001080975 | RALBP1 associated Eps domain containing 2 |
| C20orf11 | NM_017896 | chromosome 20 open reading frame 11 |
| RLN1 | NM_006911 | relaxin 1 |
| DCLK1 | NM_001195415 | doublecortin-like kinase 1 |
| FGF12 | NM_004113 | fibroblast growth factor 12 |
| BRCC3 | NM_001018055 | BRCA1/BRCA2-containing complex, subunit 3 |
| USP27X | NM_001145073 | ubiquitin specific peptidase 27, X-linked |
| RAB31 | NM_006868 | RAB31, member RAS oncogene family |
| HACE1 | NM_020771 | HECT domain and ankyrin repeat containing, E3 ubiquitin protein ligase 1 |
| YPEL4 | NM_145008 | yippee-like 4 (Drosophila) |
| EHF | NM_001206615 | ets homologous factor |
| FCAMR | NM_001122979 | Fc receptor, IgA, IgM, high affinity |
| C5orf64 | NM_173667 | chromosome 5 open reading frame 64 |
| NRXN1 | NM_001135659 | neurexin 1 |
| SFMBT1 | NM_001005158 | Scm-like with four mbt domains 1 |
| BRWD1 | NM_001007246 | bromodomain and WD repeat domain containing 1 |
| SLC16A9 | NM_194298 | solute carrier family 16, member 9 (monocarboxylic acid transporter 9) |
| PLA2G2F | NM_022819 | phospholipase A2, group IIF |
| ZC3H7A | NM_014153 | zinc finger CCCH-type containing 7A |
| DIAPH1 | NM_001079812 | diaphanous homolog 1 (Drosophila) |
| ZNF17 | NM_006959 | zinc finger protein 17 |
| ASAP2 | NM_001135191 | ArfGAP with SH3 domain, ankyrin repeat and PH domain 2 |
| RBM11 | NM_144770 | RNA binding motif protein 11 |

| | | |
|---|---|---|
| ZC3HAV1L | NM_080660 | zinc finger CCCH-type, antiviral 1-like |
| RBMS1 | NM_002897 | RNA binding motif, single stranded interacting protein 1 |
| MET | NM_000245 | met proto-oncogene (hepatocyte growth factor receptor) |
| ABTB2 | NM_145804 | ankyrin repeat and BTB (POZ) domain containing 2 |
| IFT80 | NM_001190241 | intraflagellar transport 80 homolog (Chlamydomonas) |
| ZNF705A | NM_001004328 | zinc finger protein 705A |
| ZNF705D | NM_001039615 | zinc finger protein 705D |
| CA3 | NM_005181 | carbonic anhydrase III, muscle specific |
| ASB4 | NM_016116 | ankyrin repeat and SOCS box containing 4 |
| C10orf118 | NM_018017 | chromosome 10 open reading frame 118 |
| C4orf46 | NM_001008393 | chromosome 4 open reading frame 46 |
| TIMM9 | NM_012460 | translocase of inner mitochondrial membrane 9 homolog (yeast) |
| KPNA2 | NM_002266 | karyopherin alpha 2 (RAG cohort 1, importin alpha 1) |
| HSPA13 | NM_006948 | heat shock protein 70kDa family, member 13 |
| POLR3G | NM_006467 | polymerase (RNA) III (DNA directed) polypeptide G (32kD) |
| CHSY1 | NM_014918 | chondroitin sulfate synthase 1 |
| ZBTB43 | NM_001135776 | zinc finger and BTB domain containing 43 |
| GAPVD1 | NM_015635 | GTPase activating protein and VPS9 domains 1 |
| CLDN12 | NM_001185072 | claudin 12 |
| INPP5K | NM_001135642 | inositol polyphosphate-5-phosphatase K |
| MXI1 | NM_001008541 | MAX interactor 1 |
| PNPLA4 | NM_001142389 | patatin-like phospholipase domain containing 4 |
| VPS13A | NM_001018037 | vacuolar protein sorting 13 homolog A (S. cerevisiae) |
| PKIA | NM_006823 | protein kinase (cAMP-dependent, catalytic) inhibitor alpha |
| LIN7C | NM_018362 | lin-7 homolog C (C. elegans) |
| ZIC2 | NM_007129 | Zic family member 2 |
| MAGI2 | NM_012301 | membrane associated guanylate kinase, WW and PDZ domain containing 2 |
| CHERP | NM_006387 | calcium homeostasis endoplasmic reticulum protein |
| MYCBP | NM_012333 | c-myc binding protein |
| ACTR3B | NM_001040135 | ARP3 actin-related protein 3 homolog B (yeast) |
| RNF182 | NM_001165032 | ring finger protein 182 |
| EML6 | NM_001039753 | echinoderm microtubule associated protein like 6 |
| SGPP1 | NM_030791 | sphingosine-1-phosphate phosphatase 1 |
| TPP2 | NM_003291 | tripeptidyl peptidase II |
| IRAK3 | NM_001142523 | interleukin-1 receptor-associated kinase 3 |
| RCCD1 | NM_001017919 | RCC1 domain containing 1 |
| DNHD1 | NM_173589 | dynein heavy chain domain 1 |
| LPPR5 | NM_001010861 | lipid phosphate phosphatase-related protein type 5 |
| WHSC1 | NM_001042424 | Wolf-Hirschhorn syndrome candidate 1 |

| | | |
|---|---|---|
| WDR64 | NM_144625 | WD repeat domain 64 |
| KLF6 | NM_001160124 | Kruppel-like factor 6 |
| ARHGEF7 | NM_001113511 | Rho guanine nucleotide exchange factor (GEF) 7 |
| AMIGO2 | NM_001143668 | adhesion molecule with Ig-like domain 2 |
| BDP1 | NM_018429 | B double prime 1, subunit of RNA polymerase III transcription initiation factor IIIB |
| FAM175A | NM_139076 | family with sequence similarity 175, member A |
| PTTG1IP | NM_004339 | pituitary tumor-transforming 1 interacting protein |
| SP3 | NM_001017371 | Sp3 transcription factor |
| CEP192 | NM_032142 | centrosomal protein 192kDa |
| ZCCHC2 | NM_017742 | zinc finger, CCHC domain containing 2 |
| JPH1 | NM_020647 | junctophilin 1 |
| LDOC1L | NM_032287 | leucine zipper, down-regulated in cancer 1-like |
| ZIC5 | NM_033132 | Zic family member 5 |
| LPL | NM_000237 | lipoprotein lipase |
| SCD | NM_005063 | stearoyl-CoA desaturase (delta-9-desaturase) |
| PRMT6 | NM_018137 | protein arginine methyltransferase 6 |
| MBTD1 | NM_017643 | mbt domain containing 1 |
| PRRX1 | NM_006902 | paired related homeobox 1 |
| KIAA1191 | NM_001079684 | KIAA1191 |
| GORAB | NM_001146039 | golgin, RAB6-interacting |
| PATE2 | NM_212555 | prostate and testis expressed 2 |
| GABRA4 | NM_000809 | gamma-aminobutyric acid (GABA) A receptor, alpha 4 |
| MARK1 | NM_018650 | MAP/microtubule affinity-regulating kinase 1 |
| DGKQ | NM_001347 | diacylglycerol kinase, theta 110kDa |
| ADCY1 | NM_021116 | adenylate cyclase 1 (brain) |
| GCH1 | NM_000161 | GTP cyclohydrolase 1 |
| LDB2 | NM_001130834 | LIM domain binding 2 |
| ORMDL1 | NM_001128150 | ORM1-like 1 (S. cerevisiae) |
| FAM134C | NM_178126 | family with sequence similarity 134, member C |
| KIAA1324L | NM_001142749 | KIAA1324-like |
| P4HA3 | NM_182904 | prolyl 4-hydroxylase, alpha polypeptide III |
| SPRYD3 | NM_032840 | SPRY domain containing 3 |
| HNF4G | NM_004133 | hepatocyte nuclear factor 4, gamma |
| PWP2 | NM_005049 | PWP2 periodic tryptophan protein homolog (yeast) |
| CCL7 | NM_006273 | chemokine (C-C motif) ligand 7 |
| EVI5 | NM_005665 | ecotropic viral integration site 5 |
| SPIN4 | NM_001012968 | spindlin family, member 4 |
| ZNF540 | NM_001172225 | zinc finger protein 540 |
| NR1D2 | NM_001145425 | nuclear receptor subfamily 1, group D, member 2 |

| | | |
|---|---|---|
| C13orf36 | NM_203451 | chromosome 13 open reading frame 36 |
| GCNT4 | NM_016591 | glucosaminyl (N-acetyl) transferase 4, core 2 |
| S100Z | NM_130772 | S100 calcium binding protein Z |
| SETD8 | NM_020382 | SET domain containing (lysine methyltransferase) 8 |
| C20orf20 | NM_018270 | chromosome 20 open reading frame 20 |
| CSMD1 | NM_033225 | CUB and Sushi multiple domains 1 |
| ZNF322A | NM_001242797 | zinc finger protein 322A |
| ZC3H11A | NM_014827 | zinc finger CCCH-type containing 11A |
| GFOD1 | NM_001242629 | glucose-fructose oxidoreductase domain containing 1 |
| RAC1 | NM_006908 | ras-related C3 botulinum toxin substrate 1 (rho family, small GTP binding protein |
| VPS45 | NM_007259 | vacuolar protein sorting 45 homolog (S. cerevisiae) |
| DDX4 | NM_001142549 | DEAD (Asp-Glu-Ala-Asp) box polypeptide 4 |
| TSPAN3 | NM_001168412 | tetraspanin 3 |
| ARFIP1 | NM_001025593 | ADP-ribosylation factor interacting protein 1 |
| WNK1 | NM_001184985 | WNK lysine deficient protein kinase 1 |
| NSMCE4A | NM_001167865 | non-SMC element 4 homolog A (S. cerevisiae) |
| AFF1 | NM_001166693 | AF4/FMR2 family, member 1 |
| C1orf55 | NM_152608 | chromosome 1 open reading frame 55 |
| ANKS1B | NM_001204065 | ankyrin repeat and sterile alpha motif domain containing 1B |
| PSMG2 | NM_020232 | proteasome (prosome, macropain) assembly chaperone 2 |
| ANO3 | NM_031418 | anoctamin 3 |
| LRRCC1 | NM_033402 | leucine rich repeat and coiled-coil domain containing 1 |
| ZNF563 | NM_145276 | zinc finger protein 563 |
| ZYG11A | NM_001004339 | zyg-11 homolog A (C. elegans) |
| CPD | NM_001199775 | carboxypeptidase D |
| FUNDC2 | NM_023934 | FUN14 domain containing 2 |
| PPM1A | NM_021003 | protein phosphatase, Mg2+/Mn2+ dependent, 1A |
| STXBP3 | NM_007269 | syntaxin binding protein 3 |
| KIAA1429 | NM_015496 | KIAA1429 |
| PCMTD2 | NM_001104925 | protein-L-isoaspartate (D-aspartate) O-methyltransferase domain containing 2 |
| RFTN2 | NM_144629 | raftlin family member 2 |
| BEND3 | NM_001080450 | BEN domain containing 3 |
| ABI2 | NM_005759 | abl-interactor 2 |
| CD82 | NM_001024844 | CD82 molecule |
| XPO7 | NM_015024 | exportin 7 |
| ZNF285 | NM_152354 | zinc finger protein 285 |
| ZNF682 | NM_001077349 | zinc finger protein 682 |
| BMI1 | NM_005180 | BMI1 polycomb ring finger oncogene |
| GFPT1 | NM_002056 | glutamine--fructose-6-phosphate transaminase 1 |

| | | |
|---|---|---|
| SGSM2 | NM_001098509 | small G protein signaling modulator 2 |
| LRRC7 | NM_020794 | leucine rich repeat containing 7 |
| COMMD3-BMI1 | NM_001204062 | COMMD3-BMI1 readthrough |
| HELZ | NM_014877 | helicase with zinc finger |
| SPTBN1 | NM_178313 | spectrin, beta, non-erythrocytic 1 |
| SERAC1 | NM_032861 | serine active site containing 1 |
| SRP9 | NM_001130440 | signal recognition particle 9kDa |
| ZNF507 | NM_001136156 | zinc finger protein 507 |
| ITIH3 | NM_002217 | inter-alpha (globulin) inhibitor H3 |
| PDXK | NM_003681 | pyridoxal (pyridoxine, vitamin B6) kinase |
| FAM179B | NM_015091 | family with sequence similarity 179, member B |
| NLK | NM_016231 | nemo-like kinase |
| IL28RA | NM_170743 | interleukin 28 receptor, alpha (interferon, lambda receptor) |
| DDX3X | NM_001193416 | DEAD (Asp-Glu-Ala-Asp) box polypeptide 3, X-linked |
| SRPRB | NM_021203 | signal recognition particle receptor, B subunit |
| MPZL2 | NM_005797 | myelin protein zero-like 2 |
| EPB41L4B | NM_019114 | erythrocyte membrane protein band 4.1 like 4B |
| CD274 | NM_014143 | CD274 molecule |
| RGS14 | NM_006480 | regulator of G-protein signaling 14 |
| STMN2 | NM_001199214 | stathmin-like 2 |
| PLAC8 | NM_001130715 | placenta-specific 8 |
| SLC46A3 | NM_181785 | solute carrier family 46, member 3 |
| MAGOHB | NM_018048 | mago-nashi homolog B (Drosophila) |
| MYBL1 | NM_001080416 | v-myb myeloblastosis viral oncogene homolog (avian)-like 1 |
| C15orf41 | NM_001130010 | chromosome 15 open reading frame 41 |
| IDI1 | NM_004508 | isopentenyl-diphosphate delta isomerase 1 |
| WASL | NM_003941 | Wiskott-Aldrich syndrome-like |
| LRAT | NM_004744 | lecithin retinol acyltransferase (phosphatidylcholine--retinol O-acyltransferase) |
| SNRNP40 | NM_004814 | small nuclear ribonucleoprotein 40kDa (U5) |
| RBM7 | NM_016090 | RNA binding motif protein 7 |
| TMEM87A | NM_001110503 | transmembrane protein 87A |
| WAC | NM_016628 | WW domain containing adaptor with coiled-coil |
| PHTF2 | NM_001127359 | putative homeodomain transcription factor 2 |
| GDA | NM_001242505 | guanine deaminase |
| CDCA7L | NM_001127370 | cell division cycle associated 7-like |
| TRMT11 | NM_001031712 | tRNA methyltransferase 11 homolog (S. cerevisiae) |
| RGS5 | NM_001195303 | regulator of G-protein signaling 5 |
| DOK6 | NM_152721 | docking protein 6 |
| SLC26A2 | NM_000112 | solute carrier family 26 (sulfate transporter), member 2 |

| | | |
|---|---|---|
| ZMYND12 | NM_001146192 | zinc finger, MYND-type containing 12 |
| TMEM215 | NM_212558 | transmembrane protein 215 |
| COBLL1 | NM_014900 | COBL-like 1 |
| NAT8L | NM_178557 | N-acetyltransferase 8-like (GCN5-related, putative) |
| NOTCH2 | NM_024408 | notch 2 |
| KIAA1407 | NM_020817 | KIAA1407 |
| APLF | NM_173545 | aprataxin and PNKP like factor |
| PSMD7 | NM_002811 | proteasome (prosome, macropain) 26S subunit, non-ATPase, 7 |
| CDC14A | NM_003672 | CDC14 cell division cycle 14 homolog A (S. cerevisiae) |
| ADAMTS3 | NM_014243 | ADAM metallopeptidase with thrombospondin type 1 motif, 3 |
| LOC221710 | NM_001135575 | hypothetical protein LOC221710 |
| RIN2 | NM_001242581 | Ras and Rab interactor 2 |
| ITPRIPL2 | NM_001034841 | inositol 1,4,5-trisphosphate receptor interacting protein-like 2 |
| C4orf33 | NM_001099783 | chromosome 4 open reading frame 33 |
| ALS2CR8 | NM_001104586 | amyotrophic lateral sclerosis 2 (juvenile) chromosome region, candidate 8 |
| TMEM65 | NM_194291 | transmembrane protein 65 |
| PHKG2 | NM_000294 | phosphorylase kinase, gamma 2 (testis) |
| C20orf3 | NM_020531 | chromosome 20 open reading frame 3 |
| OLIG3 | NM_175747 | oligodendrocyte transcription factor 3 |
| GIMAP6 | NM_024711 | GTPase, IMAP family member 6 |
| MS4A1 | NM_021950 | membrane-spanning 4-domains, subfamily A, member 1 |
| TAC1 | NM_003182 | tachykinin, precursor 1 |
| FAM60A | NM_001135811 | family with sequence similarity 60, member A |
| AQP9 | NM_020980 | aquaporin 9 |
| PDE5A | NM_001083 | phosphodiesterase 5A, cGMP-specific |
| TDRKH | NM_001083963 | tudor and KH domain containing |
| FOXK1 | NM_001037165 | forkhead box K1 |
| ZNF470 | NM_001001668 | zinc finger protein 470 |
| RYBP | NM_012234 | RING1 and YY1 binding protein |
| PTMA | NM_001099285 | prothymosin, alpha |
| CCR2 | NM_001123041 | chemokine (C-C motif) receptor 2 |
| POM121C | NM_001099415 | POM121 membrane glycoprotein C |
| NR3C1 | NM_000176 | nuclear receptor subfamily 3, group C, member 1 (glucocorticoid receptor) |
| RHOA | NM_001664 | ras homolog gene family, member A |
| PTPRM | NM_001105244 | protein tyrosine phosphatase, receptor type, M |
| RFX3 | NM_134428 | regulatory factor X, 3 (influences HLA class II expression) |
| SPARCL1 | NM_001128310 | SPARC-like 1 (hevin) |
| RNF44 | NM_014901 | ring finger protein 44 |
| AKIP1 | NM_001206645 | A kinase (PRKA) interacting protein 1 |

| | | |
|---|---|---|
| CCDC150 | NM_001080539 | coiled-coil domain containing 150 |
| STXBP4 | NM_178509 | syntaxin binding protein 4 |
| WDR41 | NM_018268 | WD repeat domain 41 |
| AGPAT5 | NM_018361 | 1-acylglycerol-3-phosphate O-acyltransferase 5 (lysophosphatidic acid |
| HS3ST4 | NM_006040 | heparan sulfate (glucosamine) 3-O-sulfotransferase 4 |
| LRCH1 | NM_001164211 | leucine-rich repeats and calponin homology (CH) domain containing 1 |
| SPTY2D1 | NM_194285 | SPT2, Suppressor of Ty, domain containing 1 (S. cerevisiae) |
| AKR7L | NM_001145289 | aldo-keto reductase family 7-like |
| MTMR3 | NM_021090 | myotubularin related protein 3 |
| MEGF9 | NM_001080497 | multiple EGF-like-domains 9 |
| PRDX1 | NM_001202431 | peroxiredoxin 1 |
| VEZF1 | NM_007146 | vascular endothelial zinc finger 1 |
| LRRTM2 | NM_015564 | leucine rich repeat transmembrane neuronal 2 |
| TMEM206 | NM_001198862 | transmembrane protein 206 |
| MRPS14 | NM_022100 | mitochondrial ribosomal protein S14 |
| CCDC73 | NM_001008391 | coiled-coil domain containing 73 |
| ATF7 | NM_001130059 | activating transcription factor 7 |
| PTGER2 | NM_000956 | prostaglandin E receptor 2 (subtype EP2), 53kDa |
| FTSJD1 | NM_001099642 | FtsJ methyltransferase domain containing 1 |
| IMPA1 | NM_001144878 | inositol(myo)-1(or 4)-monophosphatase 1 |
| TAF1 | NM_004606 | TAF1 RNA polymerase II, TATA box binding protein (TBP)-associated factor, 250kDa |
| MLYCD | NM_012213 | malonyl-CoA decarboxylase |
| CCDC50 | NM_174908 | coiled-coil domain containing 50 |
| GEMC1 | NM_001146686 | geminin coiled-coil domain-containing protein 1 |
| CCDC165 | NM_015210 | coiled-coil domain containing 165 |
| C2orf67 | NM_152519 | chromosome 2 open reading frame 67 |
| CPEB1 | NM_001079533 | cytoplasmic polyadenylation element binding protein 1 |
| ZNF260 | NM_001012756 | zinc finger protein 260 |
| NUDT4 | NM_019094 | nudix (nucleoside diphosphate linked moiety X)-type motif 4 |
| APOBEC3F | NM_145298 | apolipoprotein B mRNA editing enzyme, catalytic polypeptide-like 3F |
| CSF2RB | NM_000395 | colony stimulating factor 2 receptor, beta, low-affinity (granulocyte-macrophage) |
| PDE3B | NM_000922 | phosphodiesterase 3B, cGMP-inhibited |
| MATN2 | NM_002380 | matrilin 2 |
| NKX3-1 | NM_006167 | NK3 homeobox 1 |
| TOP1 | NM_003286 | topoisomerase (DNA) I |
| CYorf15B | NM_032576 | chromosome Y open reading frame 15B |
| ZNF493 | NM_145326 | zinc finger protein 493 |
| ISPD | NM_001101417 | isoprenoid synthase domain containing |
| GCNT1 | NM_001097633 | glucosaminyl (N-acetyl) transferase 1, core 2 |

| | | |
|---|---|---|
| ZNF33A | NM_006954 | zinc finger protein 33A |
| C7orf52 | NM_198571 | chromosome 7 open reading frame 52 |
| SLC16A12 | NM_213606 | solute carrier family 16, member 12 (monocarboxylic acid transporter 12) |
| ADAMTS5 | NM_007038 | ADAM metallopeptidase with thrombospondin type 1 motif, 5 |
| POM121 | NM_172020 | POM121 membrane glycoprotein |
| UBE3B | NM_130466 | ubiquitin protein ligase E3B |
| KIAA1217 | NM_001098500 | KIAA1217 |
| MAPKAPK2 | NM_004759 | mitogen-activated protein kinase-activated protein kinase 2 |
| MAGI3 | NM_152900 | membrane associated guanylate kinase, WW and PDZ domain containing 3 |
| CRHBP | NM_001882 | corticotropin releasing hormone binding protein |
| FCRLA | NM_001184866 | Fc receptor-like A |
| KLHL32 | NM_052904 | kelch-like 32 (Drosophila) |
| FCHO2 | NM_001146032 | FCH domain only 2 |
| GEN1 | NM_001130009 | Gen homolog 1, endonuclease (Drosophila) |
| RET | NM_020975 | ret proto-oncogene |
| BRD3 | NM_007371 | bromodomain containing 3 |
| TMEM119 | NM_181724 | transmembrane protein 119 |
| PJA1 | NM_001032396 | praja ring finger 1 |
| ARHGAP29 | NM_004815 | Rho GTPase activating protein 29 |
| UBE2B | NM_003337 | ubiquitin-conjugating enzyme E2B |
| SEMA3C | NM_006379 | sema domain, immunoglobulin domain (Ig), short basic domain, secreted, |
| RPP14 | NM_001098783 | ribonuclease P/MRP 14kDa subunit |
| KDELC2 | NM_153705 | KDEL (Lys-Asp-Glu-Leu) containing 2 |
| EDNRA | NM_001166055 | endothelin receptor type A |
| TNRC6A | NM_014494 | trinucleotide repeat containing 6A |
| IL20RB | NM_144717 | interleukin 20 receptor beta |
| MTMR10 | NM_017762 | myotubularin related protein 10 |
| HECTD2 | NM_182765 | HECT domain containing 2 |
| MARCH5 | NM_017824 | membrane-associated ring finger (C3HC4) 5 |
| TUB | NM_003320 | tubby homolog (mouse) |
| MLLT4 | NM_001207008 | myeloid/lymphoid or mixed-lineage leukemia (trithorax homolog, Drosophila); |
| DBT | NM_001918 | dihydrolipoamide branched chain transacylase E2 |
| UCHL5 | NM_001199261 | ubiquitin carboxyl-terminal hydrolase L5 |
| CCDC85A | NM_001080433 | coiled-coil domain containing 85A |
| FIBIN | NM_203371 | fin bud initiation factor homolog (zebrafish) |
| TLK1 | NM_001136554 | tousled-like kinase 1 |
| ZNRF2 | NM_147128 | zinc and ring finger 2 |
| CYP7A1 | NM_000780 | cytochrome P450, family 7, subfamily A, polypeptide 1 |
| LOC100500938 | NM_001195637 | hypothetical LOC100500938 |

| | | |
|---|---|---|
| SPATA13 | NM_001166271 | spermatogenesis associated 13 |
| GAS1 | NM_002048 | growth arrest-specific 1 |
| FAM3C | NM_001040020 | family with sequence similarity 3, member C |
| DCAF10 | NM_024345 | DDB1 and CUL4 associated factor 10 |
| IL11 | NM_000641 | interleukin 11 |
| ZBTB11 | NM_014415 | zinc finger and BTB domain containing 11 |
| EXOC2 | NM_018303 | exocyst complex component 2 |
| MBL2 | NM_000242 | mannose-binding lectin (protein C) 2, soluble |
| BAAT | NM_001127610 | bile acid CoA: amino acid N-acyltransferase (glycine N-choloyltransferase) |
| NBEA | NM_001204197 | neurobeachin |
| DUSP19 | NM_001142314 | dual specificity phosphatase 19 |
| CCDC125 | NM_176816 | coiled-coil domain containing 125 |
| GPR183 | NM_004951 | G protein-coupled receptor 183 |
| PRPSAP2 | NM_002767 | phosphoribosyl pyrophosphate synthetase-associated protein 2 |
| XRCC4 | NM_003401 | X-ray repair complementing defective repair in Chinese hamster cells 4 |
| KIAA0753 | NM_014804 | KIAA0753 |
| C1orf56 | NM_017860 | chromosome 1 open reading frame 56 |
| AGBL2 | NM_024783 | ATP/GTP binding protein-like 2 |
| LRR1 | NM_152329 | leucine rich repeat protein 1 |
| FAM69C | NM_001044369 | family with sequence similarity 69, member C |
| ATRNL1 | NM_207303 | attractin-like 1 |
| HPSE2 | NM_001166244 | heparanase 2 |
| PMP2 | NM_002677 | peripheral myelin protein 2 |
| ATP6V1A | NM_001690 | ATPase, H+ transporting, lysosomal 70kDa, V1 subunit A |
| MYH11 | NM_001040113 | myosin, heavy chain 11, smooth muscle |
| RAB4A | NM_004578 | RAB4A, member RAS oncogene family |
| MRPS18C | NM_016067 | mitochondrial ribosomal protein S18C |
| SNX7 | NM_015976 | sorting nexin 7 |
| DNASE1L1 | NM_001009932 | deoxyribonuclease I-like 1 |
| LIG4 | NM_001098268 | ligase IV, DNA, ATP-dependent |
| IGF2BP3 | NM_006547 | insulin-like growth factor 2 mRNA binding protein 3 |
| ASCC3 | NM_022091 | activating signal cointegrator 1 complex subunit 3 |
| SEMA3D | NM_152754 | sema domain, immunoglobulin domain (Ig), short basic domain, secreted, |
| ACSL6 | NM_001009185 | acyl-CoA synthetase long-chain family member 6 |
| CLCN6 | NM_001286 | chloride channel 6 |
| CDK14 | NM_012395 | cyclin-dependent kinase 14 |
| GGPS1 | NM_001037277 | geranylgeranyl diphosphate synthase 1 |
| FLJ44635 | NM_207422 | TPT1-like protein |
| PPP1R12A | NM_001143885 | protein phosphatase 1, regulatory (inhibitor) subunit 12A |

| | | |
|---|---|---|
| PAQR3 | NM_001040202 | progestin and adipoQ receptor family member III |
| ODAM | NM_017855 | odontogenic, ameloblast asssociated |
| ABCA13 | NM_152701 | ATP-binding cassette, sub-family A (ABC1), member 13 |
| RHOU | NM_021205 | ras homolog gene family, member U |
| GIGYF2 | NM_001103146 | GRB10 interacting GYF protein 2 |
| EAF1 | NM_033083 | ELL associated factor 1 |
| GDPD1 | NM_182569 | glycerophosphodiester phosphodiesterase domain containing 1 |
| TICAM2 | NM_021649 | toll-like receptor adaptor molecule 2 |
| TMED7-TICAM2 | NM_001164468 | TMED7-TICAM2 readthrough |
| LIN54 | NM_001115007 | lin-54 homolog (C. elegans) |
| KIAA1430 | NM_020827 | KIAA1430 |
| DCAF17 | NM_001164821 | DDB1 and CUL4 associated factor 17 |
| NOL6 | NM_022917 | nucleolar protein family 6 (RNA-associated) |
| SH3BGRL | NM_003022 | SH3 domain binding glutamic acid-rich protein like |
| BAALC | NM_001024372 | brain and acute leukemia, cytoplasmic |
| FAM124A | NM_001242312 | family with sequence similarity 124A |
| TMEM106B | NM_001134232 | transmembrane protein 106B |
| PLDN | NM_012388 | pallidin homolog (mouse) |
| SEC23IP | NM_007190 | SEC23 interacting protein |
| STK4 | NM_006282 | serine/threonine kinase 4 |
| PSAT1 | NM_021154 | phosphoserine aminotransferase 1 |
| PLA2G3 | NM_015715 | phospholipase A2, group III |
| CCDC80 | NM_199511 | coiled-coil domain containing 80 |
| PRPF39 | NM_017922 | PRP39 pre-mRNA processing factor 39 homolog (S. cerevisiae) |
| SP1 | NM_003109 | Sp1 transcription factor |
| ACTN4 | NM_004924 | actinin, alpha 4 |
| CTLA4 | NM_001037631 | cytotoxic T-lymphocyte-associated protein 4 |
| ZSWIM6 | NM_020928 | zinc finger, SWIM-type containing 6 |
| C9orf68 | NM_001039395 | chromosome 9 open reading frame 68 |
| PLAC8L1 | NM_001029869 | PLAC8-like 1 |
| SMAD9 | NM_001127217 | SMAD family member 9 |
| CNTN3 | NM_020872 | contactin 3 (plasmacytoma associated) |
| GABPB1 | NM_005254 | GA binding protein transcription factor, beta subunit 1 |
| JKAMP | NM_001098625 | JNK1/MAPK8-associated membrane protein |
| ERAP2 | NM_001130140 | endoplasmic reticulum aminopeptidase 2 |
| TPRG1 | NM_198485 | tumor protein p63 regulated 1 |
| CRBN | NM_001173482 | cereblon |
| RAD54L | NM_001142548 | RAD54-like (S. cerevisiae) |
| PARP9 | NM_001146102 | poly (ADP-ribose) polymerase family, member 9 |

| | | |
|---|---|---|
| KIAA0825 | NM_001145678 | KIAA0825 |
| CCNT2 | NM_001241 | cyclin T2 |
| KIAA0415 | NM_014855 | KIAA0415 |
| FRAT2 | NM_012083 | frequently rearranged in advanced T-cell lymphomas 2 |
| NEURL1B | NM_001142651 | neuralized homolog 1B (Drosophila) |
| RBM25 | NM_021239 | RNA binding motif protein 25 |
| SLC25A4 | NM_001151 | solute carrier family 25 (mitochondrial carrier; adenine nucleotide translocator), |
| SRCAP | NM_006662 | Snf2-related CREBBP activator protein |
| SEPT8 | NM_001098811 | septin 8 |
| SOCS4 | NM_080867 | suppressor of cytokine signaling 4 |
| NKAIN2 | NM_001040214 | Na+/K+ transporting ATPase interacting 2 |
| SPO11 | NM_012444 | SPO11 meiotic protein covalently bound to DSB homolog (S. cerevisiae) |
| RUNX1T1 | NM_001198625 | runt-related transcription factor 1; translocated to, 1 (cyclin D-related) |
| GRIA4 | NM_000829 | glutamate receptor, ionotrophic, AMPA 4 |
| PHF1 | NM_002636 | PHD finger protein 1 |
| CCDC41 | NM_001042399 | coiled-coil domain containing 41 |
| LPPR1 | NM_017753 | lipid phosphate phosphatase-related protein type 1 |
| SMC1A | NM_006306 | structural maintenance of chromosomes 1A |
| ZNF678 | NM_178549 | zinc finger protein 678 |
| ZSWIM7 | NM_001042697 | zinc finger, SWIM-type containing 7 |
| ZNF367 | NM_153695 | zinc finger protein 367 |
| ZER1 | NM_006336 | zer-1 homolog (C. elegans) |
| RNF19A | NM_015435 | ring finger protein 19A |
| TMEM48 | NM_001168551 | transmembrane protein 48 |
| TMX1 | NM_030755 | thioredoxin-related transmembrane protein 1 |
| UTP23 | NM_032334 | UTP23, small subunit (SSU) processome component, homolog (yeast) |
| RG9MTD3 | NM_144964 | RNA (guanine-9-) methyltransferase domain containing 3 |
| C14orf129 | NM_016472 | chromosome 14 open reading frame 129 |
| DPP10 | NM_001004360 | dipeptidyl-peptidase 10 (non-functional) |
| ELK4 | NM_001973 | ELK4, ETS-domain protein (SRF accessory protein 1) |
| PRC1 | NM_003981 | protein regulator of cytokinesis 1 |
| DDHD2 | NM_001164232 | DDHD domain containing 2 |
| PDE4D | NM_001104631 | phosphodiesterase 4D, cAMP-specific |
| SRSF8 | NM_032102 | serine/arginine-rich splicing factor 8 |
| FXR2 | NM_004860 | fragile X mental retardation, autosomal homolog 2 |
| CISD1 | NM_018464 | CDGSH iron sulfur domain 1 |
| PRDM15 | NM_001040424 | PR domain containing 15 |
| C15orf61 | NM_001143936 | chromosome 15 open reading frame 61 |
| LEKR1 | NM_001193283 | leucine, glutamate and lysine rich 1 |

| | | |
|---|---|---|
| ZFAND3 | NM_021943 | zinc finger, AN1-type domain 3 |
| ALG10B | NM_001013620 | asparagine-linked glycosylation 10, alpha-1,2-glucosyltransferase homolog B (yeast) |
| C22orf13 | NM_031444 | chromosome 22 open reading frame 13 |
| CHKA | NM_001277 | choline kinase alpha |
| MAGEA8 | NM_001166400 | melanoma antigen family A, 8 |
| MEIS2 | NM_001220482 | Meis homeobox 2 |
| TLR1 | NM_003263 | toll-like receptor 1 |
| GPR176 | NM_007223 | G protein-coupled receptor 176 |
| TIMM8B | NM_012459 | translocase of inner mitochondrial membrane 8 homolog B (yeast) |
| ANKRD50 | NM_001167882 | ankyrin repeat domain 50 |
| MORC4 | NM_001085354 | MORC family CW-type zinc finger 4 |
| PDZD3 | NM_001168468 | PDZ domain containing 3 |
| ZNF230 | NM_006300 | zinc finger protein 230 |
| C1orf43 | NM_001098616 | chromosome 1 open reading frame 43 |
| LANCL1 | NM_001136574 | LanC lantibiotic synthetase component C-like 1 (bacterial) |
| PPP1R14C | NM_030949 | protein phosphatase 1, regulatory (inhibitor) subunit 14C |
| ERAP1 | NM_016442 | endoplasmic reticulum aminopeptidase 1 |
| PDCD2 | NM_001199461 | programmed cell death 2 |
| PHKB | NM_000293 | phosphorylase kinase, beta |
| HPCAL4 | NM_016257 | hippocalcin like 4 |
| ZNF490 | NM_020714 | zinc finger protein 490 |
| ABHD13 | NM_032859 | abhydrolase domain containing 13 |
| PRPF40A | NM_017892 | PRP40 pre-mRNA processing factor 40 homolog A (S. cerevisiae) |
| ZNF33B | NM_006955 | zinc finger protein 33B |
| PPP1R13B | NM_015316 | protein phosphatase 1, regulatory (inhibitor) subunit 13B |
| PJA2 | NM_014819 | praja ring finger 2 |
| ZBTB40 | NM_001083621 | zinc finger and BTB domain containing 40 |
| KIAA0232 | NM_001100590 | KIAA0232 |
| API5 | NM_001142930 | apoptosis inhibitor 5 |
| ARID4B | NM_001206794 | AT rich interactive domain 4B (RBP1-like) |
| BCL11B | NM_022898 | B-cell CLL/lymphoma 11B (zinc finger protein) |
| ZNF605 | NM_001164715 | zinc finger protein 605 |
| ARL4A | NM_001037164 | ADP-ribosylation factor-like 4A |
| MBTPS2 | NM_015884 | membrane-bound transcription factor peptidase, site 2 |
| CBX5 | NM_001127321 | chromobox homolog 5 |
| SPATA5 | NM_145207 | spermatogenesis associated 5 |
| JAG2 | NM_002226 | jagged 2 |
| LOC100507462 | NM_001242740 | hypothetical LOC100507462 |
| ALDH1A1 | NM_000689 | aldehyde dehydrogenase 1 family, member A1 |

| | | |
|---|---|---|
| CEACAM7 | NM_006890 | carcinoembryonic antigen-related cell adhesion molecule 7 |
| ZBED4 | NM_014838 | zinc finger, BED-type containing 4 |
| VPS24 | NM_001005753 | vacuolar protein sorting 24 homolog (S. cerevisiae) |
| MLL5 | NM_018682 | myeloid/lymphoid or mixed-lineage leukemia 5 (trithorax homolog, Drosophila) |
| RNF103-VPS24 | NM_001198954 | RNF103-VPS24 readthrough |
| NR0B2 | NM_021969 | nuclear receptor subfamily 0, group B, member 2 |
| C15orf56 | NM_001039905 | chromosome 15 open reading frame 56 |
| PURB | NM_033224 | purine-rich element binding protein B |
| FKBP5 | NM_001145777 | FK506 binding protein 5 |
| PFKFB2 | NM_001018053 | 6-phosphofructo-2-kinase/fructose-2,6-biphosphatase 2 |
| FGF9 | NM_002010 | fibroblast growth factor 9 (glia-activating factor) |
| RAG1 | NM_000448 | recombination activating gene 1 |
| OR11A1 | NM_013937 | olfactory receptor, family 11, subfamily A, member 1 |
| SMAP1 | NM_001044305 | small ArfGAP 1 |
| SMARCE1 | NM_003079 | SWI/SNF related, matrix associated, actin dependent regulator of chromatin, |
| ZNF69 | NM_021915 | zinc finger protein 69 |
| NUMB | NM_001005743 | numb homolog (Drosophila) |
| CERKL | NM_001030311 | ceramide kinase-like |
| NFATC1 | NM_006162 | nuclear factor of activated T-cells, cytoplasmic, calcineurin-dependent 1 |
| RNF122 | NM_024787 | ring finger protein 122 |
| SOAT1 | NM_003101 | sterol O-acyltransferase 1 |
| TFAP2C | NM_003222 | transcription factor AP-2 gamma (activating enhancer binding protein 2 gamma) |
| CD2AP | NM_012120 | CD2-associated protein |
| WLS | NM_001193334 | wntless homolog (Drosophila) |
| ABRA | NM_139166 | actin-binding Rho activating protein |
| SATB1 | NM_001131010 | SATB homeobox 1 |
| SLU7 | NM_006425 | SLU7 splicing factor homolog (S. cerevisiae) |
| EPT1 | NM_033505 | ethanolaminephosphotransferase 1 (CDP-ethanolamine-specific) |
| NDFIP1 | NM_030571 | Nedd4 family interacting protein 1 |
| GABRP | NM_014211 | gamma-aminobutyric acid (GABA) A receptor, pi |
| NEDD4 | NM_006154 | neural precursor cell expressed, developmentally down-regulated 4 |
| PTPRF | NM_002840 | protein tyrosine phosphatase, receptor type, F |
| SLC39A14 | NM_001128431 | solute carrier family 39 (zinc transporter), member 14 |
| SFXN2 | NM_178858 | sideroflexin 2 |
| CCDC15 | NM_025004 | coiled-coil domain containing 15 |
| ZC3H12B | NM_001010888 | zinc finger CCCH-type containing 12B |
| FUT9 | NM_006581 | fucosyltransferase 9 (alpha (1,3) fucosyltransferase) |
| SENP1 | NM_014554 | SUMO1/sentrin specific peptidase 1 |
| PCNP | NM_020357 | PEST proteolytic signal containing nuclear protein |

| | | |
|---|---|---|
| CDC27 | NM_001114091 | cell division cycle 27 homolog (S. cerevisiae) |
| ITPK1 | NM_001142594 | inositol-tetrakisphosphate 1-kinase |
| GLRA3 | NM_001042543 | glycine receptor, alpha 3 |
| BAG5 | NM_001015048 | BCL2-associated athanogene 5 |
| SLC22A15 | NM_018420 | solute carrier family 22, member 15 |
| UPP2 | NM_001135098 | uridine phosphorylase 2 |
| CYP20A1 | NM_177538 | cytochrome P450, family 20, subfamily A, polypeptide 1 |
| PRTG | NM_173814 | protogenin |
| FOXN2 | NM_002158 | forkhead box N2 |
| RFC5 | NM_001130112 | replication factor C (activator 1) 5, 36.5kDa |
| SGCG | NM_000231 | sarcoglycan, gamma (35kDa dystrophin-associated glycoprotein) |
| USP14 | NM_001037334 | ubiquitin specific peptidase 14 (tRNA-guanine transglycosylase) |
| SWAP70 | NM_015055 | SWAP switching B-cell complex 70kDa subunit |
| CNOT6 | NM_015455 | CCR4-NOT transcription complex, subunit 6 |
| EHHADH | NM_001166415 | enoyl-CoA, hydratase/3-hydroxyacyl CoA dehydrogenase |
| HNRPDL | NM_001207000 | heterogeneous nuclear ribonucleoprotein D-like |
| BOD1L | NM_148894 | biorientation of chromosomes in cell division 1-like |
| GADD45A | NM_001199741 | growth arrest and DNA-damage-inducible, alpha |
| TGFB3 | NM_003239 | transforming growth factor, beta 3 |
| TRPC3 | NM_001130698 | transient receptor potential cation channel, subfamily C, member 3 |
| NT5DC3 | NM_001031701 | 5'-nucleotidase domain containing 3 |
| RAB39B | NM_171998 | RAB39B, member RAS oncogene family |
| LIX1 | NM_153234 | Lix1 homolog (chicken) |
| SZT2 | NM_015284 | seizure threshold 2 homolog (mouse) |
| RNLS | NM_018363 | renalase, FAD-dependent amine oxidase |
| BOD1 | NM_001159651 | biorientation of chromosomes in cell division 1 |
| NAA16 | NM_024561 | N(alpha)-acetyltransferase 16, NatA auxiliary subunit |
| SEMA7A | NM_001146029 | semaphorin 7A, GPI membrane anchor (John Milton Hagen blood group) |
| ZNF532 | NM_018181 | zinc finger protein 532 |
| CUX1 | NM_001202543 | cut-like homeobox 1 |
| ARHGEF38 | NM_001242729 | Rho guanine nucleotide exchange factor (GEF) 38 |
| MTHFD2 | NM_006636 | methylenetetrahydrofolate dehydrogenase (NADP+ dependent) 2, methenyltetrahydrofolate cyclohydrolase |
| FIP1L1 | NM_001134937 | FIP1 like 1 (S. cerevisiae) |
| HIATL1 | NM_032558 | hippocampus abundant transcript-like 1 |
| C14orf126 | NM_080664 | chromosome 14 open reading frame 126 |
| DNMT1 | NM_001130823 | DNA (cytosine-5-)-methyltransferase 1 |
| SLC25A12 | NM_003705 | solute carrier family 25 (mitochondrial carrier, Aralar), member 12 |
| CPLX2 | NM_001008220 | complexin 2 |

| | | |
|---|---|---|
| ZNF440 | NM_152357 | zinc finger protein 440 |
| NALCN | NM_052867 | sodium leak channel, non-selective |
| FUT1 | NM_000148 | fucosyltransferase 1 (galactoside 2-alpha-L-fucosyltransferase, H blood group) |
| GNG5 | NM_005274 | guanine nucleotide binding protein (G protein), gamma 5 |
| ZNF32 | NM_001005368 | zinc finger protein 32 |
| KIAA1737 | NM_033426 | KIAA1737 |
| METTL20 | NM_001135863 | methyltransferase like 20 |
| GNL3L | NM_001184819 | guanine nucleotide binding protein-like 3 (nucleolar)-like |
| PCDHA9 | NM_031857 | protocadherin alpha 9 |
| PCDHAC2 | NM_018899 | protocadherin alpha subfamily C, 2 |
| PCDHAC1 | NM_018898 | protocadherin alpha subfamily C, 1 |
| PCDHA13 | NM_018904 | protocadherin alpha 13 |
| PCDHA12 | NM_018903 | protocadherin alpha 12 |
| PCDHA11 | NM_018902 | protocadherin alpha 11 |
| PCDHA10 | NM_018901 | protocadherin alpha 10 |
| PCDHA8 | NM_018911 | protocadherin alpha 8 |
| PCDHA7 | NM_018910 | protocadherin alpha 7 |
| PCDHA6 | NM_018909 | protocadherin alpha 6 |
| PCDHA5 | NM_018908 | protocadherin alpha 5 |
| PCDHA4 | NM_018907 | protocadherin alpha 4 |
| PCDHA3 | NM_018906 | protocadherin alpha 3 |
| PCDHA2 | NM_018905 | protocadherin alpha 2 |
| PCDHA1 | NM_018900 | protocadherin alpha 1 |
| ACYP1 | NM_001107 | acylphosphatase 1, erythrocyte (common) type |
| CCR1 | NM_001295 | chemokine (C-C motif) receptor 1 |
| DNMT3A | NM_175630 | DNA (cytosine-5-)-methyltransferase 3 alpha |
| GINS4 | NM_032336 | GINS complex subunit 4 (Sld5 homolog) |
| ZNF561 | NM_152289 | zinc finger protein 561 |
| AGAP3 | NM_031946 | ArfGAP with GTPase domain, ankyrin repeat and PH domain 3 |
| IL36RN | NM_012275 | interleukin 36 receptor antagonist |
| NUS1 | NM_138459 | nuclear undecaprenyl pyrophosphate synthase 1 homolog (S. cerevisiae) |
| USP13 | NM_003940 | ubiquitin specific peptidase 13 (isopeptidase T-3) |
| USP44 | NM_001042403 | ubiquitin specific peptidase 44 |
| MMP20 | NM_004771 | matrix metallopeptidase 20 |
| TMOD2 | NM_001142885 | tropomodulin 2 (neuronal) |
| FNDC3B | NM_001135095 | fibronectin type III domain containing 3B |
| LOC728819 | NM_001101330 | hCG1645220 |
| CYFIP2 | NM_001037332 | cytoplasmic FMR1 interacting protein 2 |
| LYN | NM_001111097 | v-yes-1 Yamaguchi sarcoma viral related oncogene homolog |

| | | |
|---|---|---|
| SYT4 | NM_020783 | synaptotagmin IV |
| CLIC5 | NM_001114086 | chloride intracellular channel 5 |
| ZNF434 | NM_017810 | zinc finger protein 434 |
| ZNF665 | NM_024733 | zinc finger protein 665 |
| EDNRB | NM_000115 | endothelin receptor type B |
| DOPEY2 | NM_005128 | dopey family member 2 |
| LIPH | NM_139248 | lipase, member H |
| C7orf42 | NM_017994 | chromosome 7 open reading frame 42 |
| TRIM35 | NM_171982 | tripartite motif containing 35 |
| DOK5 | NM_018431 | docking protein 5 |
| GNRH1 | NM_000825 | gonadotropin-releasing hormone 1 (luteinizing-releasing hormone) |
| AURKA | NM_003600 | aurora kinase A |
| VLDLR | NM_001018056 | very low density lipoprotein receptor |
| PTP4A1 | NM_003463 | protein tyrosine phosphatase type IVA, member 1 |
| CLDN10 | NM_001160100 | claudin 10 |
| HRASLS2 | NM_017878 | HRAS-like suppressor 2 |
| ZNF501 | NM_145044 | zinc finger protein 501 |
| GNS | NM_002076 | glucosamine (N-acetyl)-6-sulfatase |
| REEP1 | NM_001164730 | receptor accessory protein 1 |
| FGF13 | NM_001139498 | fibroblast growth factor 13 |
| GAD2 | NM_000818 | glutamate decarboxylase 2 (pancreatic islets and brain, 65kDa) |
| NEU3 | NM_006656 | sialidase 3 (membrane sialidase) |
| RNF13 | NM_007282 | ring finger protein 13 |
| MCTP2 | NM_001159643 | multiple C2 domains, transmembrane 2 |
| RBM26 | NM_022118 | RNA binding motif protein 26 |
| SLC26A3 | NM_000111 | solute carrier family 26, member 3 |
| RPL31 | NM_001098577 | ribosomal protein L31 |
| C16orf45 | NM_001142469 | chromosome 16 open reading frame 45 |
| KIF1B | NM_015074 | kinesin family member 1B |
| COX16 | NM_001204090 | COX16 cytochrome c oxidase assembly homolog (S. cerevisiae) |
| ANKRD36B | NM_025190 | ankyrin repeat domain 36B |
| CHST9 | NM_031422 | carbohydrate (N-acetylgalactosamine 4-0) sulfotransferase 9 |
| SYNJ2BP-COX1( | NM_001202547 | SYNJ2BP-COX16 readthrough |
| ATR | NM_001184 | ataxia telangiectasia and Rad3 related |
| MYCN | NM_005378 | v-myc myelocytomatosis viral related oncogene, neuroblastoma derived (avian) |
| HOMER1 | NM_004272 | homer homolog 1 (Drosophila) |
| PPCDC | NM_021823 | phosphopantothenoylcysteine decarboxylase |
| SH3TC2 | NM_024577 | SH3 domain and tetratricopeptide repeats 2 |
| TFEC | NM_001018058 | transcription factor EC |

| | | |
|---|---|---|
| SLC22A10 | NM_001039752 | solute carrier family 22, member 10 |
| PURG | NM_001015508 | purine-rich element binding protein G |
| CDV3 | NM_001134422 | CDV3 homolog (mouse) |
| RNF145 | NM_001199380 | ring finger protein 145 |
| TRAPPC6B | NM_001079537 | trafficking protein particle complex 6B |
| FBXO45 | NM_001105573 | F-box protein 45 |
| CMPK1 | NM_001136140 | cytidine monophosphate (UMP-CMP) kinase 1, cytosolic |
| RLIM | NM_016120 | ring finger protein, LIM domain interacting |
| AMBRA1 | NM_017749 | autophagy/beclin-1 regulator 1 |
| LRRC31 | NM_024727 | leucine rich repeat containing 31 |
| LBH | NM_030915 | limb bud and heart development homolog (mouse) |
| ZDHHC21 | NM_178566 | zinc finger, DHHC-type containing 21 |
| KCNK2 | NM_001017424 | potassium channel, subfamily K, member 2 |
| PCDHGA11 | NM_032091 | protocadherin gamma subfamily A, 11 |
| TM2D2 | NM_001024380 | TM2 domain containing 2 |
| TTLL2 | NM_031949 | tubulin tyrosine ligase-like family, member 2 |
| C12orf12 | NM_152638 | chromosome 12 open reading frame 12 |
| FBLN2 | NM_001004019 | fibulin 2 |
| PNPLA8 | NM_015723 | patatin-like phospholipase domain containing 8 |
| WDPCP | NM_015910 | WD repeat containing planar cell polarity effector |
| SPATA6 | NM_019073 | spermatogenesis associated 6 |
| THOC7 | NM_025075 | THO complex 7 homolog (Drosophila) |
| MTF2 | NM_001164391 | metal response element binding transcription factor 2 |
| TSEN15 | NM_001127394 | tRNA splicing endonuclease 15 homolog (S. cerevisiae) |
| AIFM1 | NM_001130846 | apoptosis-inducing factor, mitochondrion-associated, 1 |
| BTBD11 | NM_001017523 | BTB (POZ) domain containing 11 |
| GCSH | NM_004483 | glycine cleavage system protein H (aminomethyl carrier) |
| SLC25A24 | NM_013386 | solute carrier family 25 (mitochondrial carrier; phosphate carrier), member 24 |
| C8orf44 | NM_019607 | chromosome 8 open reading frame 44 |
| FAM81A | NM_152450 | family with sequence similarity 81, member A |
| UNC5D | NM_080872 | unc-5 homolog D (C. elegans) |
| CD47 | NM_001777 | CD47 molecule |
| FOXO3 | NM_001455 | forkhead box O3 |
| DUSP16 | NM_030640 | dual specificity phosphatase 16 |
| AVPR1A | NM_000706 | arginine vasopressin receptor 1A |
| FMR1 | NM_001185075 | fragile X mental retardation 1 |
| ZNF200 | NM_001145446 | zinc finger protein 200 |
| ZNF136 | NM_003437 | zinc finger protein 136 |
| PPHLN1 | NM_201438 | periphilin 1 |

| | | |
|---|---|---|
| ERRFI1 | NM_018948 | ERBB receptor feedback inhibitor 1 |
| RAB12 | NM_001025300 | RAB12, member RAS oncogene family |
| KLRD1 | NM_001114396 | killer cell lectin-like receptor subfamily D, member 1 |
| DLC1 | NM_001164271 | deleted in liver cancer 1 |
| NANOS1 | NM_199461 | nanos homolog 1 (Drosophila) |
| PKHD1 | NM_138694 | polycystic kidney and hepatic disease 1 (autosomal recessive) |
| TRIM9 | NM_052978 | tripartite motif containing 9 |
| C6orf192 | NM_052831 | chromosome 6 open reading frame 192 |
| GPR180 | NM_180989 | G protein-coupled receptor 180 |
| TMED5 | NM_001167830 | transmembrane emp24 protein transport domain containing 5 |
| ERC2 | NM_015576 | ELKS/RAB6-interacting/CAST family member 2 |
| INSIG1 | NM_005542 | insulin induced gene 1 |
| FEZ2 | NM_001042548 | fasciculation and elongation protein zeta 2 (zygin II) |
| GPR171 | NM_013308 | G protein-coupled receptor 171 |
| CDC37L1 | NM_017913 | cell division cycle 37 homolog (S. cerevisiae)-like 1 |
| RANBP3L | NM_001161429 | RAN binding protein 3-like |
| HTR7 | NM_000872 | 5-hydroxytryptamine (serotonin) receptor 7 (adenylate cyclase-coupled) |
| ZNF19 | NM_006961 | zinc finger protein 19 |
| ALG10 | NM_032834 | asparagine-linked glycosylation 10, alpha-1,2-glucosyltransferase homolog (S. pombe) |
| GPX8 | NM_001008397 | glutathione peroxidase 8 (putative) |
| TGIF1 | NM_003244 | TGFB-induced factor homeobox 1 |
| KDM4A | NM_014663 | lysine (K)-specific demethylase 4A |
| STXBP5 | NM_001127715 | syntaxin binding protein 5 (tomosyn) |
| AGA | NM_000027 | aspartylglucosaminidase |
| NPY2R | NM_000910 | neuropeptide Y receptor Y2 |
| TCEB3 | NM_003198 | transcription elongation factor B (SIII), polypeptide 3 (110kDa, elongin A) |
| CCNL2 | NM_001039577 | cyclin L2 |
| OLR1 | NM_001172632 | oxidized low density lipoprotein (lectin-like) receptor 1 |
| ZFPM2 | NM_012082 | zinc finger protein, multitype 2 |
| E2F3 | NM_001949 | E2F transcription factor 3 |
| SMAD7 | NM_001190821 | SMAD family member 7 |
| PRKCE | NM_005400 | protein kinase C, epsilon |
| ESM1 | NM_001135604 | endothelial cell-specific molecule 1 |
| PRRC2C | NM_015172 | proline-rich coiled-coil 2C |
| CD33 | NM_001082618 | CD33 molecule |
| EPB41L2 | NM_001135554 | erythrocyte membrane protein band 4.1-like 2 |
| ZNF20 | NM_001203250 | zinc finger protein 20 |
| CAMKK2 | NM_172214 | calcium/calmodulin-dependent protein kinase kinase 2, beta |
| ISCU | NM_014301 | iron-sulfur cluster scaffold homolog (E. coli) |

| | | |
|---|---|---|
| TMEM87B | NM_032824 | transmembrane protein 87B |
| COL12A1 | NM_004370 | collagen, type XII, alpha 1 |
| RNF138 | NM_001191324 | ring finger protein 138 |
| C14orf184 | NM_001080113 | chromosome 14 open reading frame 184 |
| VCAN | NM_001126336 | versican |
| DAPK1 | NM_004938 | death-associated protein kinase 1 |
| LDLR | NM_000527 | low density lipoprotein receptor |
| RPS6KB1 | NM_003161 | ribosomal protein S6 kinase, 70kDa, polypeptide 1 |
| ABCA8 | NM_007168 | ATP-binding cassette, sub-family A (ABC1), member 8 |
| AARSD1 | NM_001142653 | alanyl-tRNA synthetase domain containing 1 |
| LOC100293516 | NM_001204818 | zinc finger protein |
| PPIP5K2 | NM_015216 | diphosphoinositol pentakisphosphate kinase 2 |
| TGDS | NM_014305 | TDP-glucose 4,6-dehydratase |
| EXOC8 | NM_175876 | exocyst complex component 8 |
| AZIN1 | NM_015878 | antizyme inhibitor 1 |
| PLSCR3 | NM_001201576 | phospholipid scramblase 3 |
| MMP25 | NM_022468 | matrix metallopeptidase 25 |
| ZNF519 | NM_145287 | zinc finger protein 519 |
| SLC22A3 | NM_021977 | solute carrier family 22 (extraneuronal monoamine transporter), member 3 |
| SLC25A30 | NM_001010875 | solute carrier family 25, member 30 |
| CCDC144NL | NM_001004306 | coiled-coil domain containing 144 family, N-terminal like |
| SOX9 | NM_000346 | SRY (sex determining region Y)-box 9 |
| ATG14 | NM_014924 | ATG14 autophagy related 14 homolog (S. cerevisiae) |
| COBL | NM_015198 | cordon-bleu homolog (mouse) |
| C4orf41 | NM_021942 | chromosome 4 open reading frame 41 |
| ZNF432 | NM_014650 | zinc finger protein 432 |
| CUTC | NM_015960 | cutC copper transporter homolog (E. coli) |
| NCAPG2 | NM_017760 | non-SMC condensin II complex, subunit G2 |
| ARL6 | NM_032146 | ADP-ribosylation factor-like 6 |
| ZNF396 | NM_145756 | zinc finger protein 396 |
| IKZF2 | NM_001079526 | IKAROS family zinc finger 2 (Helios) |
| CEACAM5 | NM_004363 | carcinoembryonic antigen-related cell adhesion molecule 5 |
| USP34 | NM_014709 | ubiquitin specific peptidase 34 |
| PHF21B | NM_001135862 | PHD finger protein 21B |
| TGM5 | NM_004245 | transglutaminase 5 |
| P2RY12 | NM_022788 | purinergic receptor P2Y, G-protein coupled, 12 |
| AMICA1 | NM_001098526 | adhesion molecule, interacts with CXADR antigen 1 |
| SPRED1 | NM_152594 | sprouty-related, EVH1 domain containing 1 |
| GNB1 | NM_002074 | guanine nucleotide binding protein (G protein), beta polypeptide 1 |

| | | |
|---|---|---|
| TSPAN12 | NM_012338 | tetraspanin 12 |
| C5orf22 | NM_018356 | chromosome 5 open reading frame 22 |
| AGBL4 | NM_032785 | ATP/GTP binding protein-like 4 |
| MMP13 | NM_002427 | matrix metallopeptidase 13 (collagenase 3) |
| ZNF789 | NM_001013258 | zinc finger protein 789 |
| LOC646851 | NM_001013647 | hypothetical LOC646851 |
| BTBD7 | NM_001002860 | BTB (POZ) domain containing 7 |
| GABRG1 | NM_173536 | gamma-aminobutyric acid (GABA) A receptor, gamma 1 |
| HNMT | NM_006895 | histamine N-methyltransferase |
| SRPR | NM_001177842 | signal recognition particle receptor (docking protein) |
| FMN2 | NM_020066 | formin 2 |
| SCRT2 | NM_033129 | scratch homolog 2, zinc finger protein (Drosophila) |
| CCL11 | NM_002986 | chemokine (C-C motif) ligand 11 |
| ZNF14 | NM_021030 | zinc finger protein 14 |
| PLK2 | NM_006622 | polo-like kinase 2 |
| ZNF623 | NM_001082480 | zinc finger protein 623 |
| DNAH5 | NM_001369 | dynein, axonemal, heavy chain 5 |
| TM4SF1 | NM_014220 | transmembrane 4 L six family member 1 |
| RAD1 | NM_002853 | RAD1 homolog (S. pombe) |
| SORBS2 | NM_001145670 | sorbin and SH3 domain containing 2 |
| PAK4 | NM_001014831 | p21 protein (Cdc42/Rac)-activated kinase 4 |
| EIF2C2 | NM_001164623 | eukaryotic translation initiation factor 2C, 2 |
| LPP | NM_001167671 | LIM domain containing preferred translocation partner in lipoma |
| C16orf46 | NM_001100873 | chromosome 16 open reading frame 46 |
| ELMOD2 | NM_153702 | ELMO/CED-12 domain containing 2 |
| UBP1 | NM_001128160 | upstream binding protein 1 (LBP-1a) |
| NCAPD3 | NM_015261 | non-SMC condensin II complex, subunit D3 |
| NBR1 | NM_005899 | neighbor of BRCA1 gene 1 |
| RNF4 | NM_001185009 | ring finger protein 4 |
| C4orf32 | NM_152400 | chromosome 4 open reading frame 32 |
| C7orf71 | NM_001145531 | chromosome 7 open reading frame 71 |
| TRAF6 | NM_004620 | TNF receptor-associated factor 6 |
| RBM41 | NM_001171080 | RNA binding motif protein 41 |
| PAX3 | NM_000438 | paired box 3 |
| DGKI | NM_004717 | diacylglycerol kinase, iota |
| APBA3 | NM_004886 | amyloid beta (A4) precursor protein-binding, family A, member 3 |
| MAK | NM_001242385 | male germ cell-associated kinase |
| NRIP1 | NM_003489 | nuclear receptor interacting protein 1 |
| B3GNT1 | NM_006876 | UDP-GlcNAc:betaGal beta-1,3-N-acetylglucosaminyltransferase 1 |

| | | |
|---|---|---|
| DNAJB4 | NM_007034 | DnaJ (Hsp40) homolog, subfamily B, member 4 |
| C10orf93 | NM_173572 | chromosome 10 open reading frame 93 |
| FBXO25 | NM_012173 | F-box protein 25 |
| SLC15A2 | NM_001145998 | solute carrier family 15 (H+/peptide transporter), member 2 |
| APPL1 | NM_012096 | adaptor protein, phosphotyrosine interaction, PH domain and leucine zipper containing |
| SERBP1 | NM_001018067 | SERPINE1 mRNA binding protein 1 |
| DPPA4 | NM_018189 | developmental pluripotency associated 4 |
| ACBD5 | NM_001042473 | acyl-CoA binding domain containing 5 |
| RTP4 | NM_022147 | receptor (chemosensory) transporter protein 4 |
| LSAMP | NM_002338 | limbic system-associated membrane protein |
| HLA-DRB1 | NM_002124 | major histocompatibility complex, class II, DR beta 1 |
| TRIP6 | NM_003302 | thyroid hormone receptor interactor 6 |
| YES1 | NM_005433 | v-yes-1 Yamaguchi sarcoma viral oncogene homolog 1 |
| ZNF831 | NM_178457 | zinc finger protein 831 |
| MTRR | NM_002454 | 5-methyltetrahydrofolate-homocysteine methyltransferase reductase |
| SDC2 | NM_002998 | syndecan 2 |
| MORC3 | NM_015358 | MORC family CW-type zinc finger 3 |
| ATAD2 | NM_014109 | ATPase family, AAA domain containing 2 |
| CCDC83 | NM_173556 | coiled-coil domain containing 83 |
| LRP8 | NM_001018054 | low density lipoprotein receptor-related protein 8, apolipoprotein e receptor |
| CADM2 | NM_001167674 | cell adhesion molecule 2 |
| ZKSCAN1 | NM_003439 | zinc finger with KRAB and SCAN domains 1 |
| PDS5A | NM_001100399 | PDS5, regulator of cohesion maintenance, homolog A (S. cerevisiae) |
| CREBZF | NM_001039618 | CREB/ATF bZIP transcription factor |
| C13orf18 | NM_025113 | chromosome 13 open reading frame 18 |
| DNAH12 | NM_198564 | dynein, axonemal, heavy chain 12 |
| TMSB4Y | NM_004202 | thymosin beta 4, Y-linked |
| TNIP3 | NM_001128843 | TNFAIP3 interacting protein 3 |
| STX3 | NM_001178040 | syntaxin 3 |
| CBR4 | NM_032783 | carbonyl reductase 4 |
| CHRNA5 | NM_000745 | cholinergic receptor, nicotinic, alpha 5 |
| CLDN11 | NM_001185056 | claudin 11 |
| HTR2C | NM_000868 | 5-hydroxytryptamine (serotonin) receptor 2C |
| PACS2 | NM_001100913 | phosphofurin acidic cluster sorting protein 2 |
| OSBPL8 | NM_001003712 | oxysterol binding protein-like 8 |
| LGALS8 | NM_006499 | lectin, galactoside-binding, soluble, 8 |
| EIF4E | NM_001130678 | eukaryotic translation initiation factor 4E |
| CAMK2G | NM_001204492 | calcium/calmodulin-dependent protein kinase II gamma |
| COL19A1 | NM_001858 | collagen, type XIX, alpha 1 |

| | | |
|---|---|---|
| PCSK6 | NM_002570 | proprotein convertase subtilisin/kexin type 6 |
| TACR1 | NM_001058 | tachykinin receptor 1 |
| KAT7 | NM_001199155 | K(lysine) acetyltransferase 7 |
| HAUS3 | NM_024511 | HAUS augmin-like complex, subunit 3 |
| ASRGL1 | NM_001083926 | asparaginase like 1 |
| C3orf26 | NM_001167924 | chromosome 3 open reading frame 26 |
| NDP | NM_000266 | Norrie disease (pseudoglioma) |
| UFM1 | NM_016617 | ubiquitin-fold modifier 1 |
| C1orf94 | NM_001134734 | chromosome 1 open reading frame 94 |
| EIF4E3 | NM_001134649 | eukaryotic translation initiation factor 4E family member 3 |
| WDR48 | NM_020839 | WD repeat domain 48 |
| ZNF143 | NM_003442 | zinc finger protein 143 |
| SULF2 | NM_001161841 | sulfatase 2 |
| RSPH4A | NM_001010892 | radial spoke head 4 homolog A (Chlamydomonas) |
| INADL | NM_176877 | InaD-like (Drosophila) |
| TACSTD2 | NM_002353 | tumor-associated calcium signal transducer 2 |
| TOX3 | NM_001080430 | TOX high mobility group box family member 3 |
| PIGX | NM_001166304 | phosphatidylinositol glycan anchor biosynthesis, class X |
| TTC31 | NM_022492 | tetratricopeptide repeat domain 31 |
| IL10RA | NM_001558 | interleukin 10 receptor, alpha |
| CYTIP | NM_004288 | cytohesin 1 interacting protein |
| SPCS1 | NM_014041 | signal peptidase complex subunit 1 homolog (S. cerevisiae) |
| C9orf125 | NM_032342 | chromosome 9 open reading frame 125 |
| NFATC2IP | NM_032815 | nuclear factor of activated T-cells, cytoplasmic, calcineurin-dependent 2 interacting |
| TGS1 | NM_024831 | trimethylguanosine synthase 1 |
| TAB3 | NM_152787 | TGF-beta activated kinase 1/MAP3K7 binding protein 3 |
| PDK3 | NM_001142386 | pyruvate dehydrogenase kinase, isozyme 3 |
| EPN2 | NM_001102664 | epsin 2 |
| NOS2 | NM_000625 | nitric oxide synthase 2, inducible |
| SMG7 | NM_001174061 | smg-7 homolog, nonsense mediated mRNA decay factor (C. elegans) |
| ZDHHC17 | NM_015336 | zinc finger, DHHC-type containing 17 |
| AP3S1 | NM_001284 | adaptor-related protein complex 3, sigma 1 subunit |
| DLG1 | NM_001098424 | discs, large homolog 1 (Drosophila) |
| ZNF292 | NM_015021 | zinc finger protein 292 |
| NUP62CL | NM_017681 | nucleoporin 62kDa C-terminal like |
| C7orf69 | NM_025031 | chromosome 7 open reading frame 69 |
| SYT9 | NM_175733 | synaptotagmin IX |
| SFXN1 | NM_022754 | sideroflexin 1 |
| MOCS1 | NM_001075098 | molybdenum cofactor synthesis 1 |

| | | |
|---|---|---|
| IL1R1 | NM_000877 | interleukin 1 receptor, type I |
| NTRK2 | NM_001018065 | neurotrophic tyrosine kinase, receptor, type 2 |
| IRS2 | NM_003749 | insulin receptor substrate 2 |
| SART3 | NM_014706 | squamous cell carcinoma antigen recognized by T cells 3 |
| KCNK15 | NM_022358 | potassium channel, subfamily K, member 15 |
| TMEM123 | NM_052932 | transmembrane protein 123 |
| RPS6KA5 | NM_004755 | ribosomal protein S6 kinase, 90kDa, polypeptide 5 |
| KLHL7 | NM_001031710 | kelch-like 7 (Drosophila) |
| ATP11C | NM_001010986 | ATPase, class VI, type 11C |
| RGSL1 | NM_001137669 | regulator of G-protein signaling like 1 |
| PYROXD1 | NM_024854 | pyridine nucleotide-disulphide oxidoreductase domain 1 |
| SIK3 | NM_025164 | SIK family kinase 3 |
| ANP32E | NM_001136478 | acidic (leucine-rich) nuclear phosphoprotein 32 family, member E |
| POLE | NM_006231 | polymerase (DNA directed), epsilon |
| SAG | NM_000541 | S-antigen; retina and pineal gland (arrestin) |
| TAF1B | NM_005680 | TATA box binding protein (TBP)-associated factor, RNA polymerase I, B, 63kDa |
| TTLL5 | NM_015072 | tubulin tyrosine ligase-like family, member 5 |
| MCCC2 | NM_022132 | methylcrotonoyl-CoA carboxylase 2 (beta) |
| INPP4A | NM_001134224 | inositol polyphosphate-4-phosphatase, type I, 107kDa |
| NR4A2 | NM_006186 | nuclear receptor subfamily 4, group A, member 2 |
| DCUN1D4 | NM_001040402 | DCN1, defective in cullin neddylation 1, domain containing 4 (S. cerevisiae) |
| PCDHB7 | NM_018940 | protocadherin beta 7 |
| ABHD6 | NM_020676 | abhydrolase domain containing 6 |
| BCAN | NM_021948 | brevican |
| ESRP2 | NM_024939 | epithelial splicing regulatory protein 2 |
| VCL | NM_003373 | vinculin |
| UBR2 | NM_001184801 | ubiquitin protein ligase E3 component n-recognin 2 |
| CAND1 | NM_018448 | cullin-associated and neddylation-dissociated 1 |
| MTRNR2L7 | NM_001190489 | MT-RNR2-like 7 |
| TP53INP2 | NM_021202 | tumor protein p53 inducible nuclear protein 2 |
| POTEF | NM_001099771 | POTE ankyrin domain family, member F |
| SLC11A1 | NM_000578 | solute carrier family 11 (proton-coupled divalent metal ion transporters), member 1 |
| SLC34A2 | NM_001177998 | solute carrier family 34 (sodium phosphate), member 2 |
| LONP2 | NM_031490 | lon peptidase 2, peroxisomal |
| FAS | NM_000043 | Fas (TNF receptor superfamily, member 6) |
| CCNE2 | NM_057749 | cyclin E2 |
| MCPH1 | NM_001172574 | microcephalin 1 |
| SYTL5 | NM_001163334 | synaptotagmin-like 5 |
| FAM55C | NM_001134456 | family with sequence similarity 55, member C |

| | | |
|---|---|---|
| CRAT | NM_000755 | carnitine O-acetyltransferase |
| SNX18 | NM_001102575 | sorting nexin 18 |
| APOA4 | NM_000482 | apolipoprotein A-IV |
| KIAA1147 | NM_001080392 | KIAA1147 |
| GABRG2 | NM_000816 | gamma-aminobutyric acid (GABA) A receptor, gamma 2 |
| SPRY3 | NM_005840 | sprouty homolog 3 (Drosophila) |
| SLC39A6 | NM_012319 | solute carrier family 39 (zinc transporter), member 6 |
| SP140L | NM_138402 | SP140 nuclear body protein-like |
| MESDC1 | NM_022566 | mesoderm development candidate 1 |
| SLC44A5 | NM_001130058 | solute carrier family 44, member 5 |
| ALDH1B1 | NM_000692 | aldehyde dehydrogenase 1 family, member B1 |
| SOX11 | NM_003108 | SRY (sex determining region Y)-box 11 |
| TRPA1 | NM_007332 | transient receptor potential cation channel, subfamily A, member 1 |
| KIAA1033 | NM_015275 | KIAA1033 |
| XRRA1 | NM_182969 | X-ray radiation resistance associated 1 |
| NOTCH2NL | NM_203458 | notch 2 N-terminal like |
| ZNF576 | NM_001145347 | zinc finger protein 576 |
| KCTD10 | NM_031954 | potassium channel tetramerisation domain containing 10 |
| GPR146 | NM_138445 | G protein-coupled receptor 146 |
| B3GALTL | NM_194318 | beta 1,3-galactosyltransferase-like |
| KCNJ11 | NM_000525 | potassium inwardly-rectifying channel, subfamily J, member 11 |
| SCAF11 | NM_004719 | SR-related CTD-associated factor 11 |
| PDCD4 | NM_001199492 | programmed cell death 4 (neoplastic transformation inhibitor) |
| FAM178A | NM_018121 | family with sequence similarity 178, member A |
| AZI2 | NM_022461 | 5-azacytidine induced 2 |
| KDELC1 | NM_024089 | KDEL (Lys-Asp-Glu-Leu) containing 1 |
| ARHGAP12 | NM_018287 | Rho GTPase activating protein 12 |
| RNF148 | NM_198085 | ring finger protein 148 |
| MGAT4A | NM_012214 | mannosyl (alpha-1,3-)-glycoprotein beta-1,4-N-acetylglucosaminyltransferase, |
| CDK6 | NM_001145306 | cyclin-dependent kinase 6 |
| PDSS2 | NM_020381 | prenyl (decaprenyl) diphosphate synthase, subunit 2 |
| PSIP1 | NM_001128217 | PC4 and SFRS1 interacting protein 1 |
| C1orf88 | NM_181643 | chromosome 1 open reading frame 88 |
| LOC100130890 | NM_001195131 | hypothetical protein LOC100130890 |
| RYR3 | NM_001036 | ryanodine receptor 3 |
| RBM47 | NM_001098634 | RNA binding motif protein 47 |
| CHD7 | NM_017780 | chromodomain helicase DNA binding protein 7 |
| CTAGE1 | NM_172241 | cutaneous T-cell lymphoma-associated antigen 1 |
| AGPAT9 | NM_032717 | 1-acylglycerol-3-phosphate O-acyltransferase 9 |

| | | |
|---|---|---|
| ZNF92 | NM_007139 | zinc finger protein 92 |
| FAM190A | NM_207491 | family with sequence similarity 190, member A |
| ATXN3 | NM_001127696 | ataxin 3 |
| MBNL3 | NM_001170701 | muscleblind-like 3 (Drosophila) |
| PDZK1 | NM_001201325 | PDZ domain containing 1 |
| IMPACT | NM_018439 | Impact homolog (mouse) |
| KCNH8 | NM_144633 | potassium voltage-gated channel, subfamily H (eag-related), member 8 |
| ASB14 | NM_001142733 | ankyrin repeat and SOCS box containing 14 |
| OR52B4 | NM_001005161 | olfactory receptor, family 52, subfamily B, member 4 |
| RAB11FIP1 | NM_001002814 | RAB11 family interacting protein 1 (class I) |
| SOX1 | NM_005986 | SRY (sex determining region Y)-box 1 |
| BEND4 | NM_001159547 | BEN domain containing 4 |
| ZBED2 | NM_024508 | zinc finger, BED-type containing 2 |
| SYNC | NM_001161708 | syncoilin, intermediate filament protein |
| RUNX2 | NM_001015051 | runt-related transcription factor 2 |
| KIAA1383 | NM_019090 | KIAA1383 |
| MAGI1 | NM_001033057 | membrane associated guanylate kinase, WW and PDZ domain containing 1 |
| C15orf24 | NM_020154 | chromosome 15 open reading frame 24 |
| CCDC89 | NM_152723 | coiled-coil domain containing 89 |
| SMCHD1 | NM_015295 | structural maintenance of chromosomes flexible hinge domain containing 1 |
| CLEC4E | NM_014358 | C-type lectin domain family 4, member E |
| AACS | NM_023928 | acetoacetyl-CoA synthetase |
| ZNF680 | NM_178558 | zinc finger protein 680 |
| FRMD6 | NM_001042481 | FERM domain containing 6 |
| PTAR1 | NM_001099666 | protein prenyltransferase alpha subunit repeat containing 1 |
| C10orf76 | NM_024541 | chromosome 10 open reading frame 76 |
| BMPR1B | NM_001203 | bone morphogenetic protein receptor, type IB |
| KCNA7 | NM_031886 | potassium voltage-gated channel, shaker-related subfamily, member 7 |
| CDK17 | NM_001170464 | cyclin-dependent kinase 17 |
| CCDC88A | NM_001135597 | coiled-coil domain containing 88A |
| RERGL | NM_024730 | RERG/RAS-like |
| MTRNR2L5 | NM_001190478 | MT-RNR2-like 5 |
| EMR2 | NM_013447 | egf-like module containing, mucin-like, hormone receptor-like 2 |
| ASB1 | NM_001040445 | ankyrin repeat and SOCS box containing 1 |
| NR1I2 | NM_003889 | nuclear receptor subfamily 1, group I, member 2 |
| MRPS27 | NM_015084 | mitochondrial ribosomal protein S27 |
| TTC18 | NM_145170 | tetratricopeptide repeat domain 18 |
| FAM19A4 | NM_001005527 | family with sequence similarity 19 (chemokine (C-C motif)-like), member A4 |
| MID2 | NM_012216 | midline 2 |

| | | |
|---|---|---|
| TCF7L2 | NM_001146274 | transcription factor 7-like 2 (T-cell specific, HMG-box) |
| KCNC1 | NM_001112741 | potassium voltage-gated channel, Shaw-related subfamily, member 1 |
| LPGAT1 | NM_014873 | lysophosphatidylglycerol acyltransferase 1 |
| RNF220 | NM_018150 | ring finger protein 220 |
| ALDH3A2 | NM_000382 | aldehyde dehydrogenase 3 family, member A2 |
| BNIP3L | NM_004331 | BCL2/adenovirus E1B 19kDa interacting protein 3-like |
| HMG20A | NM_018200 | high mobility group 20A |
| SERP1 | NM_014445 | stress-associated endoplasmic reticulum protein 1 |
| C20orf12 | NM_001099407 | chromosome 20 open reading frame 12 |
| SLTM | NM_001013843 | SAFB-like, transcription modulator |
| TOMM5 | NM_001001790 | translocase of outer mitochondrial membrane 5 homolog (yeast) |
| MTRNR2L10 | NM_001190708 | MT-RNR2-like 10 |
| ZRANB1 | NM_017580 | zinc finger, RAN-binding domain containing 1 |
| ZFY | NM_001145275 | zinc finger protein, Y-linked |
| RPRD2 | NM_015203 | regulation of nuclear pre-mRNA domain containing 2 |
| TNKS2 | NM_025235 | tankyrase, TRF1-interacting ankyrin-related ADP-ribose polymerase 2 |
| ANKRD45 | NM_198493 | ankyrin repeat domain 45 |
| APLNR | NM_005161 | apelin receptor |
| IL4R | NM_000418 | interleukin 4 receptor |
| MAN2A1 | NM_002372 | mannosidase, alpha, class 2A, member 1 |
| PLAT | NM_000930 | plasminogen activator, tissue |
| PPP1R2 | NM_006241 | protein phosphatase 1, regulatory (inhibitor) subunit 2 |
| MAP7 | NM_001198608 | microtubule-associated protein 7 |
| ZNF382 | NM_032825 | zinc finger protein 382 |
| FAM122A | NM_138333 | family with sequence similarity 122A |
| TMEM120B | NM_001080825 | transmembrane protein 120B |
| ELAVL1 | NM_001419 | ELAV (embryonic lethal, abnormal vision, Drosophila)-like 1 (Hu antigen R) |
| SEMA3A | NM_006080 | sema domain, immunoglobulin domain (Ig), short basic domain, secreted, |
| CASC1 | NM_001082972 | cancer susceptibility candidate 1 |
| PLEKHA3 | NM_019091 | pleckstrin homology domain containing, family A (phosphoinositide binding specific) |
| RASGEF1A | NM_145313 | RasGEF domain family, member 1A |
| SLCO4C1 | NM_180991 | solute carrier organic anion transporter family, member 4C1 |
| C16orf87 | NM_001001436 | chromosome 16 open reading frame 87 |
| SNPH | NM_014723 | syntaphilin |
| ZNF25 | NM_145011 | zinc finger protein 25 |
| OBFC2A | NM_001031716 | oligonucleotide/oligosaccharide-binding fold containing 2A |
| ACTC1 | NM_005159 | actin, alpha, cardiac muscle 1 |
| KLF9 | NM_001206 | Kruppel-like factor 9 |
| SRD5A1 | NM_001047 | steroid-5-alpha-reductase, alpha polypeptide 1 (3-oxo-5 alpha-steroid delta 4- |

| | | |
|---|---|---|
| RAB11A | NM_001206836 | RAB11A, member RAS oncogene family |
| ADAMTS4 | NM_005099 | ADAM metallopeptidase with thrombospondin type 1 motif, 4 |
| RGAG1 | NM_020769 | retrotransposon gag domain containing 1 |
| C2orf3 | NM_001201334 | chromosome 2 open reading frame 3 |
| B3GNT2 | NM_006577 | UDP-GlcNAc:betaGal beta-1,3-N-acetylglucosaminyltransferase 2 |
| KLF8 | NM_001159296 | Kruppel-like factor 8 |
| TMEM56-RWDD: | NM_001199691 | TMEM56-RWDD3 readthrough |
| TRIB2 | NM_021643 | tribbles homolog 2 (Drosophila) |
| C1orf198 | NM_001136494 | chromosome 1 open reading frame 198 |
| LNX2 | NM_153371 | ligand of numb-protein X 2 |
| PLCH1 | NM_001130960 | phospholipase C, eta 1 |
| ODF2L | NM_001184765 | outer dense fiber of sperm tails 2-like |
| SMURF2 | NM_022739 | SMAD specific E3 ubiquitin protein ligase 2 |
| ZNF439 | NM_152262 | zinc finger protein 439 |
| WNK3 | NM_001002838 | WNK lysine deficient protein kinase 3 |
| KIAA0319 | NM_001168374 | KIAA0319 |
| SIGMAR1 | NM_005866 | sigma non-opioid intracellular receptor 1 |
| ZNF346 | NM_012279 | zinc finger protein 346 |
| PAFAH1B1 | NM_000430 | platelet-activating factor acetylhydrolase 1b, regulatory subunit 1 (45kDa) |
| TDG | NM_003211 | thymine-DNA glycosylase |
| JHDM1D | NM_030647 | jumonji C domain containing histone demethylase 1 homolog D (S. cerevisiae) |
| NTRK3 | NM_001007156 | neurotrophic tyrosine kinase, receptor, type 3 |
| SSPN | NM_001135823 | sarcospan (Kras oncogene-associated gene) |
| RPRD1B | NM_021215 | regulation of nuclear pre-mRNA domain containing 1B |
| ITGA2 | NM_002203 | integrin, alpha 2 (CD49B, alpha 2 subunit of VLA-2 receptor) |
| RANBP2 | NM_006267 | RAN binding protein 2 |
| CROT | NM_001143935 | carnitine O-octanoyltransferase |
| MIS12 | NM_024039 | MIS12, MIND kinetochore complex component, homolog (S. pombe) |
| PRKD3 | NM_005813 | protein kinase D3 |
| SLC6A20 | NM_020208 | solute carrier family 6 (proline IMINO transporter), member 20 |
| ARMCX3 | NM_016607 | armadillo repeat containing, X-linked 3 |
| USP36 | NM_025090 | ubiquitin specific peptidase 36 |
| TTC26 | NM_001144920 | tetratricopeptide repeat domain 26 |
| DDHD1 | NM_001160147 | DDHD domain containing 1 |
| DCAF4L1 | NM_001029955 | DDB1 and CUL4 associated factor 4-like 1 |
| VAMP7 | NM_001145149 | vesicle-associated membrane protein 7 |
| TSPAN5 | NM_005723 | tetraspanin 5 |
| MTHFD2L | NM_001144978 | methylenetetrahydrofolate dehydrogenase (NADP+ dependent) 2-like |
| HLA-DOA | NM_002119 | major histocompatibility complex, class II, DO alpha |

| | | |
|---|---|---|
| IPCEF1 | NM_001130699 | interaction protein for cytohesin exchange factors 1 |
| FASLG | NM_000639 | Fas ligand (TNF superfamily, member 6) |
| DNASE2 | NM_001375 | deoxyribonuclease II, lysosomal |
| TCF21 | NM_198392 | transcription factor 21 |
| DCAF6 | NM_001017977 | DDB1 and CUL4 associated factor 6 |
| KLHDC8B | NM_173546 | kelch domain containing 8B |
| ZNF575 | NM_174945 | zinc finger protein 575 |
| CD28 | NM_006139 | CD28 molecule |
| CR2 | NM_001006658 | complement component (3d/Epstein Barr virus) receptor 2 |
| CNOT4 | NM_001008225 | CCR4-NOT transcription complex, subunit 4 |
| CXorf1 | NM_004709 | chromosome X open reading frame 1 |
| HIF3A | NM_152796 | hypoxia inducible factor 3, alpha subunit |
| AKD1 | NM_145025 | adenylate kinase domain containing 1 |
| VAPA | NM_003574 | VAMP (vesicle-associated membrane protein)-associated protein A, 33kDa |
| GOLGA7B | NM_001010917 | golgin A7 family, member B |
| RORA | NM_002943 | RAR-related orphan receptor A |
| ZDHHC3 | NM_001135179 | zinc finger, DHHC-type containing 3 |
| SSH1 | NM_018984 | slingshot homolog 1 (Drosophila) |
| SESTD1 | NM_178123 | SEC14 and spectrin domains 1 |
| PLXNA4 | NM_001105543 | plexin A4 |
| TCF7 | NM_001134851 | transcription factor 7 (T-cell specific, HMG-box) |
| SLC16A7 | NM_004731 | solute carrier family 16, member 7 (monocarboxylic acid transporter 2) |
| ICK | NM_014920 | intestinal cell (MAK-like) kinase |
| LMO3 | NM_001001395 | LIM domain only 3 (rhombotin-like 2) |
| PARP1 | NM_001618 | poly (ADP-ribose) polymerase 1 |
| SRSF6 | NM_006275 | serine/arginine-rich splicing factor 6 |
| YKT6 | NM_006555 | YKT6 v-SNARE homolog (S. cerevisiae) |
| WDHD1 | NM_001008396 | WD repeat and HMG-box DNA binding protein 1 |
| ZNF677 | NM_182609 | zinc finger protein 677 |
| UNC13C | NM_001080534 | unc-13 homolog C (C. elegans) |
| DCC | NM_005215 | deleted in colorectal carcinoma |
| SNTB1 | NM_021021 | syntrophin, beta 1 (dystrophin-associated protein A1, 59kDa, basic component 1) |
| MEF2A | NM_001130926 | myocyte enhancer factor 2A |
| AFTPH | NM_001002243 | aftiphilin |
| JUB | NM_032876 | jub, ajuba homolog (Xenopus laevis) |
| SDPR | NM_004657 | serum deprivation response |
| SEPT11 | NM_018243 | septin 11 |
| POLR1E | NM_022490 | polymerase (RNA) I polypeptide E, 53kDa |
| VEPH1 | NM_001167915 | ventricular zone expressed PH domain homolog 1 (zebrafish) |

| | | |
|---|---|---|
| MPRIP | NM_015134 | myosin phosphatase Rho interacting protein |
| ANKRD33B | NM_001164440 | ankyrin repeat domain 33B |
| CALB2 | NM_001740 | calbindin 2 |
| EDN3 | NM_000114 | endothelin 3 |
| JAK3 | NM_000215 | Janus kinase 3 |
| U2SURP | NM_001080415 | U2 snRNP-associated SURP domain containing |
| MKX | NM_001242702 | mohawk homeobox |
| LHFPL3 | NM_199000 | lipoma HMGIC fusion partner-like 3 |
| ANTXR1 | NM_032208 | anthrax toxin receptor 1 |
| C9orf170 | NM_001001709 | chromosome 9 open reading frame 170 |
| SERPINB9 | NM_004155 | serpin peptidase inhibitor, clade B (ovalbumin), member 9 |
| GAGE1 | NM_001040663 | G antigen 1 |
| ABCB10 | NM_012089 | ATP-binding cassette, sub-family B (MDR/TAP), member 10 |
| PGAP2 | NM_001145438 | post-GPI attachment to proteins 2 |
| IPO9 | NM_018085 | importin 9 |
| MRM1 | NM_024864 | mitochondrial rRNA methyltransferase 1 homolog (S. cerevisiae) |
| C9orf3 | NM_001193329 | chromosome 9 open reading frame 3 |
| ZNF569 | NM_152484 | zinc finger protein 569 |
| ITGAV | NM_001144999 | integrin, alpha V (vitronectin receptor, alpha polypeptide, antigen CD51) |
| LCP1 | NM_002298 | lymphocyte cytosolic protein 1 (L-plastin) |
| ZNF26 | NM_019591 | zinc finger protein 26 |
| STK17B | NM_004226 | serine/threonine kinase 17b |
| PNRC2 | NM_017761 | proline-rich nuclear receptor coactivator 2 |
| RIMKLB | NM_020734 | ribosomal modification protein rimK-like family member B |
| SLC7A14 | NM_020949 | solute carrier family 7 (orphan transporter), member 14 |
| THAP2 | NM_031435 | THAP domain containing, apoptosis associated protein 2 |
| NAIF1 | NM_197956 | nuclear apoptosis inducing factor 1 |
| RASSF10 | NM_001080521 | Ras association (RalGDS/AF-6) domain family (N-terminal) member 10 |
| QKI | NM_006775 | quaking homolog, KH domain RNA binding (mouse) |
| PHF17 | NM_024900 | PHD finger protein 17 |
| C1orf52 | NM_198077 | chromosome 1 open reading frame 52 |
| C1orf9 | NM_014283 | chromosome 1 open reading frame 9 |
| CNDP2 | NM_001168499 | CNDP dipeptidase 2 (metallopeptidase M20 family) |
| TPD52L3 | NM_033516 | tumor protein D52-like 3 |
| PLEKHB2 | NM_001100623 | pleckstrin homology domain containing, family B (evectins) member 2 |
| RNF213 | NM_020914 | ring finger protein 213 |
| EDA | NM_001005609 | ectodysplasin A |
| IRAK1 | NM_001025242 | interleukin-1 receptor-associated kinase 1 |
| NDUFB10 | NM_004548 | NADH dehydrogenase (ubiquinone) 1 beta subcomplex, 10, 22kDa |

| | | |
|---|---|---|
| TNFAIP3 | NM_006290 | tumor necrosis factor, alpha-induced protein 3 |
| SPIN1 | NM_006717 | spindlin 1 |
| TOMM22 | NM_020243 | translocase of outer mitochondrial membrane 22 homolog (yeast) |
| TPCN2 | NM_139075 | two pore segment channel 2 |
| RAD51 | NM_001164269 | RAD51 homolog (S. cerevisiae) |
| LHFPL2 | NM_005779 | lipoma HMGIC fusion partner-like 2 |
| ARGFX | NM_001012659 | arginine-fifty homeobox |
| C18orf63 | NM_001174123 | chromosome 18 open reading frame 63 |
| ANK3 | NM_001149 | ankyrin 3, node of Ranvier (ankyrin G) |
| DUSP4 | NM_001394 | dual specificity phosphatase 4 |
| ARF6 | NM_001663 | ADP-ribosylation factor 6 |
| CREB1 | NM_004379 | cAMP responsive element binding protein 1 |
| PHF2 | NM_005392 | PHD finger protein 2 |
| SEPT9 | NM_001113491 | septin 9 |
| LEPREL1 | NM_001134418 | leprecan-like 1 |
| ELMOD1 | NM_001130037 | ELMO/CED-12 domain containing 1 |
| RPL27A | NM_000990 | ribosomal protein L27a |
| RIF1 | NM_001177663 | RAP1 interacting factor homolog (yeast) |
| PLA2G4E | NM_001206670 | phospholipase A2, group IVE |
| RBM27 | NM_018989 | RNA binding motif protein 27 |
| PTPRB | NM_001109754 | protein tyrosine phosphatase, receptor type, B |
| DNAJC3 | NM_006260 | DnaJ (Hsp40) homolog, subfamily C, member 3 |
| RBBP4 | NM_001135255 | retinoblastoma binding protein 4 |
| PLXNC1 | NM_005761 | plexin C1 |
| KREMEN1 | NM_032045 | kringle containing transmembrane protein 1 |
| ABCB5 | NM_001163942 | ATP-binding cassette, sub-family B (MDR/TAP), member 5 |
| CSNK1D | NM_001893 | casein kinase 1, delta |
| ZSCAN22 | NM_181846 | zinc finger and SCAN domain containing 22 |
| MYRIP | NM_015460 | myosin VIIA and Rab interacting protein |
| MAEL | NM_032858 | maelstrom homolog (Drosophila) |
| INHBB | NM_002193 | inhibin, beta B |
| SRSF1 | NM_001078166 | serine/arginine-rich splicing factor 1 |
| VAMP3 | NM_004781 | vesicle-associated membrane protein 3 (cellubrevin) |
| ZC3H4 | NM_015168 | zinc finger CCCH-type containing 4 |
| ERO1L | NM_014584 | ERO1-like (S. cerevisiae) |
| CRLS1 | NM_001127458 | cardiolipin synthase 1 |
| SENP7 | NM_001077203 | SUMO1/sentrin specific peptidase 7 |
| FAM170A | NM_001163991 | family with sequence similarity 170, member A |
| MMP2 | NM_001127891 | matrix metallopeptidase 2 (gelatinase A, 72kDa gelatinase, 72kDa type IV |

| | | |
|---|---|---|
| TSC22D2 | NM_014779 | TSC22 domain family, member 2 |
| DDX43 | NM_018665 | DEAD (Asp-Glu-Ala-Asp) box polypeptide 43 |
| PTCD2 | NM_024754 | pentatricopeptide repeat domain 2 |
| CRISPLD1 | NM_031461 | cysteine-rich secretory protein LCCL domain containing 1 |
| MYOZ3 | NM_001122853 | myozenin 3 |
| GATC | NM_176818 | glutamyl-tRNA(Gln) amidotransferase, subunit C homolog (bacterial) |
| ADAMTS2 | NM_014244 | ADAM metallopeptidase with thrombospondin type 1 motif, 2 |
| SLC5A12 | NM_178498 | solute carrier family 5 (sodium/glucose cotransporter), member 12 |
| LIFR | NM_001127671 | leukemia inhibitory factor receptor alpha |
| ST8SIA3 | NM_015879 | ST8 alpha-N-acetyl-neuraminide alpha-2,8-sialyltransferase 3 |
| ZMAT3 | NM_022470 | zinc finger, matrin-type 3 |
| SSR1 | NM_003144 | signal sequence receptor, alpha |
| UMPS | NM_000373 | uridine monophosphate synthetase |
| TCF4 | NM_001083962 | transcription factor 4 |
| UBN2 | NM_173569 | ubinuclein 2 |
| G3BP2 | NM_012297 | GTPase activating protein (SH3 domain) binding protein 2 |
| PHF6 | NM_001015877 | PHD finger protein 6 |
| TSGA14 | NM_018718 | testis specific, 14 |
| LIN28B | NM_001004317 | lin-28 homolog B (C. elegans) |
| ENSA | NM_004436 | endosulfine alpha |
| TADA2B | NM_152293 | transcriptional adaptor 2B |
| TSPYL4 | NM_021648 | TSPY-like 4 |
| FZD3 | NM_017412 | frizzled family receptor 3 |
| SLA | NM_001045556 | Src-like-adaptor |
| LMOD1 | NM_012134 | leiomodin 1 (smooth muscle) |
| JAKMIP3 | NM_001105521 | Janus kinase and microtubule interacting protein 3 |
| SLC25A36 | NM_001104647 | solute carrier family 25, member 36 |
| C1orf226 | NM_001085375 | chromosome 1 open reading frame 226 |
| KLHL8 | NM_020803 | kelch-like 8 (Drosophila) |
| ZBTB41 | NM_194314 | zinc finger and BTB domain containing 41 |
| MED13 | NM_005121 | mediator complex subunit 13 |
| PEX5L | NM_016559 | peroxisomal biogenesis factor 5-like |
| SETD7 | NM_030648 | SET domain containing (lysine methyltransferase) 7 |
| SHOC2 | NM_007373 | soc-2 suppressor of clear homolog (C. elegans) |
| CNO | NM_018366 | cappuccino homolog (mouse) |
| DPY19L1 | NM_015283 | dpy-19-like 1 (C. elegans) |
| NCAM2 | NM_004540 | neural cell adhesion molecule 2 |
| SPCS2 | NM_014752 | signal peptidase complex subunit 2 homolog (S. cerevisiae) |
| ZCCHC4 | NM_024936 | zinc finger, CCHC domain containing 4 |

| | | |
|---|---|---|
| MRI1 | NM_001031727 | methylthioribose-1-phosphate isomerase homolog (S. cerevisiae) |
| CHD2 | NM_001271 | chromodomain helicase DNA binding protein 2 |
| PTCH1 | NM_000264 | patched 1 |
| LRCH2 | NM_020871 | leucine-rich repeats and calponin homology (CH) domain containing 2 |
| ZFAND5 | NM_001102420 | zinc finger, AN1-type domain 5 |
| SDC1 | NM_001006946 | syndecan 1 |
| SLC16A2 | NM_006517 | solute carrier family 16, member 2 (monocarboxylic acid transporter 8) |
| SLC25A21 | NM_030631 | solute carrier family 25 (mitochondrial oxodicarboxylate carrier), member 21 |
| B7H6 | NM_001202439 | B7 homolog 6 |
| VDAC1 | NM_003374 | voltage-dependent anion channel 1 |
| GABARAPL1 | NM_031412 | GABA(A) receptor-associated protein like 1 |
| SLC25A26 | NM_001164796 | solute carrier family 25, member 26 |
| ZNF558 | NM_144693 | zinc finger protein 558 |
| C9orf102 | NM_001010895 | chromosome 9 open reading frame 102 |
| PPPDE1 | NM_016076 | PPPDE peptidase domain containing 1 |
| DNM3 | NM_001136127 | dynamin 3 |
| MOBKL1A | NM_173468 | MOB1, Mps One Binder kinase activator-like 1A (yeast) |
| ZNF134 | NM_003435 | zinc finger protein 134 |
| METAP2 | NM_006838 | methionyl aminopeptidase 2 |
| ZAK | NM_016653 | sterile alpha motif and leucine zipper containing kinase AZK |
| PFN2 | NM_053024 | profilin 2 |
| SC5DL | NM_001024956 | sterol-C5-desaturase (ERG3 delta-5-desaturase homolog, S. cerevisiae)-like |
| NRG3 | NM_001010848 | neuregulin 3 |
| RHOBTB2 | NM_001160036 | Rho-related BTB domain containing 2 |
| FAM71C | NM_153364 | family with sequence similarity 71, member C |
| GATAD2B | NM_020699 | GATA zinc finger domain containing 2B |
| KIF14 | NM_014875 | kinesin family member 14 |
| PIGR | NM_002644 | polymeric immunoglobulin receptor |
| SASH1 | NM_015278 | SAM and SH3 domain containing 1 |
| MCL1 | NM_001197320 | myeloid cell leukemia sequence 1 (BCL2-related) |
| ELOVL4 | NM_022726 | ELOVL fatty acid elongase 4 |
| ZNF483 | NM_001007169 | zinc finger protein 483 |
| CFTR | NM_000492 | cystic fibrosis transmembrane conductance regulator (ATP-binding cassette sub- |
| TFDP2 | NM_001178138 | transcription factor Dp-2 (E2F dimerization partner 2) |
| TMEM182 | NM_144632 | transmembrane protein 182 |
| GPR114 | NM_153837 | G protein-coupled receptor 114 |
| APBA1 | NM_001163 | amyloid beta (A4) precursor protein-binding, family A, member 1 |
| RPS6KB2 | NM_003952 | ribosomal protein S6 kinase, 70kDa, polypeptide 2 |
| PSTPIP2 | NM_024430 | proline-serine-threonine phosphatase interacting protein 2 |

| | | |
|---|---|---|
| NAA38 | NM_016200 | N(alpha)-acetyltransferase 38, NatC auxiliary subunit |
| PDRG1 | NM_030815 | p53 and DNA-damage regulated 1 |
| METTL8 | NM_024770 | methyltransferase like 8 |
| FLYWCH1 | NM_032296 | FLYWCH-type zinc finger 1 |
| TMED2 | NM_006815 | transmembrane emp24 domain trafficking protein 2 |
| KIAA1715 | NM_030650 | KIAA1715 |
| STAT6 | NM_001178078 | signal transducer and activator of transcription 6, interleukin-4 induced |
| ZNF248 | NM_021045 | zinc finger protein 248 |
| VANGL1 | NM_001172411 | vang-like 1 (van gogh, Drosophila) |
| GRHL1 | NM_198182 | grainyhead-like 1 (Drosophila) |
| ZNF323 | NM_001135215 | zinc finger protein 323 |
| RASAL3 | NM_022904 | RAS protein activator like 3 |
| RNF219 | NM_024546 | ring finger protein 219 |
| SEPT14 | NM_207366 | septin 14 |
| RSL1D1 | NM_015659 | ribosomal L1 domain containing 1 |
| NLN | NM_020726 | neurolysin (metallopeptidase M3 family) |
| KIAA0513 | NM_014732 | KIAA0513 |
| CHIC1 | NM_001039840 | cysteine-rich hydrophobic domain 1 |
| MCTS1 | NM_001137554 | malignant T cell amplified sequence 1 |
| GPAM | NM_020918 | glycerol-3-phosphate acyltransferase, mitochondrial |
| ITGB8 | NM_002214 | integrin, beta 8 |
| SOX6 | NM_001145811 | SRY (sex determining region Y)-box 6 |
| RAPH1 | NM_213589 | Ras association (RalGDS/AF-6) and pleckstrin homology domains 1 |
| UBASH3B | NM_032873 | ubiquitin associated and SH3 domain containing B |
| FAM84A | NM_145175 | family with sequence similarity 84, member A |
| MAP3K2 | NM_006609 | mitogen-activated protein kinase kinase kinase 2 |
| IRS1 | NM_005544 | insulin receptor substrate 1 |
| KLK2 | NM_001002231 | kallikrein-related peptidase 2 |
| PSEN1 | NM_000021 | presenilin 1 |
| KCNE3 | NM_005472 | potassium voltage-gated channel, Isk-related family, member 3 |
| TC2N | NM_001128595 | tandem C2 domains, nuclear |
| TTC14 | NM_001042601 | tetratricopeptide repeat domain 14 |
| SOSTDC1 | NM_015464 | sclerostin domain containing 1 |
| SLC39A10 | NM_001127257 | solute carrier family 39 (zinc transporter), member 10 |
| XYLT2 | NM_022167 | xylosyltransferase II |
| PIK3R1 | NM_001242466 | phosphoinositide-3-kinase, regulatory subunit 1 (alpha) |
| HCP5 | NM_006674 | HLA complex P5 |
| TET2 | NM_001127208 | tet oncogene family member 2 |
| KIAA1751 | NM_001080484 | KIAA1751 |

| | | |
|---|---|---|
| MST4 | NM_001042452 | serine/threonine protein kinase MST4 |
| ANTXR2 | NM_058172 | anthrax toxin receptor 2 |
| ZNF780A | NM_001010880 | zinc finger protein 780A |
| LIN9 | NM_173083 | lin-9 homolog (C. elegans) |
| CLEC12B | NM_205852 | C-type lectin domain family 12, member B |
| CCNI2 | NM_001039780 | cyclin I family, member 2 |
| C3orf71 | NM_001123040 | chromosome 3 open reading frame 71 |
| RGL1 | NM_015149 | ral guanine nucleotide dissociation stimulator-like 1 |
| GATA3 | NM_001002295 | GATA binding protein 3 |
| SRPK1 | NM_003137 | SRSF protein kinase 1 |
| DYNLT3 | NM_006520 | dynein, light chain, Tctex-type 3 |
| ARIH1 | NM_005744 | ariadne homolog, ubiquitin-conjugating enzyme E2 binding protein, 1 (Drosophila) |
| MRPL42 | NM_014050 | mitochondrial ribosomal protein L42 |
| HNRNPC | NM_001077442 | heterogeneous nuclear ribonucleoprotein C (C1/C2) |
| POLH | NM_006502 | polymerase (DNA directed), eta |
| PHF7 | NM_016483 | PHD finger protein 7 |
| FERMT1 | NM_017671 | fermitin family member 1 |
| C21orf62 | NM_001162495 | chromosome 21 open reading frame 62 |
| SAMD8 | NM_001174156 | sterile alpha motif domain containing 8 |
| GOLGA6C | NM_001164404 | golgin A6 family, member C |
| GPM6A | NM_005277 | glycoprotein M6A |
| PACSIN1 | NM_001199583 | protein kinase C and casein kinase substrate in neurons 1 |
| MCAM | NM_006500 | melanoma cell adhesion molecule |
| SRSF10 | NM_001191006 | serine/arginine-rich splicing factor 10 |
| SLC17A6 | NM_020346 | solute carrier family 17 (sodium-dependent inorganic phosphate cotransporter), |
| DSC2 | NM_004949 | desmocollin 2 |
| KIAA0494 | NM_014774 | KIAA0494 |
| ZBTB24 | NM_014797 | zinc finger and BTB domain containing 24 |
| SUSD5 | NM_015551 | sushi domain containing 5 |
| C10orf26 | NM_001083913 | chromosome 10 open reading frame 26 |
| OTUB2 | NM_023112 | OTU domain, ubiquitin aldehyde binding 2 |
| DGCR8 | NM_001190326 | DiGeorge syndrome critical region gene 8 |
| KPNA1 | NM_002264 | karyopherin alpha 1 (importin alpha 5) |
| FBXO28 | NM_001136115 | F-box protein 28 |
| MAVS | NM_001206491 | mitochondrial antiviral signaling protein |
| PGM3 | NM_001199917 | phosphoglucomutase 3 |
| CD164 | NM_001142401 | CD164 molecule, sialomucin |
| SAV1 | NM_021818 | salvador homolog 1 (Drosophila) |
| DTNA | NM_001198941 | dystrobrevin, alpha |

| | | |
|---|---|---|
| TBC1D14 | NM_001113361 | TBC1 domain family, member 14 |
| ODZ3 | NM_001080477 | odz, odd Oz/ten-m homolog 3 (Drosophila) |
| ECT2L | NM_001077706 | epithelial cell transforming sequence 2 oncogene-like |
| C17orf96 | NM_001130677 | chromosome 17 open reading frame 96 |
| PIK3R3 | NM_001114172 | phosphoinositide-3-kinase, regulatory subunit 3 (gamma) |
| NLGN4Y | NM_001206850 | neuroligin 4, Y-linked |
| RALGPS2 | NM_152663 | Ral GEF with PH domain and SH3 binding motif 2 |
| TFRC | NM_001128148 | transferrin receptor (p90, CD71) |
| JMJD6 | NM_001081461 | jumonji domain containing 6 |
| ZFP1 | NM_153688 | zinc finger protein 1 homolog (mouse) |
| PI15 | NM_015886 | peptidase inhibitor 15 |
| TMPRSS4 | NM_001083947 | transmembrane protease, serine 4 |
| ADRBK2 | NM_005160 | adrenergic, beta, receptor kinase 2 |
| EPHA7 | NM_004440 | EPH receptor A7 |
| HDHD1 | NM_001135565 | haloacid dehalogenase-like hydrolase domain containing 1 |
| PHF8 | NM_001184896 | PHD finger protein 8 |
| UBE2W | NM_001001481 | ubiquitin-conjugating enzyme E2W (putative) |
| FNIP1 | NM_001008738 | folliculin interacting protein 1 |
| FMNL2 | NM_052905 | formin-like 2 |
| C1orf216 | NM_152374 | chromosome 1 open reading frame 216 |
| AMMECR1 | NM_001025580 | Alport syndrome, mental retardation, midface hypoplasia and elliptocytosis chromosomal region gene 1 |
| PTPRJ | NM_002843 | protein tyrosine phosphatase, receptor type, J |
| GAS7 | NM_001130831 | growth arrest-specific 7 |
| PLCB1 | NM_015192 | phospholipase C, beta 1 (phosphoinositide-specific) |
| ZNF117 | NM_015852 | zinc finger protein 117 |
| ANKDD1A | NM_182703 | ankyrin repeat and death domain containing 1A |
| C3orf62 | NM_198562 | chromosome 3 open reading frame 62 |
| B3GALNT2 | NM_152490 | beta-1,3-N-acetylgalactosaminyltransferase 2 |
| ZNF84 | NM_001127372 | zinc finger protein 84 |
| FRAS1 | NM_025074 | Fraser syndrome 1 |
| RPS6KA3 | NM_004586 | ribosomal protein S6 kinase, 90kDa, polypeptide 3 |
| DCAF5 | NM_003861 | DDB1 and CUL4 associated factor 5 |
| PRLR | NM_000949 | prolactin receptor |
| SMAD2 | NM_001003652 | SMAD family member 2 |
| REST | NM_001193508 | RE1-silencing transcription factor |
| SLC7A6 | NM_001076785 | solute carrier family 7 (amino acid transporter light chain, y+L system), member 6 |
| RNF144A | NM_014746 | ring finger protein 144A |
| ZNF652 | NM_001145365 | zinc finger protein 652 |

| | | |
|---|---|---|
| FBXW2 | NM_012164 | F-box and WD repeat domain containing 2 |
| SBNO1 | NM_001167856 | strawberry notch homolog 1 (Drosophila) |
| KATNAL1 | NM_001014380 | katanin p60 subunit A-like 1 |
| NAV1 | NM_001167738 | neuron navigator 1 |
| GXYLT1 | NM_001099650 | glucoside xylosyltransferase 1 |
| ZNF621 | NM_001098414 | zinc finger protein 621 |
| C4orf40 | NM_214711 | chromosome 4 open reading frame 40 |
| SAMD4A | NM_001161576 | sterile alpha motif domain containing 4A |
| MASP1 | NM_001879 | mannan-binding lectin serine peptidase 1 (C4/C2 activating component of Ra-reactive |
| VSTM4 | NM_001031746 | V-set and transmembrane domain containing 4 |
| TBC1D15 | NM_001146213 | TBC1 domain family, member 15 |
| ZNF770 | NM_014106 | zinc finger protein 770 |
| SLC1A3 | NM_001166695 | solute carrier family 1 (glial high affinity glutamate transporter), member 3 |
| ZHX2 | NM_014943 | zinc fingers and homeoboxes 2 |
| RPGRIP1L | NM_001127897 | RPGRIP1-like |
| EPN3 | NM_017957 | epsin 3 |
| DYRK2 | NM_003583 | dual-specificity tyrosine-(Y)-phosphorylation regulated kinase 2 |
| RANGAP1 | NM_002883 | Ran GTPase activating protein 1 |
| SV2A | NM_014849 | synaptic vesicle glycoprotein 2A |
| HECA | NM_016217 | headcase homolog (Drosophila) |
| VTA1 | NM_016485 | Vps20-associated 1 homolog (S. cerevisiae) |
| HIPK1 | NM_181358 | homeodomain interacting protein kinase 1 |
| LYRM2 | NM_020466 | LYR motif containing 2 |
| C7orf16 | NM_001145123 | chromosome 7 open reading frame 16 |
| HEPHL1 | NM_001098672 | hephaestin-like 1 |
| EIF1AX | NM_001412 | eukaryotic translation initiation factor 1A, X-linked |
| PRKAR1A | NM_002734 | protein kinase, cAMP-dependent, regulatory, type I, alpha (tissue specific extinguisher |
| PHLDB2 | NM_001134437 | pleckstrin homology-like domain, family B, member 2 |
| NRG4 | NM_138573 | neuregulin 4 |
| RWDD4 | NM_152682 | RWD domain containing 4 |
| ACACA | NM_198834 | acetyl-CoA carboxylase alpha |
| TMBIM1 | NM_022152 | transmembrane BAX inhibitor motif containing 1 |
| GGCT | NM_001199815 | gamma-glutamylcyclotransferase |
| TP53INP1 | NM_001135733 | tumor protein p53 inducible nuclear protein 1 |
| CLN8 | NM_018941 | ceroid-lipofuscinosis, neuronal 8 (epilepsy, progressive with mental retardation) |
| MAP3K3 | NM_002401 | mitogen-activated protein kinase kinase kinase 3 |
| PI4KB | NM_001198773 | phosphatidylinositol 4-kinase, catalytic, beta |
| KIAA1310 | NM_001115016 | KIAA1310 |
| IL36G | NM_019618 | interleukin 36, gamma |

| | | |
|---|---|---|
| SOX13 | NM_005686 | SRY (sex determining region Y)-box 13 |
| PLD6 | NM_178836 | phospholipase D family, member 6 |
| SMYD4 | NM_052928 | SET and MYND domain containing 4 |
| LUZP2 | NM_001009909 | leucine zipper protein 2 |
| PHF14 | NM_014660 | PHD finger protein 14 |
| HDAC4 | NM_006037 | histone deacetylase 4 |
| GOPC | NM_001017408 | golgi-associated PDZ and coiled-coil motif containing |
| IGF1 | NM_000618 | insulin-like growth factor 1 (somatomedin C) |
| NLGN4X | NM_020742 | neuroligin 4, X-linked |
| NCEH1 | NM_001146276 | neutral cholesterol ester hydrolase 1 |
| KIT | NM_000222 | v-kit Hardy-Zuckerman 4 feline sarcoma viral oncogene homolog |
| FAM198B | NM_001031700 | family with sequence similarity 198, member B |
| CLIC4 | NM_013943 | chloride intracellular channel 4 |
| GNG2 | NM_053064 | guanine nucleotide binding protein (G protein), gamma 2 |
| BMPER | NM_133468 | BMP binding endothelial regulator |
| SMC4 | NM_001002800 | structural maintenance of chromosomes 4 |
| SEPT6 | NM_015129 | septin 6 |
| KCTD9 | NM_017634 | potassium channel tetramerisation domain containing 9 |
| METTL16 | NM_024086 | methyltransferase like 16 |
| ZEB1 | NM_001128128 | zinc finger E-box binding homeobox 1 |
| TRIM32 | NM_001099679 | tripartite motif containing 32 |
| TMEM139 | NM_001242773 | transmembrane protein 139 |
| SLFN13 | NM_144682 | schlafen family member 13 |
| ETF1 | NM_004730 | eukaryotic translation termination factor 1 |
| TP63 | NM_001114978 | tumor protein p63 |
| CYBB | NM_000397 | cytochrome b-245, beta polypeptide |
| STX7 | NM_003569 | syntaxin 7 |
| KLHL29 | NM_052920 | kelch-like 29 (Drosophila) |
| C3orf23 | NM_173826 | chromosome 3 open reading frame 23 |
| DACH1 | NM_004392 | dachshund homolog 1 (Drosophila) |
| ATP8A1 | NM_001105529 | ATPase, aminophospholipid transporter (APLT), class I, type 8A, member 1 |
| TMOD3 | NM_014547 | tropomodulin 3 (ubiquitous) |
| BVES | NM_001199563 | blood vessel epicardial substance |
| PAPOLG | NM_022894 | poly(A) polymerase gamma |
| CLIP3 | NM_001199570 | CAP-GLY domain containing linker protein 3 |
| PDGFD | NM_025208 | platelet derived growth factor D |
| BICD2 | NM_001003800 | bicaudal D homolog 2 (Drosophila) |
| ELMO1 | NM_001039459 | engulfment and cell motility 1 |
| XPR1 | NM_001135669 | xenotropic and polytropic retrovirus receptor 1 |

| | | |
|---|---|---|
| SEL1L | NM_005065 | sel-1 suppressor of lin-12-like (C. elegans) |
| C17orf63 | NM_001077498 | chromosome 17 open reading frame 63 |
| EPB41L5 | NM_001184937 | erythrocyte membrane protein band 4.1 like 5 |
| UNC80 | NM_032504 | unc-80 homolog (C. elegans) |
| BMPR2 | NM_001204 | bone morphogenetic protein receptor, type II (serine/threonine kinase) |
| GPCPD1 | NM_019593 | glycerophosphocholine phosphodiesterase GDE1 homolog (S. cerevisiae) |
| GPR85 | NM_001146265 | G protein-coupled receptor 85 |
| PAG1 | NM_018440 | phosphoprotein associated with glycosphingolipid microdomains 1 |
| MDM2 | NM_002392 | Mdm2 p53 binding protein homolog (mouse) |
| ACVR1C | NM_001111031 | activin A receptor, type IC |
| JMY | NM_152405 | junction mediating and regulatory protein, p53 cofactor |
| FGFR1 | NM_001174063 | fibroblast growth factor receptor 1 |
| C3orf70 | NM_001025266 | chromosome 3 open reading frame 70 |
| AKAP11 | NM_016248 | A kinase (PRKA) anchor protein 11 |
| APBB2 | NM_001166050 | amyloid beta (A4) precursor protein-binding, family B, member 2 |
| ARSD | NM_001669 | arylsulfatase D |
| ATP2B4 | NM_001001396 | ATPase, Ca++ transporting, plasma membrane 4 |
| BACH1 | NM_001186 | BTB and CNC homology 1, basic leucine zipper transcription factor 1 |
| C18orf1 | NM_001003674 | chromosome 18 open reading frame 1 |
| CACNB4 | NM_000726 | calcium channel, voltage-dependent, beta 4 subunit |
| SLC31A1 | NM_001859 | solute carrier family 31 (copper transporters), member 1 |
| ETV6 | NM_001987 | ets variant 6 |
| GALNT2 | NM_004481 | UDP-N-acetyl-alpha-D-galactosamine:polypeptide N-acetylgalactosaminyltransferase |
| SMAD3 | NM_001145102 | SMAD family member 3 |
| MKLN1 | NM_001145354 | muskelin 1, intracellular mediator containing kelch motifs |
| NFIA | NM_001134673 | nuclear factor I/A |
| NFIX | NM_002501 | nuclear factor I/X (CCAAT-binding transcription factor) |
| PRKAB2 | NM_005399 | protein kinase, AMP-activated, beta 2 non-catalytic subunit |
| RREB1 | NM_001003698 | ras responsive element binding protein 1 |
| TEAD1 | NM_021961 | TEA domain family member 1 (SV40 transcriptional enhancer factor) |
| TLL1 | NM_012464 | tolloid-like 1 |
| ZFX | NM_001178084 | zinc finger protein, X-linked |
| UNC5C | NM_003728 | unc-5 homolog C (C. elegans) |
| SPTLC2 | NM_004863 | serine palmitoyltransferase, long chain base subunit 2 |
| ULK2 | NM_014683 | unc-51-like kinase 2 (C. elegans) |
| EPM2AIP1 | NM_014805 | EPM2A (laforin) interacting protein 1 |
| AKT3 | NM_005465 | v-akt murine thymoma viral oncogene homolog 3 (protein kinase B, gamma) |
| GPC6 | NM_005708 | glypican 6 |
| DZIP1 | NM_014934 | DAZ interacting protein 1 |

| | | |
|---|---|---|
| ZNF609 | NM_015042 | zinc finger protein 609 |
| TNRC6B | NM_001024843 | trinucleotide repeat containing 6B |
| NFASC | NM_001005388 | neurofascin |
| TBC1D30 | NM_015279 | TBC1 domain family, member 30 |
| SIRT5 | NM_001193267 | sirtuin 5 |
| ICMT | NM_012405 | isoprenylcysteine carboxyl methyltransferase |
| DSTYK | NM_015375 | dual serine/threonine and tyrosine protein kinase |
| CNTNAP2 | NM_014141 | contactin associated protein-like 2 |
| AFF4 | NM_014423 | AF4/FMR2 family, member 4 |
| RAB8B | NM_016530 | RAB8B, member RAS oncogene family |
| SGTB | NM_019072 | small glutamine-rich tetratricopeptide repeat (TPR)-containing, beta |
| FAM46C | NM_017709 | family with sequence similarity 46, member C |
| STX17 | NM_017919 | syntaxin 17 |
| DHTKD1 | NM_018706 | dehydrogenase E1 and transketolase domain containing 1 |
| BBX | NM_001142568 | bobby sox homolog (Drosophila) |
| VANGL2 | NM_020335 | vang-like 2 (van gogh, Drosophila) |
| KIAA1456 | NM_001099677 | KIAA1456 |
| BACH2 | NM_001170794 | BTB and CNC homology 1, basic leucine zipper transcription factor 2 |
| LMBR1 | NM_022458 | limb region 1 homolog (mouse) |
| TRAK2 | NM_015049 | trafficking protein, kinesin binding 2 |
| NOL9 | NM_024654 | nucleolar protein 9 |
| QSER1 | NM_001076786 | glutamine and serine rich 1 |
| CSRNP3 | NM_001172173 | cysteine-serine-rich nuclear protein 3 |
| DNAL1 | NM_001201366 | dynein, axonemal, light chain 1 |
| ZDHHC18 | NM_032283 | zinc finger, DHHC-type containing 18 |
| TBRG1 | NM_032811 | transforming growth factor beta regulator 1 |
| PAQR8 | NM_133367 | progestin and adipoQ receptor family member VIII |
| MYSM1 | NM_001085487 | Myb-like, SWIRM and MPN domains 1 |
| TSHZ2 | NM_001193421 | teashirt zinc finger homeobox 2 |
| SLITRK4 | NM_001184749 | SLIT and NTRK-like family, member 4 |
| MIPOL1 | NM_001195296 | mirror-image polydactyly 1 |
| SLC30A7 | NM_001144884 | solute carrier family 30 (zinc transporter), member 7 |
| ALDH1L2 | NM_001034173 | aldehyde dehydrogenase 1 family, member L2 |
| MACC1 | NM_182762 | metastasis associated in colon cancer 1 |
| ANKRD34C | NM_001146341 | ankyrin repeat domain 34C |
| KLHL31 | NM_001003760 | kelch-like 31 (Drosophila) |
| ZBTB34 | NM_001099270 | zinc finger and BTB domain containing 34 |
| RNF165 | NM_152470 | ring finger protein 165 |
| POTEM | NM_001145442 | POTE ankyrin domain family, member M |

| | | |
|---|---|---|
| THSD7A | NM_015204 | thrombospondin, type I, domain containing 7A |
| TNFRSF9 | NM_001561 | tumor necrosis factor receptor superfamily, member 9 |
| MON2 | NM_015026 | MON2 homolog (S. cerevisiae) |
| RBMS3 | NM_001003792 | RNA binding motif, single stranded interacting protein 3 |
| ZXDC | NM_001040653 | ZXD family zinc finger C |
| ZBTB44 | NM_014155 | zinc finger and BTB domain containing 44 |
| ZNF805 | NM_001023563 | zinc finger protein 805 |
| CTSC | NM_001114173 | cathepsin C |
| KDM5A | NM_001042603 | lysine (K)-specific demethylase 5A |
| C9orf5 | NM_032012 | chromosome 9 open reading frame 5 |
| PPP1R9A | NM_001166160 | protein phosphatase 1, regulatory (inhibitor) subunit 9A |
| FBXL20 | NM_001184906 | F-box and leucine-rich repeat protein 20 |
| C6orf89 | NM_152734 | chromosome 6 open reading frame 89 |
| CNOT6L | NM_144571 | CCR4-NOT transcription complex, subunit 6-like |
| KSR2 | NM_173598 | kinase suppressor of ras 2 |
| FMN1 | NM_001103184 | formin 1 |
| PAFAH1B2 | NM_002572 | platelet-activating factor acetylhydrolase 1b, catalytic subunit 2 (30kDa) |
| OLA1 | NM_001011708 | Obg-like ATPase 1 |
| C22orf41 | NM_001123225 | chromosome 22 open reading frame 41 |
| SYN3 | NM_001135774 | synapsin III |
| ANGEL1 | NM_015305 | angel homolog 1 (Drosophila) |
| AP1S3 | NM_001039569 | adaptor-related protein complex 1, sigma 3 subunit |
| C18orf25 | NM_001008239 | chromosome 18 open reading frame 25 |
| SUB1 | NM_006713 | SUB1 homolog (S. cerevisiae) |
| TBC1D2B | NM_015079 | TBC1 domain family, member 2B |
| SAR1A | NM_001142648 | SAR1 homolog A (S. cerevisiae) |
| KIAA1804 | NM_032435 | mixed lineage kinase 4 |
| SORBS1 | NM_001034954 | sorbin and SH3 domain containing 1 |
| ARHGEF40 | NM_018071 | Rho guanine nucleotide exchange factor (GEF) 40 |
| PANX2 | NM_001160300 | pannexin 2 |
| C6orf97 | NM_025059 | chromosome 6 open reading frame 97 |
| CDKN1B | NM_004064 | cyclin-dependent kinase inhibitor 1B (p27, Kip1) |
| RIPK4 | NM_020639 | receptor-interacting serine-threonine kinase 4 |
| ATAD2B | NM_001242338 | ATPase family, AAA domain containing 2B |
| C9orf57 | NM_001128618 | chromosome 9 open reading frame 57 |
| CTCFL | NM_080618 | CCCTC-binding factor (zinc finger protein)-like |
| TLK2 | NM_001112707 | tousled-like kinase 2 |
| KLHL28 | NM_017658 | kelch-like 28 (Drosophila) |
| METTL4 | NM_022840 | methyltransferase like 4 |

| | | |
|---|---|---|
| LRRC8C | NM_032270 | leucine rich repeat containing 8 family, member C |
| ZNF208 | NM_007153 | zinc finger protein 208 |
| ERP44 | NM_015051 | endoplasmic reticulum protein 44 |
| COG6 | NM_001145079 | component of oligomeric golgi complex 6 |
| DHX37 | NM_032656 | DEAH (Asp-Glu-Ala-His) box polypeptide 37 |
| EFNB2 | NM_004093 | ephrin-B2 |
| ANGPTL2 | NM_012098 | angiopoietin-like 2 |
| AGGF1 | NM_018046 | angiogenic factor with G patch and FHA domains 1 |
| UHMK1 | NM_001184763 | U2AF homology motif (UHM) kinase 1 |
| TMEM170B | NM_001100829 | transmembrane protein 170B |
| EYA4 | NM_004100 | eyes absent homolog 4 (Drosophila) |
| MPP5 | NM_022474 | membrane protein, palmitoylated 5 (MAGUK p55 subfamily member 5) |
| COX6B2 | NM_144613 | cytochrome c oxidase subunit VIb polypeptide 2 (testis) |
| WTH3DI | NM_001077637 | RAB6C-like |
| RBFOX2 | NM_001031695 | RNA binding protein, fox-1 homolog (C. elegans) 2 |
| LDB3 | NM_001080114 | LIM domain binding 3 |
| RASSF8 | NM_001164746 | Ras association (RalGDS/AF-6) domain family (N-terminal) member 8 |
| C7orf29 | NM_138434 | chromosome 7 open reading frame 29 |
| MBD2 | NM_003927 | methyl-CpG binding domain protein 2 |
| NPTXR | NM_014293 | neuronal pentraxin receptor |
| CDK12 | NM_015083 | cyclin-dependent kinase 12 |
| HM13 | NM_178582 | histocompatibility (minor) 13 |
| GDNF | NM_000514 | glial cell derived neurotrophic factor |
| KCNJ12 | NM_021012 | potassium inwardly-rectifying channel, subfamily J, member 12 |
| ZNF192 | NM_006298 | zinc finger protein 192 |
| ZEB2 | NM_001171653 | zinc finger E-box binding homeobox 2 |
| CELF1 | NM_001172639 | CUGBP, Elav-like family member 1 |
| C21orf91 | NM_001100420 | chromosome 21 open reading frame 91 |
| CYP1A2 | NM_000761 | cytochrome P450, family 1, subfamily A, polypeptide 2 |
| BHLHE22 | NM_152414 | basic helix-loop-helix family, member e22 |
| SH3GLB1 | NM_001206651 | SH3-domain GRB2-like endophilin B1 |
| AP1AR | NM_001128426 | adaptor-related protein complex 1 associated regulatory protein |
| MECOM | NM_001105077 | MDS1 and EVI1 complex locus |
| WSCD2 | NM_014653 | WSC domain containing 2 |
| KLF12 | NM_007249 | Kruppel-like factor 12 |
| SMAP2 | NM_001198978 | small ArfGAP2 |
| C3orf35 | NM_178339 | chromosome 3 open reading frame 35 |
| OCLN | NM_001205254 | occludin |
| LRRC20 | NM_018205 | leucine rich repeat containing 20 |

| | | |
|---|---|---|
| MAML3 | NM_018717 | mastermind-like 3 (Drosophila) |
| CALD1 | NM_004342 | caldesmon 1 |
| ANO5 | NM_001142649 | anoctamin 5 |
| ACAN | NM_001135 | aggrecan |
| SYS1 | NM_001099791 | SYS1 Golgi-localized integral membrane protein homolog (S. cerevisiae) |
| TMTC2 | NM_152588 | transmembrane and tetratricopeptide repeat containing 2 |
| HEPACAM | NM_152722 | hepatic and glial cell adhesion molecule |
| SS18 | NM_001007559 | synovial sarcoma translocation, chromosome 18 |
| ZDBF2 | NM_020923 | zinc finger, DBF-type containing 2 |
| TBCEL | NM_001130047 | tubulin folding cofactor E-like |
| DAZ1 | NM_004081 | deleted in azoospermia 1 |
| MAP2K4 | NM_003010 | mitogen-activated protein kinase kinase 4 |
| ZBTB6 | NM_006626 | zinc finger and BTB domain containing 6 |
| EDAR | NM_022336 | ectodysplasin A receptor |
| DAZ3 | NM_020364 | deleted in azoospermia 3 |
| DAZ2 | NM_001005785 | deleted in azoospermia 2 |
| DAZ4 | NM_001005375 | deleted in azoospermia 4 |
| CD81 | NM_004356 | CD81 molecule |
| NEGR1 | NM_173808 | neuronal growth regulator 1 |
| PCSK5 | NM_001190482 | proprotein convertase subtilisin/kexin type 5 |
| CHST3 | NM_004273 | carbohydrate (chondroitin 6) sulfotransferase 3 |
| BCL2L15 | NM_001010922 | BCL2-like 15 |
| PSAPL1 | NM_001085382 | prosaposin-like 1 (gene/pseudogene) |
| HIST2H2BE | NM_003528 | histone cluster 2, H2be |
| TMEM135 | NM_001168724 | transmembrane protein 135 |
| KRAS | NM_004985 | v-Ki-ras2 Kirsten rat sarcoma viral oncogene homolog |
| POU2F1 | NM_001198783 | POU class 2 homeobox 1 |
| GPR56 | NM_001145770 | G protein-coupled receptor 56 |
| PCNX | NM_014982 | pecanex homolog (Drosophila) |
| FAR1 | NM_032228 | fatty acyl CoA reductase 1 |
| KIF5C | NM_004522 | kinesin family member 5C |
| SYNJ2BP | NM_018373 | synaptojanin 2 binding protein |
| RIMKLA | NM_173642 | ribosomal modification protein rimK-like family member A |
| NHLRC3 | NM_001012754 | NHL repeat containing 3 |
| AVPR2 | NM_000054 | arginine vasopressin receptor 2 |
| VAMP1 | NM_014231 | vesicle-associated membrane protein 1 (synaptobrevin 1) |
| KIAA1267 | NM_001193465 | KIAA1267 |
| DAZL | NM_001190811 | deleted in azoospermia-like |
| PREPL | NM_001042385 | prolyl endopeptidase-like |

| | | |
|---|---|---|
| LOC100507421 | NM_001195278 | transmembrane protein 178-like |
| MLLT10 | NM_001195626 | myeloid/lymphoid or mixed-lineage leukemia (trithorax homolog, Drosophila); |
| SPIN3 | NM_001010862 | spindlin family, member 3 |
| KCNJ13 | NM_001172416 | potassium inwardly-rectifying channel, subfamily J, member 13 |
| PCLO | NM_033026 | piccolo (presynaptic cytomatrix protein) |
| HUNK | NM_014586 | hormonally up-regulated Neu-associated kinase |
| LAMC3 | NM_006059 | laminin, gamma 3 |
| MAP4K4 | NM_001242559 | mitogen-activated protein kinase kinase kinase kinase 4 |
| TESK2 | NM_007170 | testis-specific kinase 2 |
| SLC7A2 | NM_001008539 | solute carrier family 7 (cationic amino acid transporter, y+ system), member 2 |
| XYLT1 | NM_022166 | xylosyltransferase I |
| IRGQ | NM_001007561 | immunity-related GTPase family, Q |
| NCALD | NM_001040624 | neurocalcin delta |
| CCNG2 | NM_004354 | cyclin G2 |
| CDS2 | NM_003818 | CDP-diacylglycerol synthase (phosphatidate cytidylyltransferase) 2 |
| MYNN | NM_001185118 | myoneurin |
| LCOR | NM_001170765 | ligand dependent nuclear receptor corepressor |
| ARHGAP40 | NM_001164431 | Rho GTPase activating protein 40 |
| PHLDA1 | NM_007350 | pleckstrin homology-like domain, family A, member 1 |
| NRL | NM_006177 | neural retina leucine zipper |
| PAQR5 | NM_001104554 | progestin and adipoQ receptor family member V |
| AP1G1 | NM_001030007 | adaptor-related protein complex 1, gamma 1 subunit |
| AQP1 | NM_001185060 | aquaporin 1 (Colton blood group) |
| CALU | NM_001130674 | calumenin |
| CHM | NM_000390 | choroideremia (Rab escort protein 1) |
| COX15 | NM_004376 | COX15 homolog, cytochrome c oxidase assembly protein (yeast) |
| DOCK3 | NM_004947 | dedicator of cytokinesis 3 |
| PHC2 | NM_004427 | polyhomeotic homolog 2 (Drosophila) |
| EMP2 | NM_001424 | epithelial membrane protein 2 |
| ERBB4 | NM_001042599 | v-erb-a erythroblastic leukemia viral oncogene homolog 4 (avian) |
| ETV1 | NM_001163147 | ets variant 1 |
| GPR12 | NM_005288 | G protein-coupled receptor 12 |
| HTT | NM_002111 | huntingtin |
| AGFG1 | NM_001135187 | ArfGAP with FG repeats 1 |
| KCNN3 | NM_001204087 | potassium intermediate/small conductance calcium-activated channel, subfamily N, |
| LRPAP1 | NM_002337 | low density lipoprotein receptor-related protein associated protein 1 |
| MATN1 | NM_002379 | matrilin 1, cartilage matrix protein |
| CHST6 | NM_021615 | carbohydrate (N-acetylglucosamine 6-O) sulfotransferase 6 |
| NFATC3 | NM_004555 | nuclear factor of activated T-cells, cytoplasmic, calcineurin-dependent 3 |

| | | |
|---|---|---|
| OAS3 | NM_006187 | 2'-5'-oligoadenylate synthetase 3, 100kDa |
| OPCML | NM_001012393 | opioid binding protein/cell adhesion molecule-like |
| PRKAA1 | NM_006251 | protein kinase, AMP-activated, alpha 1 catalytic subunit |
| PRRG1 | NM_000950 | proline rich Gla (G-carboxyglutamic acid) 1 |
| RBM3 | NM_006743 | RNA binding motif (RNP1, RRM) protein 3 |
| RDX | NM_002906 | radixin |
| SLC1A2 | NM_001195728 | solute carrier family 1 (glial high affinity glutamate transporter), member 2 |
| SORL1 | NM_003105 | sortilin-related receptor, L(DLR class) A repeats containing |
| STC1 | NM_003155 | stanniocalcin 1 |
| KLF11 | NM_001177716 | Kruppel-like factor 11 |
| SPAG9 | NM_001130528 | sperm associated antigen 9 |
| MTMR6 | NM_004685 | myotubularin related protein 6 |
| SYNGR2 | NM_004710 | synaptogyrin 2 |
| DLGAP2 | NM_004745 | discs, large (Drosophila) homolog-associated protein 2 |
| HS2ST1 | NM_012262 | heparan sulfate 2-O-sulfotransferase 1 |
| FRMPD4 | NM_014728 | FERM and PDZ domain containing 4 |
| ZBTB5 | NM_014872 | zinc finger and BTB domain containing 5 |
| HCN4 | NM_005477 | hyperpolarization activated cyclic nucleotide-gated potassium channel 4 |
| IKZF1 | NM_001220765 | IKAROS family zinc finger 1 (Ikaros) |
| NEBL | NM_001173484 | nebulette |
| TRAFD1 | NM_001143906 | TRAF-type zinc finger domain containing 1 |
| MSL3 | NM_078628 | male-specific lethal 3 homolog (Drosophila) |
| ABHD2 | NM_007011 | abhydrolase domain containing 2 |
| BAZ2A | NM_013449 | bromodomain adjacent to zinc finger domain, 2A |
| AAK1 | NM_014911 | AP2 associated kinase 1 |
| ZNF365 | NM_014951 | zinc finger protein 365 |
| CD93 | NM_012072 | CD93 molecule |
| SARM1 | NM_015077 | sterile alpha and TIR motif containing 1 |
| TTC28 | NM_001145418 | tetratricopeptide repeat domain 28 |
| SLC24A2 | NM_001193288 | solute carrier family 24 (sodium/potassium/calcium exchanger), member 2 |
| ATXN10 | NM_001167621 | ataxin 10 |
| NSL1 | NM_001042549 | NSL1, MIND kinetochore complex component, homolog (S. cerevisiae) |
| ODZ4 | NM_001098816 | odz, odd Oz/ten-m homolog 4 (Drosophila) |
| SETBP1 | NM_015559 | SET binding protein 1 |
| EIF2C1 | NM_012199 | eukaryotic translation initiation factor 2C, 1 |
| GREM1 | NM_001191322 | gremlin 1 |
| PCDH11X | NM_001168360 | protocadherin 11 X-linked |
| SNX8 | NM_013321 | sorting nexin 8 |
| SEPSECS | NM_016955 | Sep (O-phosphoserine) tRNA:Sec (selenocysteine) tRNA synthase |

| | | |
|---|---|---|
| SAR1B | NM_001033503 | SAR1 homolog B (S. cerevisiae) |
| SHC3 | NM_016848 | SHC (Src homology 2 domain containing) transforming protein 3 |
| TPCN1 | NM_001143819 | two pore segment channel 1 |
| FAM105A | NM_019018 | family with sequence similarity 105, member A |
| IMPAD1 | NM_017813 | inositol monophosphatase domain containing 1 |
| PDPR | NM_017990 | pyruvate dehydrogenase phosphatase regulatory subunit |
| ETNK1 | NM_018638 | ethanolamine kinase 1 |
| TULP4 | NM_001007466 | tubby like protein 4 |
| ZNF286A | NM_001130842 | zinc finger protein 286A |
| TMCC3 | NM_020698 | transmembrane and coiled-coil domain family 3 |
| USP31 | NM_020718 | ubiquitin specific peptidase 31 |
| RPTOR | NM_001163034 | regulatory associated protein of MTOR, complex 1 |
| PCDH19 | NM_001105243 | protocadherin 19 |
| ALPK3 | NM_020778 | alpha-kinase 3 |
| STIM2 | NM_001169117 | stromal interaction molecule 2 |
| ZFP106 | NM_022473 | zinc finger protein 106 homolog (mouse) |
| RMND5A | NM_022780 | required for meiotic nuclear division 5 homolog A (S. cerevisiae) |
| C17orf39 | NM_024052 | chromosome 17 open reading frame 39 |
| ASB13 | NM_024701 | ankyrin repeat and SOCS box containing 13 |
| CLMN | NM_024734 | calmin (calponin-like, transmembrane) |
| MOBKL2B | NM_024761 | MOB1, Mps One Binder kinase activator-like 2B (yeast) |
| L2HGDH | NM_024884 | L-2-hydroxyglutarate dehydrogenase |
| ERMP1 | NM_024896 | endoplasmic reticulum metallopeptidase 1 |
| KLHL15 | NM_030624 | kelch-like 15 (Drosophila) |
| RNF170 | NM_001160223 | ring finger protein 170 |
| PCDH11Y | NM_032973 | protocadherin 11 Y-linked |
| CABLES1 | NM_001100619 | Cdk5 and Abl enzyme substrate 1 |
| MTDH | NM_178812 | metadherin |
| MYOCD | NM_001146312 | myocardin |
| FBXO32 | NM_001242463 | F-box protein 32 |
| NAA30 | NM_001011713 | N(alpha)-acetyltransferase 30, NatC catalytic subunit |
| KLF17 | NM_173484 | Kruppel-like factor 17 |
| C5orf24 | NM_001135586 | chromosome 5 open reading frame 24 |
| CCBE1 | NM_133459 | collagen and calcium binding EGF domains 1 |
| ZNF578 | NM_001099694 | zinc finger protein 578 |
| DIRAS1 | NM_145173 | DIRAS family, GTP-binding RAS-like 1 |
| AMOTL1 | NM_130847 | angiomotin like 1 |
| RASSF6 | NM_177532 | Ras association (RalGDS/AF-6) domain family member 6 |
| QSOX2 | NM_181701 | quiescin Q6 sulfhydryl oxidase 2 |

| | | |
|---|---|---|
| SYNPO2 | NM_001128933 | synaptopodin 2 |
| IBA57 | NM_001010867 | IBA57, iron-sulfur cluster assembly homolog (S. cerevisiae) |
| TET3 | NM_144993 | tet oncogene family member 3 |
| SENP5 | NM_152699 | SUMO1/sentrin specific peptidase 5 |
| GK5 | NM_001039547 | glycerol kinase 5 (putative) |
| SERINC5 | NM_001174072 | serine incorporator 5 |
| PGM2L1 | NM_173582 | phosphoglucomutase 2-like 1 |
| SREK1IP1 | NM_173829 | SREK1-interacting protein 1 |
| PLCXD3 | NM_001005473 | phosphatidylinositol-specific phospholipase C, X domain containing 3 |
| ZNF445 | NM_181489 | zinc finger protein 445 |
| NSUN4 | NM_199044 | NOP2/Sun domain family, member 4 |
| TMPRSS11BNL | NM_001129907 | TMPRSS11B N terminal-like |
| CENPP | NM_001012267 | centromere protein P |
| ZNF286B | NM_001145045 | zinc finger protein 286B |
| SH3PXD2A | NM_014631 | SH3 and PX domains 2A |
| ANKRD13C | NM_030816 | ankyrin repeat domain 13C |
| BCL2 | NM_000633 | B-cell CLL/lymphoma 2 |
| CACNA1E | NM_000721 | calcium channel, voltage-dependent, R type, alpha 1E subunit |
| GABRB2 | NM_000813 | gamma-aminobutyric acid (GABA) A receptor, beta 2 |
| SCAMP1 | NM_004866 | secretory carrier membrane protein 1 |
| IGF2BP1 | NM_001160423 | insulin-like growth factor 2 mRNA binding protein 1 |
| MIB1 | NM_020774 | mindbomb homolog 1 (Drosophila) |
| APOLD1 | NM_001130415 | apolipoprotein L domain containing 1 |
| SORCS1 | NM_001013031 | sortilin-related VPS10 domain containing receptor 1 |
| NUDT16 | NM_001171905 | nudix (nucleoside diphosphate linked moiety X)-type motif 16 |
| GRPEL2 | NM_152407 | GrpE-like 2, mitochondrial (E. coli) |
| CISD2 | NM_001008388 | CDGSH iron sulfur domain 2 |
| ITPKB | NM_002221 | inositol-trisphosphate 3-kinase B |
| NAIP | NM_004536 | NLR family, apoptosis inhibitory protein |
| NECAB1 | NM_022351 | N-terminal EF-hand calcium binding protein 1 |
| MEGF11 | NM_032445 | multiple EGF-like-domains 11 |
| C12orf59 | NM_153022 | chromosome 12 open reading frame 59 |
| RNF2 | NM_007212 | ring finger protein 2 |
| MRPS16 | NM_016065 | mitochondrial ribosomal protein S16 |
| ZNF146 | NM_001099638 | zinc finger protein 146 |
| CD99L2 | NM_001184808 | CD99 molecule-like 2 |
| USP51 | NM_201286 | ubiquitin specific peptidase 51 |
| TNFSF4 | NM_003326 | tumor necrosis factor (ligand) superfamily, member 4 |
| C1GALT1 | NM_020156 | core 1 synthase, glycoprotein-N-acetylgalactosamine 3-beta-galactosyltransferase, 1 |

| | | |
|---|---|---|
| TWISTNB | NM_001002926 | TWIST neighbor |
| ENTPD6 | NM_001114089 | ectonucleoside triphosphate diphosphohydrolase 6 (putative) |
| CHML | NM_001821 | choroideremia-like (Rab escort protein 2) |
| C5orf30 | NM_033211 | chromosome 5 open reading frame 30 |
| NR4A3 | NM_006981 | nuclear receptor subfamily 4, group A, member 3 |
| ADCY7 | NM_001114 | adenylate cyclase 7 |
| LTN1 | NM_015565 | listerin E3 ubiquitin protein ligase 1 |
| RGAG4 | NM_001024455 | retrotransposon gag domain containing 4 |
| IPO7 | NM_006391 | importin 7 |
| ZNF333 | NM_032433 | zinc finger protein 333 |
| H3F3B | NM_005324 | H3 histone, family 3B (H3.3B) |
| RAPGEF2 | NM_014247 | Rap guanine nucleotide exchange factor (GEF) 2 |
| SMU1 | NM_018225 | smu-1 suppressor of mec-8 and unc-52 homolog (C. elegans) |
| PDE2A | NM_001143839 | phosphodiesterase 2A, cGMP-stimulated |
| TFCP2 | NM_001173452 | transcription factor CP2 |
| SHPK | NM_013276 | sedoheptulokinase |
| PTGER3 | NM_198715 | prostaglandin E receptor 3 (subtype EP3) |
| SLC9A2 | NM_003048 | solute carrier family 9 (sodium/hydrogen exchanger), member 2 |
| DNAJC15 | NM_013238 | DnaJ (Hsp40) homolog, subfamily C, member 15 |
| RASSF5 | NM_182663 | Ras association (RalGDS/AF-6) domain family member 5 |
| PPM1H | NM_020700 | protein phosphatase, Mg2+/Mn2+ dependent, 1H |
| TTYH2 | NM_032646 | tweety homolog 2 (Drosophila) |
| DTX3L | NM_138287 | deltex 3-like (Drosophila) |
| PARD6B | NM_032521 | par-6 partitioning defective 6 homolog beta (C. elegans) |
| ZNF566 | NM_001145343 | zinc finger protein 566 |
| SP4 | NM_003112 | Sp4 transcription factor |
| JOSD1 | NM_014876 | Josephin domain containing 1 |
| RBM12 | NM_001198838 | RNA binding motif protein 12 |
| C9orf152 | NM_001012993 | chromosome 9 open reading frame 152 |
| TTC33 | NM_012382 | tetratricopeptide repeat domain 33 |
| CD59 | NM_000611 | CD59 molecule, complement regulatory protein |
| GATA2 | NM_001145661 | GATA binding protein 2 |
| IRF4 | NM_001195286 | interferon regulatory factor 4 |
| TAP2 | NM_000544 | transporter 2, ATP-binding cassette, sub-family B (MDR/TAP) |
| MAP3K13 | NM_001242314 | mitogen-activated protein kinase kinase kinase 13 |
| WDR20 | NM_001242415 | WD repeat domain 20 |
| ZNF772 | NM_001024596 | zinc finger protein 772 |
| EPB41L1 | NM_012156 | erythrocyte membrane protein band 4.1-like 1 |
| DICER1 | NM_001195573 | dicer 1, ribonuclease type III |

| | | |
|---|---|---|
| KIAA0355 | NM_014686 | KIAA0355 |
| DIP2A | NM_001146116 | DIP2 disco-interacting protein 2 homolog A (Drosophila) |
| GSPT1 | NM_001130006 | G1 to S phase transition 1 |
| KIAA1462 | NM_020848 | KIAA1462 |
| GRIA1 | NM_000827 | glutamate receptor, ionotropic, AMPA 1 |
| ACVR2B | NM_001106 | activin A receptor, type IIB |
| CEBPG | NM_001806 | CCAAT/enhancer binding protein (C/EBP), gamma |
| FLT1 | NM_001159920 | fms-related tyrosine kinase 1 (vascular endothelial growth factor/vascular permeability |
| NR5A2 | NM_003822 | nuclear receptor subfamily 5, group A, member 2 |
| GABRA1 | NM_000806 | gamma-aminobutyric acid (GABA) A receptor, alpha 1 |
| GRIN2A | NM_000833 | glutamate receptor, ionotropic, N-methyl D-aspartate 2A |
| HMGB1 | NM_002128 | high mobility group box 1 |
| NDST1 | NM_001543 | N-deacetylase/N-sulfotransferase (heparan glucosaminyl) 1 |
| FOXK2 | NM_004514 | forkhead box K2 |
| INPP5A | NM_005539 | inositol polyphosphate-5-phosphatase, 40kDa |
| KCNMA1 | NM_001014797 | potassium large conductance calcium-activated channel, subfamily M, alpha member |
| LASP1 | NM_006148 | LIM and SH3 protein 1 |
| SMAD5 | NM_001001419 | SMAD family member 5 |
| MAP6 | NM_207577 | microtubule-associated protein 6 |
| MLLT6 | NM_005937 | myeloid/lymphoid or mixed-lineage leukemia (trithorax homolog, Drosophila); |
| MMP8 | NM_002424 | matrix metallopeptidase 8 (neutrophil collagenase) |
| NKTR | NM_005385 | natural killer-tumor recognition sequence |
| PDGFRA | NM_006206 | platelet-derived growth factor receptor, alpha polypeptide |
| PGR | NM_000926 | progesterone receptor |
| PODXL | NM_001018111 | podocalyxin-like |
| PPP2R5C | NM_001161725 | protein phosphatase 2, regulatory subunit B', gamma |
| PURA | NM_005859 | purine-rich element binding protein A |
| PVRL1 | NM_002855 | poliovirus receptor-related 1 (herpesvirus entry mediator C) |
| PEX2 | NM_000318 | peroxisomal biogenesis factor 2 |
| SFRP1 | NM_003012 | secreted frizzled-related protein 1 |
| SGCD | NM_000337 | sarcoglycan, delta (35kDa dystrophin-associated glycoprotein) |
| SH3BP2 | NM_001122681 | SH3-domain binding protein 2 |
| STRN | NM_003162 | striatin, calmodulin binding protein |
| TLL2 | NM_012465 | tolloid-like 2 |
| SEC62 | NM_003262 | SEC62 homolog (S. cerevisiae) |
| CORO2A | NM_003389 | coronin, actin binding protein, 2A |
| ZMYM2 | NM_001190964 | zinc finger, MYM-type 2 |
| STK24 | NM_001032296 | serine/threonine kinase 24 |
| LAMTOR3 | NM_021970 | late endosomal/lysosomal adaptor, MAPK and MTOR activator 3 |

| | | |
|---|---|---|
| TNKS | NM_003747 | tankyrase, TRF1-interacting ankyrin-related ADP-ribose polymerase |
| SEMA5A | NM_003966 | sema domain, seven thrombospondin repeats (type 1 and type 1-like), transmembrane domain (TM) and short cytoplasmic domain, (semaphorin) 5A |
| MTMR7 | NM_004686 | myotubularin related protein 7 |
| LRRFIP1 | NM_001137550 | leucine rich repeat (in FLII) interacting protein 1 |
| CCPG1 | NM_004748 | cell cycle progression 1 |
| CYTH1 | NM_004762 | cytohesin 1 |
| NTN1 | NM_004822 | netrin 1 |
| PHACTR2 | NM_001100164 | phosphatase and actin regulator 2 |
| KIAA0247 | NM_014734 | KIAA0247 |
| RIMS3 | NM_014747 | regulating synaptic membrane exocytosis 3 |
| LPPR4 | NM_001166252 | lipid phosphate phosphatase-related protein type 4 |
| SV2B | NM_001167580 | synaptic vesicle glycoprotein 2B |
| GJC1 | NM_001080383 | gap junction protein, gamma 1, 45kDa |
| MRVI1 | NM_001098579 | murine retrovirus integration site 1 homolog |
| SLC9A6 | NM_001042537 | solute carrier family 9 (sodium/hydrogen exchanger), member 6 |
| ENDOD1 | NM_015036 | endonuclease domain containing 1 |
| MESDC2 | NM_015154 | mesoderm development candidate 2 |
| KIAA1024 | NM_015206 | KIAA1024 |
| CAMTA1 | NM_015215 | calmodulin binding transcription activator 1 |
| SLC7A11 | NM_014331 | solute carrier family 7 (anionic amino acid transporter light chain, xc- system), member |
| RAB3GAP2 | NM_012414 | RAB3 GTPase activating protein subunit 2 (non-catalytic) |
| FBXO9 | NM_012347 | F-box protein 9 |
| TFCP2L1 | NM_014553 | transcription factor CP2-like 1 |
| A1CF | NM_001198818 | APOBEC1 complementation factor |
| RSBN1 | NM_018364 | round spermatid basic protein 1 |
| LEPROT | NM_001198681 | leptin receptor overlapping transcript |
| KLHL24 | NM_017644 | kelch-like 24 (Drosophila) |
| UBE2R2 | NM_017811 | ubiquitin-conjugating enzyme E2R 2 |
| STRBP | NM_001171137 | spermatid perinuclear RNA binding protein |
| PARVA | NM_018222 | parvin, alpha |
| BCAP29 | NM_001008405 | B-cell receptor-associated protein 29 |
| RAD18 | NM_020165 | RAD18 homolog (S. cerevisiae) |
| CABP4 | NM_145200 | calcium binding protein 4 |
| TWSG1 | NM_020648 | twisted gastrulation homolog 1 (Drosophila) |
| C12orf5 | NM_020375 | chromosome 12 open reading frame 5 |
| KIAA1244 | NM_020340 | KIAA1244 |
| NUFIP2 | NM_020772 | nuclear fragile X mental retardation protein interacting protein 2 |
| CCDC90A | NM_001031713 | coiled-coil domain containing 90A |

| | | |
|---|---|---|
| C10orf54 | NM_022153 | chromosome 10 open reading frame 54 |
| ZNF655 | NM_001009960 | zinc finger protein 655 |
| CDC73 | NM_024529 | cell division cycle 73, Paf1/RNA polymerase II complex component, homolog (S. |
| PODNL1 | NM_001146254 | podocan-like 1 |
| VCPIP1 | NM_025054 | valosin containing protein (p97)/p47 complex interacting protein 1 |
| RAB33B | NM_031296 | RAB33B, member RAS oncogene family |
| SYT15 | NM_031912 | synaptotagmin XV |
| PCGF5 | NM_032373 | polycomb group ring finger 5 |
| PLEKHA8 | NM_001197026 | pleckstrin homology domain containing, family A (phosphoinositide binding specific) |
| ARHGAP19 | NM_001204300 | Rho GTPase activating protein 19 |
| FHDC1 | NM_033393 | FH2 domain containing 1 |
| ZC3H12C | NM_033390 | zinc finger CCCH-type containing 12C |
| SSH2 | NM_033389 | slingshot homolog 2 (Drosophila) |
| KLHL6 | NM_130446 | kelch-like 6 (Drosophila) |
| EFHC1 | NM_001172420 | EF-hand domain (C-terminal) containing 1 |
| RAB3IP | NM_001024647 | RAB3A interacting protein (rabin3) |
| FGD4 | NM_139241 | FYVE, RhoGEF and PH domain containing 4 |
| C1orf96 | NM_145257 | chromosome 1 open reading frame 96 |
| TBC1D20 | NM_144628 | TBC1 domain family, member 20 |
| ICA1L | NM_138468 | islet cell autoantigen 1,69kDa-like |
| EMB | NM_198449 | embigin |
| CD109 | NM_001159587 | CD109 molecule |
| VPS37A | NM_001145152 | vacuolar protein sorting 37 homolog A (S. cerevisiae) |
| NFAM1 | NM_145912 | NFAT activating protein with ITAM motif 1 |
| FAM117B | NM_173511 | family with sequence similarity 117, member B |
| TMEM167A | NM_174909 | transmembrane protein 167A |
| C18orf54 | NM_173529 | chromosome 18 open reading frame 54 |
| ADAMTS15 | NM_139055 | ADAM metallopeptidase with thrombospondin type 1 motif, 15 |
| KIAA2018 | NM_001009899 | KIAA2018 |
| ARL5B | NM_178815 | ADP-ribosylation factor-like 5B |
| SDK1 | NM_152744 | sidekick homolog 1, cell adhesion molecule (chicken) |
| C7orf41 | NM_152793 | chromosome 7 open reading frame 41 |
| NAPEPLD | NM_001122838 | N-acyl phosphatidylethanolamine phospholipase D |
| KCTD20 | NM_173562 | potassium channel tetramerisation domain containing 20 |
| MMS22L | NM_198468 | MMS22-like, DNA repair protein |
| SYT14 | NM_001146261 | synaptotagmin XIV |
| KCTD21 | NM_001029859 | potassium channel tetramerisation domain containing 21 |
| SIAH3 | NM_198849 | seven in absentia homolog 3 (Drosophila) |
| GPIHBP1 | NM_178172 | glycosylphosphatidylinositol anchored high density lipoprotein binding protein 1 |

| | | |
|---|---|---|
| CYP27C1 | NM_001001665 | cytochrome P450, family 27, subfamily C, polypeptide 1 |
| ATXN1L | NM_001137675 | ataxin 1-like |
| FAM73A | NM_198549 | family with sequence similarity 73, member A |
| ILDR2 | NM_199351 | immunoglobulin-like domain containing receptor 2 |
| VGLL3 | NM_016206 | vestigial like 3 (Drosophila) |
| MCART6 | NM_001012755 | mitochondrial carrier triple repeat 6 |
| ZBTB8A | NM_001040441 | zinc finger and BTB domain containing 8A |
| ZNF814 | NM_001144989 | zinc finger protein 814 |
| DDTL | NM_001084393 | D-dopachrome tautomerase-like |
| CASP1 | NM_001223 | caspase 1, apoptosis-related cysteine peptidase (interleukin 1, beta, convertase) |
| ABCB1 | NM_000927 | ATP-binding cassette, sub-family B (MDR/TAP), member 1 |
| RPS27A | NM_001135592 | ribosomal protein S27a |
| TKT | NM_001064 | transketolase |
| TRDN | NM_006073 | triadin |
| RBBP9 | NM_006606 | retinoblastoma binding protein 9 |
| MMRN1 | NM_007351 | multimerin 1 |
| CNTLN | NM_001114395 | centlein, centrosomal protein |
| ZDHHC4 | NM_001134387 | zinc finger, DHHC-type containing 4 |
| UBR7 | NM_175748 | ubiquitin protein ligase E3 component n-recognin 7 (putative) |
| TRPM8 | NM_024080 | transient receptor potential cation channel, subfamily M, member 8 |
| TUBAL3 | NM_001171864 | tubulin, alpha-like 3 |
| ALG13 | NM_001099922 | asparagine-linked glycosylation 13 homolog (S. cerevisiae) |
| ERI2 | NM_001142725 | ERI1 exoribonuclease family member 2 |
| TP53RK | NM_033550 | TP53 regulating kinase |
| LEO1 | NM_138792 | Leo1, Paf1/RNA polymerase II complex component, homolog (S. cerevisiae) |
| DNAJC19 | NM_001190233 | DnaJ (Hsp40) homolog, subfamily C, member 19 |
| COMMD1 | NM_152516 | copper metabolism (Murr1) domain containing 1 |
| MPZL3 | NM_198275 | myelin protein zero-like 3 |
| TMED4 | NM_182547 | transmembrane emp24 protein transport domain containing 4 |
| YTHDF3 | NM_152758 | YTH domain family, member 3 |
| ZNF844 | NM_001136501 | zinc finger protein 844 |
